# Supplementary material for: Translating new evidence into clinical practice: a quasi-experimental controlled before–after study evaluating the effect of a novel outreach mentoring approach on knowledge, attitudes and confidence of health workers providing HIV and infant feeding counselling in South Africa
Source: BMJ Open. 2020 Oct 27;10(10):e034770. doi: 10.1136/bmjopen-2019-034770 (PMC7592306; doi:10.1136/bmjopen-2019-034770)
Supplement: Supplementary data [file bmjopen-2019-034770supp001.pdf]

Version4_ 14August2017

QUESTIONNAIRE FOR HEALTHCARE WORKERS

Goga A, *et al. BMJ Open* 2020; 10:e034770. doi: 10.1136/bmjopen-2019-034770

Version4_ 14August2017

**Section 1. Admin**		
1.1.	Interview Date	☐☐/ ☐☐ ☐ /☐☐☐☐<br>D D M M M Y Y Y Y
1.2.	District	1 OTshwane<br>2 OUgu
1.3.	Sub district	**If Tshwane chosen above:**<br>1 OTshwane 1<br>1 OTshwane 2<br>1 OTshwane 3<br>1 OTshwane 6<br>**If Ugu chosen above:**<br>1 O Omdoni<br>1 O UmZumbe<br>1 O Hibiscus<br>1 O uMuziwabantu
1.4.	Facility	**If Tshwane 1 selected the following clinics can be chosen:**<br>1 O Soshang Block JJ clinic<br>1 O KT Motubatse clinic<br>1 O Boikhutsong clinic<br>1 O Sedilega clinic<br>1 O Shoshanguve 2 clinic<br>1 O Soshang Block TT clinic<br>**If Tshwane 2 selected:**<br>1 O Jubilee gateway clinic<br>1 O Kekanastad clinic<br>1 O Suurman clinic<br>1 O Ramotse clinic<br>1 O Kekana gardens clinic<br>1 O New Eersterus clinic<br>**If Tshwane 3 selected**<br>Atteridgeville Clinic<br>Bophelong Clinic (Tshw 3)<br>Saulsville Clinic

Goga A, *et al. BMJ Open* 2020; 10:e034770. doi: 10.1136/bmjopen-2019-034770

Version4_ 14August2017

		**If Tshwane 6 selected**<br>S Bopape CHC<br>Eersterust CHC<br>Nellmapius Clinic<br><br>**If Omdoni selected**<br>GJ Crooke's Gateway<br>Pennington Clinic<br>Philani Clinic<br>Scottburgh Clinic<br>Umzinto Clinic<br><br>**If UmZumbe selected**<br>• Gqayinyanga clinic<br>• St Faiths clinic<br>• Phungashe clinic<br>• Ntimbankulu clinic<br>• Turton CHC<br>• Ndelu clinic<br><br>**If Hibiscus coast selected:**<br>• Gamalakhe CHC<br>• Southport Clinic<br>• Marburg Clinic<br><br>**If uMuziwabantu selected**<br>• Santombe clinic<br>• Meadowsweet clinic<br>• Mbonwa clinic
1.5.	Health worker number	Assigned number to each participant

Version4_ 14August2017

Section 2. Demographics			
2.1.	What is your date of birth?	□□/□□/□□□□ D D M M Y Y Y Y	
2.2.	Gender	1 O Male	2 O Female
2.3.	What is your role in this clinic?	1 O Lay counsellor or nutritional advisor<br>2. O Enrolled nurse assistant<br>3. O Enrolled nurse<br>4. O Registered nurse<br>5. O Medical degree (MB ChB or equivalent)<br>6. O community health worker (CCG)<br>7 O: dietician<br>8 O: Registered nurse operational manager<br>9 O: other specify below	
2.4.	Other		
2.5.	How long have you been working as a health worker?	1 O less than 1 year<br>2 O 1- <2 years<br>3 O 2- <5 years<br>4 O 5-< 10 years<br>5 O 10 or more years	

Version4_ 14August2017

**Section 3**	**Updated HIV and infant feeding guidelines.**<br>**In this section you will be asked about new infant feeding guidelines adopted in South Africa.**		
3.1	During 2017 have you received any information or training at work about the revised Infant and Young Child Feeding Policy – in the form of a circular, letter, workshop, meeting or lecture	1O Yes	0O No SKIP
3.2	If yes, how did you receive this information?	1. O Circular/letter<br>2. O Meeting<br>3. O Workshop<br>4. O Feedback/information from colleague<br>5. O lecture<br>6. O other	
3.3	Who gave you this information/ training?	1 O District trainer/ DoH staff member<br>2 O Outside/ private company	
3.4	How long was this training?	__________hours	

Version4_ 14August2017

SECTION 4.	**TRAINING Topics**<br>Have you received any training on the following topics (either in-service or formal training).<br><br>**Usuke wathola uqeqesho kulezihloko ezilandelayo?**		
4.1	Did the content of your training include the importance of breastfeeding in preventing common childhood illness such as diarrhoea?	1 ○ Yes	0○ No
4.2	Have you ever had any training about correct positioning and attachment of an infant during breastfeeding?	1 ○ Yes	0○ No
4.3	Have you ever had any training about the management of common breastfeeding problems?	1 ○ Yes	0○ No
4.4	Have you ever had any training about advising a mother about how to provide breastmilk for her baby when she returns to work or school	1 ○ Yes	0○ No
4.5	Have you ever had any training about how to advise a mother about formula feeding safely?	1 ○ Yes	0○ No
4.6	Have you ever had any training about how to advise an HIV infected woman about how to feed her baby?	1 ○ Yes	0○ No
4.7	Have you ever had any training about how to manage breastfeeding problems in HIV infected women (cracked nipples, mastitis etc.)?	1 ○ Yes	5.0 ○ No
4.8	Have you ever had any training about how to assess and support ART adherence for HIV infected women?	1 ○ Yes	0 ○ No
4.9	Have you ever had any training on viral load monitoring?	1 ○ Yes	0 ○ No

Version4_ 14August2017

Section 5	**ACTIVITIES:**<br>***Think carefully about your work in this facility.*** For the activity mentioned consider whether you ever perform this activity and if so how regularly do you perform this activity? If you do not perform this activity at all select the option 'Never'				
5.1	How often do you talk to groups of pregnant women attending the antenatal clinic about infant feeding (group counselling)	1 O one or more times per week	2 O one to three times per month	3 O Less than once a month	4 O Never
5.2	How often do you talk to a pregnant woman individually about her plan for feeding her baby	1 O one or more times per week	2 O one to three times per month	3 O Less than once a month	4 O Never
5.3	How often do you talk to an HIV infected pregnant woman about her plan for feeding her baby	1 O one or more times per week	2 O one to three times per month	3 O Less than once a month	4 O Never
5.4	How often do you assist a mother with breastfeeding within the first hour post delivery	1 O one or more times per week	2 O one to three times per month	3 O Less than once a month	4 O Never
5.5	How often do you talk to a mother about how she is feeding her baby?	1 O one or more times per week	2 O one to three times per month	3 O Less than once a month	4 O Never
5.6	How often do you observe a mother breastfeeding during a clinic or home visit	1 O one or more times per week	2 O one to three times per month	3 O Less than once a month	4 O Never
5.7	How often do you talk to a mother about positioning and attachment of the baby during breastfeeding	1 O one or more times per week	2 O one to three times per month	3 O Less than once a month	4 O Never
5.8	How often do you talk to an HIV infected mother about managing a breastfeeding problem (e.g. cracked nipples, baby crying all the time, mother says she does not have enough milk)	1 O one or more times per week	2 O one to three times per month	3 O Less than once a month	4 O Never
5.9	How often do you talk to a mother about how to maintain breastfeeding when away from the baby (going back to school or work)	1 O one or more times per week	2 O one to three times per month	3 O Less than once a month	4 O Never
5.10	How often do you talk to an HIV infected breastfeeding mother about taking ARVs	1 O one or more times per week	2 O one to three times per month	3 O Less than once a month	4 O Never

Version4_ 14August2017

SECTION 6	INFANT FEEDING KNOWLEDGE			
please state whether the statement is true or false or you do not know				
	**Statement**	**True**	**False**	**Do not know**
6.1	Exclusive breastfeeding is the recommended infant feeding method for ALL infants aged 0-6 months in SA, regardless of mother's HIV status	1 ○	2 ○	3 ○
6.2	Giving any formula milk during the first six months of life increases the risk of death from diarrhoea and/or pneumonia	1 ○	2 ○	3 ○
6.3	Continued breastfeeding for 2 years is the recommended infant method in SA for ALL children, regardless of mother's HIV status	1 ○	2 ○	3 ○
6.4	Mothers living with HIV who are receiving antiretroviral treatment and are virally suppressed should be advised not to breastfeed their infants	1 ○	2 ○	3 ○
6.5	When an HIV infected mother is ready to add complementary feeds she should stop breastfeeding rapidly over a 24hour period	1 ○	2 ○	3 ○
6.6	If an HIV exposed baby is receiving both breastmilk and formula milk, the mother should chose to either breastfeeding or formula feeding if she is adherent to ART	1 ○	2 ○	3 ○
6.7	A mother who is working and giving formula milk should mix the milk herself and leave for the carer to give during the day	1 ○	2 ○	3 ○
6.8	An HIV positive mother who is virally suppressed on antiretroviral treatment should breastfeed her child rather than not breastfeed to improve the child's survival	1 ○	2 ○	3 ○
6.9	When sterilising feeding bottles cover the bottles with water in a saucepan and place on the heat. As soon as the water boils remove from heat and leave the bottle in the water until completely cool	1 ○	2 ○	3 ○
6.10	In South Africa, the leading cause of death amongst children under 5 is pneumonia	1 ○	2 ○	3 ○
6.11	In South Africa, HIV infected women who are breastfeeding should be supported to adhere to antiretroviral treatment and should be counselled and supported to exclusively breastfeed their infants for the first six months of life whilst maintaining an undetectable viral load	1 ○	2 ○	3 ○
6.12	A baby under 4 months should be given soft porridge once he/she seems hungry	1 ○	2 ○	3 ○

Goga A, *et al. BMJ Open* 2020; 10:e034770. doi: 10.1136/bmjopen-2019-034770

Version4_ 14August2017

6.13	A mother living with HIV and adherent to antiretroviral treatment cannot exclusively breastfeed her 4-month old infant because she is working. It is better for this mother to give formula during the day and breastfeed at night rather than giving no breast milk at all	1 ○	2 ○	3 ○
6.14	An HIV exposed baby who is exclusively breastfeeding should be given some water when the weather is very hot	1 ○	2 ○	3 ○
6.15	If a baby has a positive PCR (HIV test) at birth the mother should stop breastfeeding if this is affordable and feasible in her situation	1 ○	2 ○	3 ○
6.16	Giving a baby expressed breastmilk is not as good as breastfeeding	1 ○	2 ○	3 ○
6.17	If a mother misses 2 doses of her ART in one month, she should be classified as a treatment failure	1 ○	2 ○	3 ○
6.18	An HIV positive mother who has cracked nipples should continue to breastfeed unless they are bleeding	1 ○	2 ○	3 ○
6.19	A mother who has missed 6 tablets of FDC in one month is considered to be poorly adherent and should stop breastfeeding immediately	1 ○	2 ○	3 ○
6.20	There are long term health benefits of breastfeeding for mother and child that last beyond the breastfeeding period	1 ○	2 ○	3 ○
6.21	In South Africa, HIV infected women who are breastfeeding should be supported to adhere to antiretroviral treatment and should introduce complementary foods around 6 months and be supported to continue breastfeeding for at least two years.	1 ○	2 ○	3 ○
6.22	It is safe to give the baby expressed breastmilk that has been kept outside the fridge for 8 hours	1 ○	2 ○	3 ○

Goga A, *et al. BMJ Open* 2020; 10:e034770. doi: 10.1136/bmjopen-2019-034770

Version4_ 14August2017

**SECTION 7.**		**INFANT FEEDING ATTITUDE**<br>Please state whether you completely disagree, disagree, neutral, agree or completely agree with the statement				
			Completely disagree	Disagree	Neutral	Agree	Completely agree
7.1	There have been so many changes to the infant feeding guidelines and breastfeeding guidelines that I am confused about what to tell mothers who are HIV infected about	1 ○	2 ○	3 ○	4 ○	5 ○
7.2	When a baby cries all the time it is usually because the baby is hungry and needs more food than just breastmilk	1 ○	2 ○	3 ○	4 ○	5 ○
7.3	Exclusive breastfeeding in the first 6 months of life is the best choice for all mothers and babies in South Africa	1 ○	2 ○	3 ○	4 ○	5 ○
7.4	For an HIV exposed infant any breastfeeding is better than no breastfeeding at all, as long as the mother is virally suppressed and on antiretroviral therapy	1 ○	2 ○	3 ○	4 ○	5 ○
7.5	The benefits of breastfeeding for protecting children from illness such as diarrhoea and pneumonia outweighs the risk of acquiring HIV if the mother is on antiretroviral treatment	1 ○	2 ○	3 ○	4 ○	5 ○
7.6	I feel that an HIV infected mother who has not disclosed to her partner is at high risk of non-adherence to ART and should stop breastfeeding as soon as [illegible]	1 ○	2 ○	3 ○	4 ○	5 ○
7.7	I should support all mothers, regardless of HIV status, to continue breastfeeding until 2 years, as long as HIV infected women are virally suppressed	1 ○	2 ○	3 ○	4 ○	5 ○
7.8	I should advise an HIV positive virally suppressed mother who has cracked and bleeding nipples to temporarily stop breastfeeding	1 ○	2 ○	3 ○	4 ○	5 ○
7.9	HIV exposed babies who are PCR negative must stop breastfeeding as soon as possible	1 ○	2 ○	3 ○	4 ○	5 ○

Version4_ 14August2017

7.10	Formula feeding is the best choice for mothers living in good socioeconomic circumstances who are going back to work	1 O	2 O	3 O	4 O	5 O
7.11	For an HIV positive mother on antiretroviral treatment and virally suppressed mixed feeding is better than not breastfeeding at all	1 O	2 O	3 O	4 O	5 O
7.12	Exclusive breastfeeding for six months is an achievable goal for the majority of mothers	1 O	2 O	3 O	4 O	5 O
7.13	It is safer for HIV positive mothers to breastfeed than to formula feed	1 O	2 O	3 O	4 O	5 O
7.14	In our community working mothers can successfully maintain exclusive breast feeding while going to work	1 O	2 O	3 O	4 O	5 O
7.15	An HIV positive mother who is on ART and not virally suppressed and is mixed feeding is putting her child at risk of acquiring HIV	1 O	2 O	3 O	4 O	5 O
7.16	It is very difficult for mothers to express breastmilk while they are at work or school	1 O	2 O	3 O	4 O	5 O
7.17	If an HIV positive mother can afford to buy formula it is better for her to formula feed her baby	1 O	2 O	3 O	4 O	5 O
7.18	Promoting breastfeeding for two years for HIV exposed infants is a risk because mothers will be unable to maintain good ART adherence for that long	1 O	2 O	3 O	4 O	5 O
7.19	In South Africa it is possible to improve exclusive breastfeeding rates	1 O	2 O	3 O	4 O	5 O
7.20	There are exceptional circumstances where an HIV positive mother would be advised not to breastfeed, such as failure of 2nd or 3rd line ART treatment, but these are not common	1 O	2 O	3 O	4 O	5 O
7.21	Formula feeding is more convenient for a mother than breastfeeding	1 O	2 O	3 O	4 O	5 O

Goga A, *et al. BMJ Open* 2020; 10:e034770. doi: 10.1136/bmjopen-2019-034770

Version4_ 14August2017

SECTION 8.	INFANT FEEDING COUNSELLING CONFIDENCE<br><br>For each activity below, please indicate how confident you feel to undertake each activity. Do you feel "Not at all confident", "Not very confident", "Somewhat confident" or "Very confident".				
		Not at all confident	Not very confident	Confident	Very confident
8.1	How confident do you feel about counselling an HIV positive pregnant woman about how she will feed her baby	1 ○	2 ○	3 ○	4 ○
8.2	How confident do you feel about giving information about the risks and benefits of breastfeeding to an HIV infected mother	1 ○	2 ○	3 ○	4 ○
8.3	How confident do you feel about assessing whether there is good positioning and attachment during breastfeeding	1 ○	2 ○	3 ○	4 ○
8.4	How confident do you feel about advising an HIV positive mother about how to continue to breastfeed her baby when she	1 ○	2 ○	3 ○	4 ○
8.5	How confident do you feel about advising an HIV infected mother who is virally suppressed who is mixed feeding her infant	1 ○	2 ○	3 ○	4 ○
8.6	How confident do you feel about advising an HIV infected mother to continue breastfeeding for two years	1 ○	2 ○	3 ○	4 ○
8.7	How confident do you feel about advising an HIV infected mother about how to stop breastfeeding	1 ○	2 ○	3 ○	4 ○
8.8	How confident do you feel about advising an HIV positive mother about starting complementary feeds	1 ○	2 ○	3 ○	4 ○
8.9	How confident do you feel about assessing ART compliance in an HIV positive mother	1 ○	2 ○	3 ○	4 ○
8.10	How confident do you feel about identifying when an HIV positive mother is not adhering to her ART treatment	1 ○	2 ○	3 ○	4 ○
8.11	How confident do you feel about reassuring a mother living with HIV who is virally suppressed that a shorter duration of breastfeeding is better than never initiating breastfeeding	1 ○	2 ○	3 ○	4 ○
8.12	How confident do you feel about explaining the risks of HIV transmission through breastmilk to an HIV infected mother with high viral load	1 ○	2 ○	3 ○	4 ○

Version4_ 14August2017

8.13	How confident do you feel about assisting a mother with HIV to safely formula feed her baby	1 O	2 O	3 O	4 O
8.14	How confident do you feel about advising an HIV infected mother who is exclusively breastfeeding and has cracked nipples with bloody milk about how to feed her baby	1 O	2 O	3 O	4 O
8.15	How confident do you feel about using the guidelines for safe replacement feeding when you counsel a mother who is not adherent to ART and has a viral load above 1000 copies/ml	1 O	2 O	3 O	4 O
8.16	How confident do you feel about advising an HIV infected mother who is exclusively breastfeeding and has defaulted from her ART about how to feed her baby	1 O	2 O	3 O	4 O
8.17	How confident do you feel about explaining to a mother about expressing and storing milk	1 O	2 O	3 O	4 O
8.18	How confident do you feel about managing poor ART compliance in an HIV infected breastfeeding mother	1 O	2 O	3 O	4 O
8.19	A mother is not adherent to ART and her last viral load is 1000 copies per ml. How confident do you feel about counselling her about feeding her infant?	1 O	2 O	3 O	4 O

Goga A, *et al. BMJ Open* 2020; 10:e034770. doi: 10.1136/bmjopen-2019-034770
