# Supplementary material for: Translating new evidence into clinical practice: a quasi-experimental controlled before–after study evaluating the effect of a novel outreach mentoring approach on knowledge, attitudes and confidence of health workers providing HIV and infant feeding counselling in South Africa
Source: BMJ Open. 2020 Oct 27;10(10):e034770. doi: 10.1136/bmjopen-2019-034770 (PMC7592306; doi:10.1136/bmjopen-2019-034770)
Supplement: Supplementary data [file bmjopen-2019-034770supp002.pdf]

Supplementary Figure 1: Prior training reported by participants at baseline (intervention versus comparison groups)

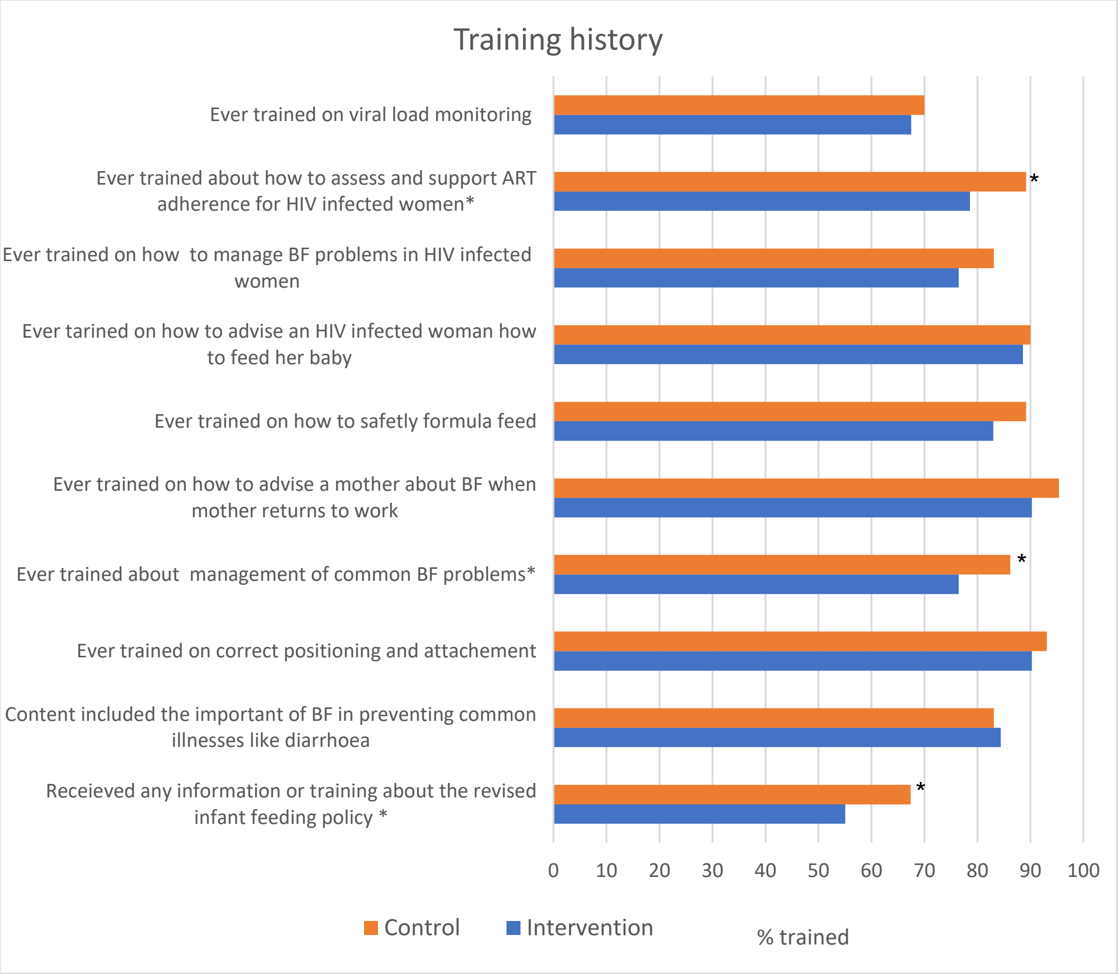

*p<0.05

Abbreviations: ART= antiretroviral therapy; BF= breastfeeding
