# Supplementary material for: Translating new evidence into clinical practice: a quasi-experimental controlled before–after study evaluating the effect of a novel outreach mentoring approach on knowledge, attitudes and confidence of health workers providing HIV and infant feeding counselling in South Africa
Source: BMJ Open. 2020 Oct 27;10(10):e034770. doi: 10.1136/bmjopen-2019-034770 (PMC7592306; doi:10.1136/bmjopen-2019-034770)
Supplement: Supplementary data [file bmjopen-2019-034770supp003.pdf]

Supplementary Table 1: Attitude scores of health workers at baseline and follow-up

Attitude statements	Number (%) who agreed or strongly agreed with the statement at baseline			Number (%) who agreed or strongly agreed with the statement at follow-up		
	Intervention (n=289)	Comparison (n=131)	p-value *	Intervention (n=252)	Comparison (n=114)	p-value*
Significant improvements between intervention and comparison groups at follow-up						
There have been so many changes to the infant feeding guidelines and breastfeeding guidelines however I am NOT confused about what to tell mothers who are HIV-infected about breastfeeding	155 (53.6)	74 (56.9)	059	175 (69.4)	65 (57.0)	0.02
For an HIV-exposed infant any breastfeeding is better than no breastfeeding at all, as long as the mother is virally suppressed and on antiretroviral therapy	189 (65.4)	79 (61.2)	0.31	196 (77.8)	74 (64.9)	0.01
When a baby cries all the time it is NOT usually because the baby is hungry and needs more food than just breastmilk	260 (90.0)	107 (83.0)	0.02	230 (91.3)	91(79.8)	<0.01
Exclusive breastfeeding in the first 6 months of life is the best choice for all mothers and babies in South Africa	256 (88.6)	114 (88.4)	0.65	239 (94.8)	98 (86.0)	<0.01
The benefits of breastfeeding for protecting children from illness such as diarrhoea and pneumonia outweighs the risk of acquiring HIV if the mother is on antiretroviral treatment	224 (77.5)	93 (72.1)	0.15	218 (86.5)	87 (76.3)	0.02
I feel that an HIV-infected mother who has not disclosed to her partner is NOT at high risk of non-adherence to ART and should NOT stop breastfeeding as soon as possible	128 (44.3)	51 (39.5)	0.30	137 (54.4)	46 (40.4)	0.01
I should support all mothers, regardless of HIV status, to continue breastfeeding until 2 years, as long as HIV-infected women are virally suppressed	237 (82.0)	101 (78.3)	0.24	237 (94.1)	87 (76.3)	<0.01
I should NOT advise an HIV-positive virally suppressed mother who has cracked and bleeding nipples to temporarily stop breastfeeding	80 (27.7)	40 (31.3)	0.55	97 (38.5)	24 (21.1)	<0.01
Formula feeding is NOT the best choice for mothers living in good socio-economic circumstances who are going back to work	202 (70.0)	95 (74.2)	0.58	198 (78.6)	74 (64.9)	<0.01
It is safer for HIV-positive mothers to breastfeed than to formula feed	231 (79.9)	89 (69.5)	0.63	226 (89.7)	85 (74.6)	<0.01
It is NOT very difficult for mothers to express breastmilk while they are at work or school	164 (53.0)	57 (44.5)	0.01	168 (67.1)	51 (44.7)	<0.01
If an HIV-positive mother can afford to buy formula it is NOT better for her to formula feed her baby	170 (58.8)	83 (64.9)	0.38	185 (73.4)	68 (59.7)	<0.01
Promoting breastfeeding for two years for HIV-exposed infants is NOT a risk because	197 (68.2)	85 (66.4)	0.51	210 (83.3)	79 (69.3)	<0.01

Goga A, *et al. BMJ Open* 2020; 10:e034770. doi: 10.1136/bmjopen-2019-034770

mothers will be able to maintain good ART adherence for that long						
Low levels of knowledge (<80%) at baseline in both groups - no significant differences between intervention and comparison groups at follow-up [concept that this relates to]						
For an HIV-positive mother on antiretroviral treatment and virally suppressed mixed feeding is better than not breastfeeding at all	39 (13.5)	15 (11.7)	0.56	57 (22.6)	16 (14.0)	0.06
In our community working mothers can successfully maintain exclusive breast feeding while going to work	219 (75.8)	89 (69.5)	0.09	208 (82.5)	92 (80.7)	0.67
There are exceptional circumstances where an HIV-positive mother would be advised not to breastfeed, such as failure of 2nd or 3rd line ART treatment, but these are not common	225 (77.9)	93 (72.7)	0.13	201 (79.7)	87 (76.3)	0.46
High levels of knowledge (≥80%) at baseline in both groups – no significant differences between intervention and comparison groups at follow-up						
HIV-exposed babies who are PCR negative must NOT stop breastfeeding as soon as possible	238 (82.4)	104 (81.3)	0.47	221 (87.7)	95 (83.3)	0.26
Exclusive breastfeeding for six months is an achievable goal for the majority of mothers	249 (86.2)	105 (82.0)	0.11	227 (90.1)	96 (84.1)	0.11
An HIV-positive mother who is on ART and not virally suppressed and is mixed feeding is putting her child at risk of acquiring HIV	256 (88.6)	109 (85.2)	0.13	219 (86.9)	97 (85.1)	0.64
In South Africa it is possible to improve exclusive breastfeeding rates	244 (84.7)	108 (84.4)	0.61	230 (91.3)	97 (85.1)	0.08
Formula feeding is NOT more convenient for a mother than breastfeeding	253 (87.5)	109 (85.2)	0.23	211 (83.7)	100(87.7)	0.32
**Number (%) participants whose attitude was to at least agree (Attitude score ≥84)***	**71 (24.6)**	**23 (17.9)**	**0.12**	**123 (49.2)**	**27 (24.1)**	**<0.01**
**Mean attitude score out of 105 (95% CI)**	**76.9 (75.9 – 77.9)**	**75.0 (73.0 – 77.0)**	**0.07**	**82.7 (81.6 – 83.8)**	**76.8 (75.0 – 78.5)**	**<0.01**

*84 was the minimum score obtainable if a participant at least agreed with all statements

Supplementary Table 2: Confidence statements of health workers at baseline and follow-up

Confidence statements	Number (%) who felt confident or very confident at baseline			Number (%) who felt confident or very confident at follow-up		
	Intervention (n=289)	Comparison (n=131)	p-value	Intervention (n=252)	Comparison (n=114)	p-value
Significant improvements between intervention and comparison groups at follow-up						
How confident do you feel about counselling an HIV-positive pregnant woman about how she will feed her baby	265 (91.7)	116 (88.6)	0.30	240 (95.2)	100 (87.7)	0.01
How confident do you feel about advising an HIV-positive mother about how to continue to breastfeed her baby when she returns to work or school	258 (89.3)	117 (89.3)	0.99	242 (96.0)	101 (88.6)	<0.01
How confident do you feel about advising an HIV-infected mother to continue breastfeeding for two years	216 (74.7)	105 (80.2)	0.23	236 (93.6)	93 (81.6)	<0.01
How confident do you feel about assessing ART compliance in an HIV-positive mother	240 (83.1)	111 (84.7)	0.67	230 (91.3)	94 (82.5)	0.02
How confident do you feel about advising an HIV-infected mother who is exclusively breastfeeding and has cracked nipples with bloody milk about how to feed her baby	196 (67.8)	84 (64.1)	0.45	201 (79.8)	80 (70.2)	0.04
Low levels of knowledge (<80%) at baseline in both groups - no significant differences between intervention and comparison groups at follow-up [concept that this relates to]						
How confident do you feel about advising an HIV-infected mother about how to stop breastfeeding	214 (74.1)	89 (67.9)	0.20	188 (74.6)	86 (75.4)	0.87
How confident do you feel about identifying when an HIV-positive mother is not adhering to her ART treatment	224 (77.5)	104 (79.4)	0.67	218 (86.5)	90 (79.0)	0.07
How confident do you feel about reassuring a mother living with HIV who is virally suppressed that a shorter duration of breastfeeding is better than never initiating breastfeeding	227 (78.5)	103 (78.6)	0.99	215 (85.3)	96 (84.2)	0.78
How confident do you feel about assisting a mother with HIV to safely formula feed her baby	212 (73.4)	102 (77.9)	0.33	191 (75.8)	95 (83.3)	0.11
How confident do you feel about using the guidelines for safe replacement feeding when you counsel a mother who is not adherent to ART and has a viral load above 1000 copies/ml	191 (66.1)	99 (75.6)	0.05	185 (73.4)	75 (65.8)	0.14
How confident do you feel about advising an HIV-infected mother who is exclusively breastfeeding and has defaulted from her ART about how to feed her baby	205 (70.9)	97 (74.1)	0.51	189 (75.0)	80 (70..2)	0.33
How confident do you feel about managing poor ART compliance in an HIV-infected breastfeeding mother	215 (74.4)	104 (79.4)	0.27	209 (82.9)	89 (78.1)	0.27

Goga A, *et al. BMJ Open* 2020; 10:e034770. doi: 10.1136/bmjopen-2019-034770

A mother is not adherent to ART and her last viral load is 1000 copies per ml. How confident do you feel about counselling her about feeding her infant?	199 (68.9)	98 (74.8)	0.21	198 (78.6)	80 (70.2)	0.08
High levels of knowledge (≥80%) at baseline in both groups – no significant differences between intervention and comparison groups at follow-up						
How confident do you feel about giving information about the risks and benefits of breastfeeding to an HIV-infected mother	268 (92.7)	120 (91.6)	0.67	238 (94.4)	102 (89.5)	0.09
How confident do you feel about assessing whether there is good positioning and attachment during breastfeeding	263 (91.0)	125 (95.4)	0.11	243(96.4)	107 (93.9)	0.27
How confident do you feel about advising an HIV-infected mother who is virally suppressed who is mixed feeding her infant	243 (84.1)	113 (86.3)	0.57	226 (89.7)	99 (86.8)	0.43
How confident do you feel about advising an HIV-positive mother about starting complementary feeds	251 (86.9)	115 (87.8)	0.79	229 (90.9)	103 (90.4)	0.87
How confident do you feel about explaining the risks of HIV transmission through breastmilk to an HIV-infected mother with high viral load	247 (85.5)	114 (87.0)	0.67	220 (87.3)	105 (92.1)	0.17
How confident do you feel about explaining to a mother about expressing and storing milk	269 (93.1)	124 (94.7)	0.54	237 (94.1)	109 (95.6)	0.54
**Number (%) participants who were confident or very confident (Sum Score ≥ 57)**	**164 (56.8)**	**86 (67.2)**	**0.09**	**175 (70.0)**	**72 (64.3)**	**0.28**
**Mean confidence score out of 76 (95% CI)**	**59.1 (58.0-60.2)**	**59.1 (57.0-61.3)**	**1.0**	**61.2 (60.8-63.1)**	**59.9 (58.1-61.7)**	**0.05**

Goga A, *et al. BMJ Open* 2020; 10:e034770. doi: 10.1136/bmjopen-2019-034770
