# Supplementary material for: Translating new evidence into clinical practice: a quasi-experimental controlled before–after study evaluating the effect of a novel outreach mentoring approach on knowledge, attitudes and confidence of health workers providing HIV and infant feeding counselling in South Africa
Source: BMJ Open. 2020 Oct 27;10(10):e034770. doi: 10.1136/bmjopen-2019-034770 (PMC7592306; doi:10.1136/bmjopen-2019-034770)
Supplement: Author's manuscript [file bmjopen-2019-034770.draft_revisions.pdf]

BMJ Open

BMJ Open is committed to open peer review. As part of this commitment we make the peer review history of every article we publish publicly available.

When an article is published we post the peer reviewers' comments and the authors' responses online. We also post the versions of the paper that were used during peer review. These are the versions that the peer review comments apply to.

The versions of the paper that follow are the versions that were submitted during the peer review process. They are not the versions of record or the final published versions. They should not be cited or distributed as the published version of this manuscript.

BMJ Open is an open access journal and the full, final, typeset and author-corrected version of record of the manuscript is available on our site with no access controls, subscription charges or pay-per-view fees (http://bmjopen.bmj.com).

If you have any questions on BMJ Open's open peer review process please email info.bmjopen@bmj.com

BMJ Open

Translating new evidence into clinical practice: the effect of a novel outreach mentoring approach on the knowledge, attitudes and confidence of health workers providing HIV and infant feeding counselling in South Africa

Journal:	*BMJ Open*
Manuscript ID	bmjopen-2019-034770
Article Type:	Original research
Date Submitted by the Author:	18-Oct-2019

Complete List of Authors:	Goga, Ameena; Medical Research Council of South Africa, Health Systems Research Unit and HIV Prevention Research Unit; University of Pretoria Faculty of Health Sciences, Paediatrics<br>Doherty, Tanya; South African Medical Research Council<br>Manda, Samuel; South African Medical Research Council<br>Nkwenika, Tshifhiwa; SAMRC<br>Haskins, Lyn; University of KwaZulu-Natal<br>John, Vaughn; University of KwaZulu-Natal, School of Education<br>Engebretsen, Ingunn; University of Bergen Faculty of Medicine and Dentistry, Centre for International Health<br>Feucht, Ute; University of Pretoria<br>Dhansay, Ali; Burden of Disease Research Unit<br>Rollins, Nigel; World Health Organization, Department of Maternal, Newborn, Child and Adolescent Health<br>Kroon, Max; University of Cape Town Faculty of Health Sciences, Neonatology<br>Sanders, David; University of the Western Cape, South Africa , School of Public Health<br>Kauchali, Shuaib; South African National Department of Health<br>Tylleskär, Thorkild; Universitetet i Bergen, Centre for International health<br>Horwood, Christiane ; University of KwaZulu-Natal,

Keywords:	Public health < INFECTIOUS DISEASES, HIV & AIDS < INFECTIOUS DISEASES, Community child health < PAEDIATRICS

SCHOLARONE™
Manuscripts

I, the Submitting Author has the right to grant and does grant on behalf of all authors of the Work (as defined in the below author licence), an exclusive licence and/or a non-exclusive licence for contributions from authors who are: i) UK Crown employees; ii) where BMJ has agreed a CC-BY licence shall apply, and/or iii) in accordance with the terms applicable for US Federal Government officers or employees acting as part of their official duties; on a worldwide, perpetual, irrevocable, royalty-free basis to BMJ Publishing Group Ltd ("BMJ") its licensees and where the relevant Journal is co-owned by BMJ to the co-owners of the Journal, to publish the Work in this journal and any other BMJ products and to exploit all rights, as set out in our licence.

The Submitting Author accepts and understands that any supply made under these terms is made by BMJ to the Submitting Author unless you are acting as an employee on behalf of your employer or a postgraduate student of an affiliated institution which is paying any applicable article publishing charge ("APC") for Open Access articles. Where the Submitting Author wishes to make the Work available on an Open Access basis (and intends to pay the relevant APC), the terms of reuse of such Open Access shall be governed by a Creative Commons licence – details of these licences and which Creative Commons licence will apply to this Work are set out in our licence referred to above.

Other than as permitted in any relevant BMJ Author's Self Archiving Policies, I confirm this Work has not been accepted for publication elsewhere, is not being considered for publication elsewhere and does not duplicate material already published. I confirm all authors consent to publication of this Work and authorise the granting of this licence.

For peer review only

Translating new evidence into clinical practice: the effect of a novel outreach mentoring approach on the knowledge, attitudes and confidence of health workers providing HIV and infant feeding counselling in South Africa

Ameena Goga[1, 2, 3], Tanya Doherty[1,4,5], Samuel Manda[6, 7], Tshifhiwa Nkwenika[6], Lyn Haskins[8], Vaughn John[9], Ingunn M.S. Engebretsen[10], Ute Feucht[2, 11,12, 13], Ali Dhansay[14, 15] Nigel Rollins[16], Max Kroon[17, 18], David Sanders[4,19], Shuaib Kauchali[20], Thorkild Tylleskar[10,] Christiane Horwood[8]

Affiliations

[1]Health Systems Research Unit, South African Medical Research Council, South Africa
[2]Department of Paediatrics, University of Pretoria, South Africa
[3]HIV Prevention Research Unit, South African Medical Research Council, South Africa
[4]School of Public Health, University of the Western Cape, South Africa
[5]School of Public Health, University of the Witwatersrand
[6]Biostatistics Unit, South African Medical Research Council, South Africa
[7]Department of Statistics, University of Pretoria, South Africa
[8]Centre for Rural Health, University of KwaZulu-Natal, South Africa
[9]School of Education, University of KwaZulu Natal, Pietermaritzburg, South Africa
[10]Centre for International Health, University of Bergen, Norway
[11]Tshwane District Health Services, Gauteng Department of Health, city? South Africa
[12]Research Centre for Maternal, Fetal, Newborn and Child Health Care Strategies, University of Pretoria, Pretoria, South Africa
[13]Maternal and Infant Health Care Strategies Research Unit, South African Medical Research Council, South Africa
[14]Division of Human Nutrition and Department of Paediatrics and Child Health, Stellenbosch University, South Africa.
[15]Burden of Disease Unit, South African Medical Research Council, South Africa
[16]Department of Maternal, Newborn, Child and Adolescent Health, World Health Organization, Geneva, Switzerland
[17]Department of Neonatology, Faculty of Health Sciences, University of Cape Town
[18]Mowbray Maternity Hospital, Cape Town, South Africa
[19]Department of Paediatrics and Child Health, University of Cape Town, South Africa
[20]National Department of Health, South Africa

Corresponding author: Ameena.Goga@mrc.ac.za

For peer review only

Abstract

Objectives: We report the effectiveness of a theory-driven, facility-based mentoring approach to disseminate updated HIV and infant feeding guidelines.
Design: A quasi-experimental controlled before-after study.
Setting: Primary health care clinics were randomly selected (n=24 intervention, n=12 comparison) from two districts, South Africa.
Participants: All health workers (HW) providing infant feeding counselling in each intervention clinic were invited to participate.
Interventions: three 1-2 hour, on-site workshops were conducted over 3-6 weeks.
Primary outcome measures: a) knowledge scores: 22 statements each scored 1 if correct or 0; b) attitude scores: 21 statements with 5 possible responses: completely disagree (1), disagree (2), neutral (3), agree (4) and completely agree (5); c) confidence scores: 19 statements with 4 possible responses: not at all confident (1), not confident (2), confident (3) and very confident (4). Analysis was at the HW level, with clinic as a random effect to adjust for clustering. Data were analysed using STATA/SE version 15.
Results: In intervention and comparison sites, respectively: 289 and 131 baseline and 253 and 114 follow-up interviews were conducted. At baseline, the proportion of participants previously trained in HIV and infant feeding was significantly higher in comparison clinics. At follow-up, the mean score for the 22 knowledge questions was 15.2 (69%) in comparison and 17.2 (78.2%) in intervention sites ($p<0.001$). Knowledge scores among HW who attended 3 workshops was significantly better ($p<0.0001$) compared with HW who attended <3 workshops. At follow-up, the mean attitude and confidence scores towards breastfeeding were significantly better in intervention sites versus comparison sites ($p<0.001$ and $p=0.05$, respectively).
Conclusion: A participatory, low intensity on-site mentoring programme improved HW's knowledge, attitudes and confidence more than standard training to provide infant feeding counselling. Further research is required to evaluate the effectiveness, feasibility and sustainability of implementing such a mentoring approach at scale.

Strengths and limitations of the study:

1. Fieldwork was conducted in two geographically and historically different provinces, facilitating generalisability of results.
2. The intervention was participatory, low intensity, on-site and integrated into routine facilities.
3. Several types of analyses were conducted which all yielded congruent results.
4. However, limitations were that we purposively selected districts for inclusion; we could not control for previous breastfeeding experience as we did not gather these data; the follow-up evaluation was done 3 months after the intervention; thus, we were only able to measure short term benefits, and we did not measure the effect of improved knowledge, attitudes and confidence on actual infant feeding practices.
5. The finding that knowledge scores at follow-up of participants who attended 3 workshops compared with knowledge scores at follow-up of participants who attended less than 3 workshops was significantly better, may simply reflect better motivation amongst attendees of more workshops, rather than the effect of the workshops themselves. We could not tease out these effects.

For peer review only - http://bmjopen.bmj.com/site/about/guidelines.xhtml

Funding statement

This study was funded by the World Health Organisation (Ref: 2017/712509-0). Tanya Doherty receives a small grant from the National Research Foundation, South Africa. Ameena Goga and Tanya Doherty's time was funded by the South African Medical Research Council. The publication of paper was funded by the South African Medical Research Council.

Competing interests statement

No author has declared any competing interests.

For peer review only

Introduction

Policies and clinical practice guidelines are updated regularly to ensure that health care is guided by the most recent evidence. In the area of HIV and infant feeding, guidelines have been updated frequently as evidence emerged, but effective dissemination of these guidelines has lagged behind. Multi-component dissemination strategies, which aim to increase the reach, ability and motivation of health workers, are more effective than one strategy alone.[1] However, the standard of evidence to guide dissemination strategies is low as studies are few; additionally, most studies have been conducted in high income countries, and results may not apply to low-middle income countries, which have different needs and challenges.[1] We examined how HIV and infant feeding guidelines, which have been updated over the past two decades to align with new evidence, can be effectively disseminated in a middle income setting with high HIV prevalence.

The benefits of breastfeeding in all settings, and particularly in low-middle income settings with high HIV prevalence, are undisputed.[2 3] In 2010, the World Health Organisation (WHO) issued HIV and infant feeding recommendations, in support of six to 12 months of breastfeeding under antiretroviral cover.[4] This followed a period when, in the absence of evidence for interventions such as triple antiretroviral therapy (ART) to prevent postnatal transmission, the avoidance of breastfeeding was recommended if specific conditions conducive to the safe use of replacement feeds could be met. In 2009, evidence was published that both maternal triple ART and infant antiretroviral prophylaxis greatly reduce breast milk HIV transmission risk.[5 6] In July 2016, the WHO guidelines on HIV and infant feeding were updated to recommend exclusive breastfeeding (EBF) for the first six months of life, with the introduction of appropriate complementary foods thereafter and continued breastfeeding for at least 12 to 24 months amongst HIV-negative mothers and mothers living with (MLHIV) while being fully supported for ART adherence, on the assumption that they will be virally suppressed.[7] In June 2017, these recommendations were adopted in South Africa, thus necessitating updates for health workers. Many studies on the uptake of breastfeeding recommendations have focused on mothers, assessing the effect of counselling versus standard education on breastfeeding practices.[8] A few studies have investigated the impact of staff training on HIV and infant feeding guideline implementation.[9] These demonstrated that interventions and training that aim to develop HW's capacity of can significantly improve their skills, self-efficacy and confidence to counsel, support and promote breastfeeding among MLHIV.[8] Consequently, a key question was: What learning approach could best develop health care worker capacity and confidence to implement the updated HIV and

For peer review only - http://bmjopen.bmj.com/site/about/guidelines.xhtml

infant feeding guideline, using a methodology that was sustainable and feasible to implement at scale. Pedagogical research highlights the advantage of participatory training compared with standard didactic teaching for improving health worker skills.[10 11] Thus, we sought to determine whether a participatory outreach mentorship approach to disseminate the updated HIV and infant feeding guidelines, using simple low-technology activities, improves the knowledge, attitude, and confidence of health workers.

Methods

Study design

A quasi-experimental before-after design with intervention and comparison sites was used. Two purposively-selected districts (Ugu and Tshwane District) in South Africa in each of two geographically disparate provinces, KwaZulu-Natal (KZN) and Gauteng (Figure 1), were included for their differing infant feeding historical contexts: KZN has a history of strong political will to support breastfeeding, whilst Gauteng has historically supported formula feeding amongst MLHIV. Both provinces experienced a policy changed when infant feeding guidelines were updated in 2017.

Sampling

In Ugu District all four sub-districts were selected; within Tshwane District two of the seven service delivery regions were randomly selected.

Twelve intervention and six comparison primary health care clinics were randomly sampled in Ugu District and Tshwane District (separately). Only clinics with above the median number of annual clinic visits for children under-5 years in the district were eligible for inclusion in the sampling frame. The comparison clinics served to capture any temporal changes in health worker knowledge, confidence and attitudes due to other interventions or training provided by the health system and hence a smaller sample was required than for the intervention sites for which we required more precise estimates of the intervention effect. A two-stage process was used to recruit participants. Firstly, research staff explained the study and participant inclusion and exclusion criteria to each facility manager during face-to-face on-site introductory meetings. Then the facility manager compiled a list of all eligible health workers involved in the care of pregnant women and children, including nurses, midwives, visiting doctors, lay counsellors, dieticians, nutritionists, facility managers and community health workers (CHWs). In the second stage, research staff approached eligible health workers and invited them to participate in the research. We aimed to recruit 8-10 health workers per clinic to have a manageable group size to participate in the intervention, and in the evaluation. The same staff were approached for the baseline and follow-up evaluation.

Sample size

The sample size was determined based on 80% power and alpha 0.05 to measure a 15-percentage points difference in health worker confidence in HIV and infant feeding counselling between the intervention and comparison clinics comparing baseline and follow-up. The expected effect was based on the researchers' experience and data from recent studies in South Africa with the baseline level of high confidence to counsel HIV-positive women on breastfeeding duration set at 45%.[12] It was assumed that the confidence score would remain unchanged in the comparison clinics, implying a two-sample test in the post-intervention period. Clinic-level analyses were used for the

For peer review only - http://bmjopen.bmj.com/site/about/guidelines.xhtml

sample size calculations, assuming a sampling ratio of 2:1 for the intervention clinics and a standard deviation of 15% in the mean score between clinics. Based on these assumptions, and adjusting for clustering, the sample size was determined to be 24 intervention clinics and 12 comparison clinics.

Description of the intervention

We designed a participatory intervention consisting of on-site mentoring through three workshops in each clinic, targeting all health workers who provide care for pregnant women, breastfeeding mothers and their infants. This was delivered by the same trained facilitator (nurse in Gauteng or nutritionist in KZN) in each intervention clinic. Each workshop lasted 1-2 hours over a 3-6-week period and had well-defined learning outcomes. The intervention has been described elsewhere (Horwood et.al, in press). In summary, our participatory intervention was guided by evidence that health workers' attitudes and practices are influenced by various factors, not just exposure to training and information.[13] We used Dee Fink's six part taxonomy as a guiding theory. This proposes that significant learning only occurs by developing foundational knowledge, applying skills, integrating ideas, developing new feelings/interests and values, and learning how to learn (encouraging the spirit of enquiry) (Figure 2).[14] Additionally, we applied the theory of planned behaviour to the intervention design (Figure 2).[15 16] This states that an individual's intention to perform a behaviour is influenced by the person's attitudes towards performing the behaviour, their beliefs about whether people who are important to them will approve of the behaviour (subjective norms), and their beliefs about how likely they are to be able to implement the behaviour successfully. According to this theory, if health workers are to provide infant feeding counselling and support in accordance with updated infant feeding guidelines to HIV-infected and uninfected mothers, they need to agree with the change, believe that their colleagues and other stakeholders will approve of the action, and believe in their ability to implement it successfully. The workshops were tailored to achieve these goals: Workshop 1 covered knowledge gaps reported by participants, controversial statements, and advantages of breastfeeding. Following workshop 1, a poster or cards with key messages were placed in a prominent place in the clinic. Workshop 2 comprised a progressive case study discussed in small groups. Workshop 3 involved one-on-one mentorship: each participant was observed providing infant feeding counselling or a case study was discussed if no mothers were available for counselling. The same facilitator conducted all three workshops at each clinic. In addition, a WhatsApp cell phone messaging group was established to support participants in intervention sites to facilitate sharing of concerns, tips for counselling and dealing with difficult situations. Key messages were posted on the group approximately weekly. Comparison sub-districts were exposed to routine supervision and training activities that take place at district level. The study team documented whether and how the June 2017 circular issued by the National Department of Health, informing health facilities about the change in the 2013 Infant and Young Child feeding policy, was disseminated to comparison clinics.

Patient and Public Involvement

Patients and the public were not involved in the design of this study, as the main population of interest were health workers; thus, the intervention targeted health workers who were consulted to assist with intervention design. These details are explained in our intervention paper (Horwood et.al.,) which is currently under review.

For peer review only - http://bmjopen.bmj.com/site/about/guidelines.xhtml

Data collection

The primary outcome measure for the study was confidence level of health workers to counsel on infant feeding, evaluated using a Likert-scale tool, developed after reviewing existing tools and literature. Secondary outcomes included health worker knowledge and attitude about breastfeeding counselling. A baseline assessment amongst all participating health workers in intervention and comparison sites was undertaken prior to the start of the intervention (August 2017). Health workers self-completed the assessment on study-provided electronic tablets at their workplaces. Questions covered basic demographic information, types of activities undertaken at work, knowledge, attitudes and confidence around counselling on infant feeding. A mean of 12 weeks after the baseline assessment, a follow-up assessment using the same tool was conducted amongst the same group of health workers. The tool had been piloted amongst a separate group of health workers to determine length, complexity of questions and level of understanding. Health workers who were not in the clinic on the day of the follow-up assessments were included in a special catch-up assessment. Questionnaire software had built in range and skip logic and was transferred automatically to a database held at the University of KwaZulu-Natal.

Data analysis

There were three outcomes in the study: a) 22 knowledge statements which were scored 1 if correctly answered and 0 otherwise (binary outcomes); b) 21 attitude questions whose responses were measured on a 5-point Likert scale - given as completely disagree (1); disagree (2); neutral (3); agree (4) and completely agree (5); positive attitudes received higher scores and in some items, the scores were inverted; and c) 19 statements on confidence item questions which were also measured on a Likert scale, scored as such: not at all confident (1), not confident (2), confident (3) and very confident (4). For both attitude and confidence domains, a participant outcome was measured by the sum of the responses to the respective items (we verified that there was not a missing response on the items). Thus, the ranges for the attitude and confidence scores were 5 to 105, and 4 to 75, respectively.

Statistical Analysis

Participants baseline and follow-up characteristics and outcomes between the intervention and control areas were compared using Chi-squared tests for categorical variables and for continuous measures, two-sample t tests were used after confirmation of normality in the data. To assess the effect of the proposed intervention, several analysis methods for comparing treatment effect in pre-post quasi-experimental designs were considered. These include using post-measures and change from pre-treatment to post-treatment as the response variables. Approaches that use change and post measurements as response variables, adjusting for pre-treatment measures are preferred, and often give similar results.[17] In this paper, we used linear regression on change and post-measurements, adjusting for the level of the corresponding baseline measurement and characteristics. The analyses were adjusted for the possible clustering effect at the site level, using a variance-correction method. Data can be obtained by e-mailing the corresponding author.

For peer review only - http://bmjopen.bmj.com/site/about/guidelines.xhtml

Ethics

Ethics approval was obtained from the South African Medical Research Council (EC028-9/2016), the University of KwaZulu-Natal (RECIP348/17) and the WHO Ethics Review Committee (ERC0002833). Permission for undertaking the study was obtained from Tshwane and KZN Districts. Informed consent was sought from all study participants and no personal identifying information was captured in the questionnaires, only a study identification number.

Results

Tshwane and Ugu Districts differed significantly in three characteristics: Tshwane had significantly more participants who had worked for less than 2 years (14.4% versus 6.2%, p=0.007), significantly more registered nurses, and fewer lay counsellors/CHWs (57% versus 26.2% and 7.3% versus 50%, respectively, p=0.02 for the difference in participant's clinic roles in Tshwane versus Ugu districts); however, data from the two sites were combined for the analysis as the sites did not differ in the main outcomes measured (knowledge, attitude and confidence) at baseline. At baseline and follow-up, 23 intervention clinics (one large clinic was sampled twice with two rounds of data collection) and 12 comparison clinics were visited; 289 and 131 health care provider interviews were conducted at baseline, respectively (Figure 3). Loss to follow-up between baseline and follow-up did not differ between intervention and comparison sites: 17 (13%) in comparison sites versus 36 (12.5%) in intervention sites.

All staff approached agreed to participate. There were no significant differences between intervention and comparison sites at baseline, regarding district of origin, median age of respondent, gender, cadre of health worker, and working duration (Table 1). The proportion of participants who had received previous training (through the routine health system) on specific topics was similar in intervention versus comparison sites, except for three topics which had better coverage in comparison sites (Supplementary Figure 1). These were: ever trained on how to assess and support ART adherence for HIV-infected women (78.6% in intervention sites versus 89.2% in comparison sites, p=0.01); ever trained about managing breastfeeding problems (76.5% in intervention sites and 86.2% in comparison sites, p=0.02); and received any information or training about the revised infant feeding policy (55.1% in intervention sites versus 67.4% in comparison sites p=0.02). At baseline, activities around breastfeeding counselling and management were similar between comparison and intervention sites in all respects except that comparison site participants reportedly spoke more frequently to HIV-infected pregnant women about feeding than intervention participants (67% versus 71.6% spoke more than 1-3 times per month, p=0.04, data not shown).

For peer review only - http://bmjopen.bmj.com/site/about/guidelines.xhtml

Table 1: Characteristics of the participants in the intervention and comparison groups at baseline

Characteristic	Intervention group (n=289) (N (%))	Comparison group (n=131) (N (%))	p-value
**District:**			
- Tshwane	152 (52.6)	56 (42.8)	0.06
- Ugu	137 (47.4)	75 (57.3)	
**Age categories:**			
- 23 to 35 years	56 (19.4)	38 (29.7)	
- 36 to 41 years	61 (21.2)	25 (19.5)	
- 42 to 46 years	53 (18.4)	26 (20.3)	0.11
- 47 to 54 years	64 (22.2)	18 (14.1)	
- Over 54 years	54 (18.8)	21 (16.4)	
**Gender**			
- Female	267 (92.7)	118 (91.5)	0.66
- Male	21 (7.3)	11 (8.5)	
**Cadre of health worker**			
- Community level worker	84 (29.5)	52 (40)	
- Trained health professional*	151 (53.0)	64 (49.2)	0.05
- Enrolled nurse	50 (17.4)	14 (10.8)	
**Work experience in yrs**			
- Less than 1 yr	4 (1.4)	3 (2.3)	
- 1 to <2 yrs	23 (8.0)	12 (9.3)	
- 2 to less than 5 yrs	36 (12.5)	18 (14.0)	0.2
- 5 to less than 10 yrs	71 (24.7)	43 (33.3)	
- 10 yrs or more	154 (53.5)	53 (41.1)	

Abbreviations:; *includes 68% nurses in the intervention arm and 58% nurses in comparison arm. This group also includes operation managers, dieticians, doctors and nutritionists.

In intervention sites, workshops were attended by 84-88% of participants interviewed at follow-up (Table 2).

For peer review only

Table 2: Attendance at workshops 1-3 measured at follow-up in intervention sites

	Attended workshop n	Attended catch-up n	Total attended n/N (%)
**Number of staff attending each workshop:**			
Group workshop 1	202	63	265/303 (87.5)
Group workshop 2	223	34	257/303 (84.8)
Workshop 3 (Clinical mentoring)	216	40	256/303 (84.5)
**Number of workshops attended:**			
	number	%	
No workshop	18	7.2	
1-2 workshops	10	4.0	
All 3 workshops	221	88.8	
**Total**	**249**	**100**	

Effect of the intervention on health worker knowledge

At baseline, knowledge about key infant feeding statements or facts was similar between intervention and comparison sites, except for knowledge about soft porridge (Table 3). Although at baseline, more than 90% of intervention and comparison site participants knew that a baby under 4 months should not be given soft porridge if hungry, significantly more intervention site participants knew this recommendation (Table 3). The percentage of participants at baseline correctly answering the more difficult questions (on bottle sterilisation, storing expressed breastmilk, feeding HIV exposed infants) was low (Table 3). At follow-up significantly more intervention site participants correctly answered knowledge questions, regarding the leading cause of death in children under 5, the risk of formula feeding, duration of breastfeeding for HIV-negative mothers and MLHIV, how to stop breastfeeding, complementary feeding, storing expressed breastmilk, feeding whilst at work, breastfeeding and viral suppression, mixed feeding in MLHIV, adherence to ART and breastfeeding, breastfeeding difficulties in MLHIV and managing MLHIV who are breastfeeding, than comparison site participants (Table 3). The significant differences between intervention and comparison sites regarding soft porridge were not present at follow-up. Although improvements were seen in knowledge related to the risks of mixed feeding for MLHIV, most health workers still provided incorrect responses at follow-up. At baseline, the mean knowledge score was 15.0 (68%) in comparison sites versus 15.2 (69%) in intervention sites, p=0.89 (Table 3). At follow-up the mean knowledge score was to 15.2 (69%) in comparison sites and 17.2 (78.2%) in intervention sites, p<0.001 (Table 3). The difference in difference in the mean knowledge scores at baseline and follow-up between intervention and control sites was significant (p=0.0000, data not shown). At follow-up, knowledge scores of participants who attended 3 workshops compared with knowledge scores of participants who attended less than 3 workshops was significantly better (p=0.0000).

For peer review only - http://bmjopen.bmj.com/site/about/guidelines.xhtml

Table 3: Knowledge of health care workers about breastfeeding in the intervention and comparison sites at baseline and follow-up

Knowledge statements	Number (%) with correct answers at BASELINE			Number (%) with correct answers at FOLLOW-UP		
	Intervention (n=289)	Comparison (n=128)	p-value *	Intervention (n=250)	Comparison (n=112)	p-value *
**General breastfeeding**						
Exclusive breastfeeding is the recommended infant feeding method for ALL infants aged 0-6 months in SA, regardless of mother's HIV status (True)	271 (93.8)	118 (90.1)	0.18	234 (93.6)	102 (91.1)	0.39
Giving any formula milk during the first six months of life increases the risk of death from diarrhoea and/or pneumonia (True)	246 (85.1)	104 (79.4)	0.14	232 (92.8)	95 (84.8)	0.02
A mother who is working and giving formula milk should mix the milk herself and leave for the carer to give during the day (False)	218 (75.4)	94 (71.8)	0.42	189 (75.6)	68 (60.7)	<0.01
When sterilising feeding bottles cover the bottles with water in a saucepan and place on the heat. As soon as the water boils remove from heat and do not leave the bottles in the water until completely cool (False)	64 (22.2)	27 (20.6)	0.72	53 (21.2)	25 (22.3)	0.81
In South Africa, the leading cause of death amongst children under 5 is pneumonia (True)	189 (65.4)	82 (62.6)	0.58	230 (92.0)	75 (67.0)	<0.01
Continued breastfeeding for 2 years is the recommended infant method in SA for ALL children, regardless of mother's HIV status (True)	190 (65.7)	91 (69.5)	0.45	224 (89.6)	88 (78.6)	<0.01
A baby under 4 months should be given soft porridge once he/she seems hungry (False)	284 (98.3)	124 (94.7)	0.04	247 (98.8)	108 (96.4)	0.13
Giving a baby expressed breastmilk is not as good as breastfeeding (False)	234 (81.0)	106 (80.9)	0.99	218 (87.2)	96 (85.7)	0.70
It is safe to give the baby expressed breastmilk that has been kept outside the fridge for 8 hours (True)	106 (36.7)	43 (32.8)	0.44	120 (48.0)	38 (33.9)	<0.05
There are long term health benefits of breastfeeding for mother and child that last beyond the breastfeeding period (True)	264 (91.4)	116 (88.6)	0.37	232 (92.8)	100 (89.3)	0.26
**Breastfeeding and HIV**						
Mothers living with HIV who are receiving antiretroviral treatment and are virally suppressed should be advised not to breastfeed their infants (False)	252 (87.2)	115 (87.8)	0.87	230 (92.0)	99 (88.4)	0.27
When an HIV-infected mother is ready to add complementary feeds she should stop breastfeeding rapidly over a 24-hour period (False)	214 (74.1)	103 (78.6)	0.31	217 (86.8)	86 (76.8)	<0.05

For peer review only - http://bmjopen.bmj.com/site/about/guidelines.xhtml

If an HIV exposed baby is receiving both breastmilk and formula milk, the mother should choose either breastfeeding or formula feeding if she is adherent to ART (False)	69 (23.9)	29 (22.1)	0.70	75 (30.0)	28 (25.0)	0.33
An HIV-positive mother who is virally suppressed on antiretroviral treatment should breastfeed her child rather than not breastfeed to improve the child's survival (True)	237 (82.0)	108 (82.4)	0.91	236 (94.4)	96 (85.7)	<0.01
In South Africa, HIV-infected women who are breastfeeding should be supported to adhere to antiretroviral treatment and should be counselled and supported to exclusively breastfeed their infants for the first six months of life whilst maintaining an undetectable viral load (True)	281 (97.2)	123 (93.9)	0.10	242 (96.8)	109 (97.3)	0.79
A mother living with HIV and adherent to antiretroviral treatment cannot exclusively breastfeed her 4-month old infant because she is working. It is better for this mother to give formula during the day and breastfeed at night rather than giving no breast milk at all (True)	22 (7.6)	14 (10.7)	0.30	40 (16.0)	14 (12.5)	0.38
╞If a mother misses 2 doses of her ART in one month, she should be classified as a treatment failure (False)	185 (64.0)	82 (62.6)	0.78	191 (76.4)	73 (65.2)	<0.05
An HIV-positive mother who has cracked nipples should continue to breastfeed unless they are bleeding (True)	143 (49.5)	64 (48.9)	0.91	187 (74.8)	59 (52.7)	<0.01
╞An HIV-exposed baby who is exclusively breastfeeding should be given some water when the weather is very hot (False)	270 (93.4)	122 (93.13)	0.91	239 (95.6)	105 (93.8)	0.45
╞If a baby has a positive PCR (HIV test) at birth the mother should stop breastfeeding if this is affordable and feasible in her situation (False)	224 (81.0)	106 (80.9)	0.99	214 (85.6)	82 (73.2)	<0.01
╞A mother who has missed 6 tablets of FDC in one month is considered to be poorly adherent and should stop breastfeeding immediately (False)	181 (62.6)	89 (67.9)	0.29	201 (80.4)	72 (64.3)	<0.01
In South Africa, HIV-infected women who are breastfeeding should be supported to adhere to antiretroviral treatment and should introduce complementary foods around 6 months and be supported to continue breastfeeding for at least two years. (True)	245 (84.8)	116 (88.6)	0.30	244 (97.6)	91 (81.3)	<0.01
**Mean knowledge score (standard deviation) out of 22**	15.2 (2.6)	15.0 (3.1)	0.89*	17.2 (2.1)	15.2 (2.8)	<0.001

╞ The Statement is false; thus, the scales were inverted during data analysis.
*Mann-Whitney U test comparing intervention and comparison sites at the relevant time point.
Note: the tables displays numbers with correct knowledge

For peer review only

Effect of the intervention on attitudes

Comparing baseline and follow-up there were few significant differences between intervention and comparison sites in individual attitude statements towards breastfeeding (Supplementary Table 1). However, at follow-up, the mean attitude score towards breastfeeding was significantly higher (better) in intervention sites (p<0.001), (Supplementary Table 1). All three approaches to analysis demonstrated that, after controlling for other variables, final attitude (measured as attitude at follow-up, change in attitude between intervention and comparison sites or change in attitude between baseline and follow-up between intervention and comparison sites) was significantly better in intervention compared with comparison sites (Table 4). In these analyses, attitude at follow-up and change in attitude at follow-up was 5.36 points higher in the intervention group than the comparison group; the difference in attitudes using a diff-in-diff analysis showed a significant 4.40-point higher score in the intervention compared with the comparison group (Table 4). The first two analytical approaches demonstrated that being an enrolled nurse, and being in the youngest (36-41 years) or oldest (>54 years) age group was associated with a significantly lower attitude score; the diff-in-diff analysis demonstrated that, controlling for other factors, trained health professionals had a significantly higher attitude score at follow-up (Table 4). We did not detect a dose-association in intervention sites when comparing 0-1 or 1-2 versus 3 workshops (p=0.4); but numbers in each group may have been too small to reliably assess any dose effect.

Table 4: Adjusted effect of the intervention on health worker attitude score using different methods (Effect estimate and 95% confidence interval (CI))

**Variable**	**Modelling attitude score at follow-up**		**Modelling diff-in-diff attitude score**	
	**Effect estimate**	**95% CI**	**Effect estimate**	**95% CI**
**Attitude score at baseline**	**0.52**	**0.35; 0.69***	N/A	N/A
**Intervention**	**5.36**	**3.95; 6.76***	4.40	2.56; 6.23*
**Follow-up Period**	N/A	N/A	1.35	-0.15; 2.84
**Professional role: vs community level**				
- Trained health professional	1.62	-0.04; 3.28	**5.24**	**3.37; 7.11***
- Enrolled nurse	**-2.56**	**-5.10; -0.01***	-0.06	-2.33; 2.21
**Ugu District vs Tshwane District**	-0.82	-2.23; 0.59	-1.09	-3.00; 0.83
**Age category: vs 23-35 yrs**				
- 36 to 41 yrs	**-2.78**	**-5.29; -0.26***	-1.40	-3.86; 1.05
- 42 to 46 yrs	-0.91	-3.32; 1.50	-0.06	-2.45; 2.34
- 47 to 54 yrs	0.48	-1.96; 2.91	-0.89	-2.84; 1.05
- over 54 yrs	**-3.30**	**-5.57; -1.02***	-1.95	-4.32; 0.42
**Work experience <5 yrs vs ≥5yrs**	-0.36	-2.49; 1.77	-1.63	-3.88; 0.63

*p<0.005 N/A: not applicable. NB: All analyses are adjusted for clustering

For peer review only - http://bmjopen.bmj.com/site/about/guidelines.xhtml

Effect of the intervention on confidence

There was no difference in the percentage of participants in the intervention and control sites who were confident or very confident at baseline and at follow-up (Supplementary Table 2). However, the mean confidence score at follow-up was significantly higher in the intervention compared with the comparison sites at follow-up (p=0.05) (Table 5). All three approaches to analysis demonstrated that final confidence (measured as confidence at follow-up, change in confidence between intervention and comparison sites and change in confidence between baseline and follow-up between intervention and comparison sites) was significantly better in intervention compared with comparison sites (Table 5). Confidence at follow-up and change in confidence at follow-up was 2.42 points higher in the intervention arm than the comparison arm; the difference in confidence using a diff-in-diff analysis showed a significant 3.00-point higher score in the intervention compared with the comparison group (Table 5). Our analysis demonstrated that, controlling for other factors, being a trained health professional significantly increased confidence scores by 3.11 (ANCOVA or linear regression analysis) or 4.25 (diff-in-diff analysis). Additionally, the diff-in-diff analysis demonstrated that, controlling for other factors, working for less than 5 years significantly reduced the confidence score. We did not measure a dose-effect (one or two versus three workshops (p=0.4)); but numbers in each group may have been too small to assess this.

Table 5: Adjusted effect of the intervention on health worker confidence scores, using different multivariable analysis methods (Effect estimate and 95% confidence interval (CI))

Variable	Modelling confidence score at follow-up		Modelling diff-in-diff confidence score	
	Effect estimate	95% CI	Effect estimate	95% CI
**Confidence score at baseline**	**0.42**	**0.28; 0.56***	N/A	N/A
**Intervention**	**2.42**	**0.39; 4.45***	3.00	0.56; 5.43*
**Follow-up time**	N/A	N/A	0.01	-2.03; 2.05
**Cadre of heath professional: vs community level**				
- Trained health professional	**3.11**	**0.34; 5.87***	**4.25**	**2.14; 6.36***
- Enrolled nurse	-0.85	-4.17; 2.48	-1.82	-3.84; 0.20
**Ugu District vs Tshwane District**	0.00	-2.08; 2.08	-0.48	-2.71; 1.75
**Age category vs 23-35 yrs**				
- 36 to 41 yrs	-1.03	-3.77; 1.70	0.46	-2.21; 3.12
- 42 to 46 yrs	0.25	-2.85; 3.35	0.45	-2.19; 3.09
- 47 to 54 yrs	1.42	-0.73; 3.56	-0.89	-3.30; 1.51
- over 54 yrs	-2.28	-5.53; 0.97	-0.38	-3.62; 2.86
**Work experience <5 yrs vs ≥5 yrs**	-0.44	-3.31; 2.43	**-2.24**	**-4.06; -0.41***

*p<0.005 N/A= not applicable yrs= years. NB: All analyses are adjusted for clustering

For peer review only - http://bmjopen.bmj.com/site/about/guidelines.xhtml

Discussion

We implemented a participatory, team-based mentoring approach to disseminating updated HIV and infant feeding guidelines that had four distinct features: 1) it was on-site so that learning occurred in context and all cadres of health workers could attend (as lower cadres are frequently left out in off-site training); 2) it was team-based; all participants learned together; 3) content was led by the gaps in knowledge identified by participants themselves, and 4) activities were piloted and rooted in a theoretical framework. This mentorship approach was significantly associated with increased knowledge, especially around HIV and infant feeding, better attitudes and more confidence compared with a standard approach to disseminating infant feeding guidelines. Although some knowledge items did not change, we noted key improvements in the important knowledge items relating to HIV and infant feeding, however further reinforcement is needed regarding the issue of mixed feeding for MLHIV which is possibly the biggest practice change in the 2016 guidelines. Although some individual attitude and confidence items did not change at all, or only changed marginally, all our analyses indicated an improvement in follow-up attitude and confidence, controlling for clustering. Although the intervention group still performed poorly on some of the more difficult questions, we hypothesise that this could be attributed to the short duration of the intervention – three one-hour workshops over a period of three to six weeks. Notwithstanding this, we demonstrated shifts in key HIV-related parameters, and hypothesise that the overall change in knowledge, attitudes and confidence could positively influence infant feeding counselling and infant feeding practices.

There is ample evidence that in-service training and supervision improves the knowledge, skills and practices of health workers managing childhood undernutrition, and can improve health worker job satisfaction and motivation.[18 19] Post-training supervision and follow-up are included as key components of important child health programmes, such as the Integrated management of childhood illness strategy (IMCI)[20]; however, implementation challenges have been described, including inadequately trained or shortages of supervisors or inadequate job aids for follow-up, and if community views/ practise are not aligned to the programme then implementation and uptake is suboptimal.[21] Our approach provides a low technology, sustainable model for skills development at clinic level that could be used by existing personnel including supervisors and adapted to different settings and other areas of care quality or to dissemination of guidelines and improve confidence.

We used a mentorship approach to dissemination because mentorship is a holistic process that aims to empower the participant.[22] Using a team-based approach allows different cadres of health workers to learn together and could improve belief among health workers that their peers are supportive of the change in practice. Our findings demonstrate the advantages of a participatory, mentorship approach: In accordance with Dee Fink's theory, such an approach allows participants to develop foundational knowledge, apply skills, integrate ideas, develop new feelings/ interests and values and learn how to learn.[13] Our experience suggests that such an approach allowed discussion of the participant's attitudes towards performing the behaviour, their beliefs about whether people who are important to them will approve of the behaviour (subjective norms), and their beliefs about how likely they are to be able to implement the behaviour successfully.[15 16] Our findings corroborate a scoping review which demonstrated that

For peer review only - http://bmjopen.bmj.com/site/about/guidelines.xhtml

mentorship improves certain quality of care outcomes [22]; in our case it improved knowledge, attitudes and confidence. However, only four studies were included in this scoping review, and the nature of the mentorship varied from video-conferencing to monthly, six-weekly or annual visits interspersed with other contact forums, conducted over 1 day to an entire week. A list of desirable features of mentorship interventions, using insights gained from the scoping review, include at least one dedicated mentor per facility, ensuring an adequate mentor:mentee ratio so that all staff can be supported, forming meaningful relationships between mentors and mentees, ensuring cultural congruency between mentee and mentor, and using mentors for protocol-driven programmes, such as IMCI or HIV.[22] Our intervention related to HIV and infant feeding guidelines, was low cost and low technology (one mentor working with pen, flip chart and paper in the health facility), and was implemented by a dedicated mentor from the same cultural background as the mentees. She provided onsite support during the workshops, which lasted approximately one hour, and additional support through a WhatsApp messaging group.

Given the ongoing heath worker crisis in resource limited settings, including maldistribution of staff, an imbalance in skills mix, increasingly complicated health programmes and complicated socio-cultural-political-economic environments, some may question whether an on-site mentorship approach to guidelines dissemination is feasible to include within routine systems and services. We argue that strengthening investment in on-site mentorship rather than off-site training, improves health care management and likely to be a worthwhile and cost-effective health system investment (though not measured in this study). In our setting, all clinics receive regular visits from district primary health care (PHC) supervisors, who often focus on administrative aspects of clinic management. These supervisors, as well as existing district PHC trainers, could be capacitated to provide clinical mentoring for health workers in the clinics they oversee. In this way our model of team-based learning and mentoring is an example of how mentorship can be used for any clinical guideline update within the health system.

Our study had several limitations: We purposively selected districts for inclusion; we could not control for previous breastfeeding experience as we did not gather these data; the follow-up evaluation was done 3 months after the intervention; thus, we were only able to measure short term benefits and we did not measure the effect of improved knowledge, attitudes and confidence on actual infant feeding practices. The finding that knowledge scores at follow-up of participants who attended 3 workshops compared with knowledge scores at follow-up of participants who attended less than 3 workshops was significantly better may simply reflect better motivation amongst attendees of more workshops, rather than the effect of the workshops themselves. We could not tease out these effects, However, we were able to conduct several types of analyses and they all yielded congruent results.

Conclusion: We demonstrated improved knowledge, attitudes and confidence of health workers following a participatory mentorship approach to HIV and infant feeding guideline dissemination compared with a standard approach. More research is needed to better understand how to bring about changes in actual practice which may then improve breastfeeding practices.

For peer review only - http://bmjopen.bmj.com/site/about/guidelines.xhtml

What is already known?

Policies and clinical practice guidelines are updated regularly to ensure that health care is guided by the most recent evidence. In the area of HIV and infant feeding, guidelines have been updated frequently as evidence emerged, but effective dissemination of these guidelines has lagged behind. Multi-component dissemination strategies, which aim to increase the reach, ability and motivation of health workers, are more effective than one strategy alone. However, the standard of evidence to guide dissemination strategies in low-middle income settings where HIV prevalence is high, is low as studies are few and most studies have been conducted in high income countries. Thus, results may not apply to low-middle income countries, which have unique needs and challenges.

What are the new findings?

In primary health care clinics within South Africa (a high HIV prevalence setting), a quasi-experimental controlled before-after design comparing three 1-2 hour, on-site mentoring workshops over 3-6 weeks to disseminate updated guidelines on HIV and infant feeding with standard dissemination, showed positive results: At follow-up, mean knowledge, attitude and confidence scores of health workers (HW) participating in mentoring workshops were significantly higher than HW receiving standard dissemination ($p<0.001$, $p<0.01$ and $p=0.05$ respectively). Knowledge scores among HW who attended 3 workshops were significantly better (p=<0.001), compared with HW who attended <3 workshops.

What do the new findings imply?

In low-middle income settings, a low-technology, on-site mentoring approach to guideline dissemination significantly benefits HW knowledge attitudes and confidence, compared with dissemination through circulars. Low-middle income settings that have existing cadres of HW in supervisory roles should recommend that these cadres adopt a mentoring approach. Furthermore, they should test the effectiveness of mentoring-based supervision on HW knowledge, attitude, confidence, job satisfaction and morale when implemented at scale.

For peer review only - http://bmjopen.bmj.com/site/about/guidelines.xhtml

Authors contributions:

AG: Study conceptualization and tool development, protocol writing including intervention development, oversight of sampling and field work, writing of the first draft of this manuscript, receiving and incorporating co-author comments, finalization of the paper
TD: Study conceptualization and tool development, protocol writing including intervention development, set up the sample frame and sampling, contributed to the manuscript, reviewed and approved the final version of the manuscript
SM: Led the statistical components of the protocol; provided overall oversight on the statistical analysis, contributed to the manuscript, reviewed and approved the final version of the manuscript
TN: Performed the work on the statistical components of the protocol, under SM's guidance; provided data analysis under SM's guidance, contributed to the manuscript, reviewed and approved the final version of the manuscript
LH: Contributed to study conceptualization and tool development, protocol writing including intervention development; was overall Project Manager; established, managed and cleaned the database; contributed to the manuscript, reviewed and approved the final version of the manuscript
VJ: Provided guidance on intervention development. Contributed to the manuscript, reviewed and approved the final version of the manuscript
IMSE: Contributed to study conceptualization and tool development, protocol writing including intervention development; contributed to the manuscript, reviewed and approved the final version of the manuscript
UF: Contributed to study conceptualization, assisted with district level buy-in in Tshwane District, provided information on how routine dissemination of updated infant feeding guidelines; contributed to the manuscript, reviewed and approved the final version of the manuscript
MAD: Contributed to study conceptualization and tool development; contributed to the manuscript, reviewed and approved the final version of the manuscript
NR: Contributed to study conceptualization; contributed to the manuscript, reviewed and approved the final version of the manuscript
MK: Contributed to study conceptualization and tool development; contributed to the manuscript, reviewed and approved the final version of the manuscript
DS: Contributed to study conceptualization and tool development; contributed to the manuscript, reviewed and approved the final version of the manuscript
SK: Contributed to study conceptualization, assisted with national level buy-in, provided information on how routine dissemination of updated infant feeding guidelines; contributed to the manuscript, reviewed and approved the final version of the manuscript
TT: Contributed to study conceptualization and tool development; contributed to the manuscript, reviewed and approved the final version of the manuscript
CH: Study conceptualization, protocol writing including intervention development, high level oversight of study implementation, contributed to the manuscript, reviewed and approved the final version of the manuscript

Data sharing statement: Data can be obtained by e-mailing the corresponding author, and upon reasonable request

For peer review only - http://bmjopen.bmj.com/site/about/guidelines.xhtml

Figure legends:

Figure 1: Study districts: Tshwane District in Gauteng Province and Ugu District in KwaZulu-Natal Province of South Africa

Figure 2: Theoretical frameworks which informed the development of the intervention

Figure 3: Study population at baseline and follow-up for intervention and comparison sites

Supplementary Figure 1: Prior training reported by participants at baseline (intervention versus comparison groups)

For peer review only

For peer review only - http://bmjopen.bmj.com/site/about/guidelines.xhtml

References

1. McCormack L, Sheridan S, Lewis M, et al. Communication and dissemination strategies to facilitate the use of health-related evidence. Secondary Communication and dissemination strategies to facilitate the use of health-related evidence. 2013. https://www.ncbi.nlm.nih.gov/books/NBK179104/pdf/Bookshelf_NBK179104.pdf.
2. Victora C, Bahl R, Barros A, et al. Breastfeeding in the 21st century: epidemiology, mechanisms, and lifelong effect. The Lancet 2016;**387**(10017):475-90
3. Black RE, Allen LH, Bhutta ZA, et al. Maternal and child undernutrition: global and regional exposures and health consequences. Lancet. 2008.;**37**(9608):243-60
4. World Health Organisation. Antiretroviral drugs for treating pregnant women and preventing HIV infection in infants: towards universal access. Recommendations for a public health approach (2010 revision). In: Organisation WH, ed., 2010.
5. Six week extended dose nevirapine (SWEN) Study team et al. Extended dose nevirapine at 6 weeks of age for infants to prevent HIV transmission via breastfeeding in Ethiopia, India and Uganda: an analysis of 3 randomised controlled trials. Lancet 2008;**372**:300-13
6. Jamieson D, Chasela C, Hudgens M, et al. Maternal and infant antiretroviral regimens to prevent postnatal HIVtransmission: 48 weeks follow-up of the BAN randomised controlled trial. Lancet 2012;**379**:2449-258
7. World Health Organisation, UNICEF. 2016 Guideline Update on HIV and Infant Feeding. 2016 ed. Geneva, 2016.
8. World Health Organization, United Nations Children's Fund. Guideline: updates on HIV and infant feeding: the duration of breastfeeding, and support from health services to improve feeding practices among mothers living with HIV. . Geneva, 2016.
9. Academy of Nutrition and Dietetics, Handu D, Acosta A, et al. Effectiveness of interventions to promote exclusive breastfeeding in women living with HIV that are on antiretroviral therapy living in areas that promote exclusive breastfeeding due to limited resources for safe replacement feeding. , 2016.
10. Barker M, Baird J, Lawrence W, et al. The Southampton Initiative for Health: a complex intervention to improve the diets and increase the physical activity levels of women from disadvantaged communities. . J Health Psychol 2011;**16**:178-91
11. Baird J, Jarman M, Lawrence W, al. e. The effect of a behaviour change intervention on the diets and physical activity levels of women attending Sure Start Children's Centres: results from a complex public health intervention. . BMJ Open 2014;**4:e005290.**
12. Janse van Rensburg L, Nel R, Walsh CM. Knowledge, opinions and practices of healthcare workers related to infant feeding in the context of HIV. Health SA Gesondheid 2016;**21**:129-36 doi: http://dx.doi.org/10.1016/j.hsag.2015.12.001[published Online First: Epub Date]|.
13. GW. P, Gouws E, Bryce J, Burnham G, Team FtUIIS. Improving facility-based care for sick children in Uganda: training is not enough Health Policy Plan 2005;**20**(Suppl_1):i58-i68
14. Dee Fink L. A self-directed guide to designing courses for significant learning. Secondary A self-directed guide to designing courses for significant learning 2003. https://www.deefinkandassociates.com/GuidetoCourseDesignAug05.pdf.
15. Ajzen I. The theory of planned behavior. Organizational Behavior and Human Decision Processes 1991;**50**(2):179-211 doi: http://dx.doi.org/10.1016/0749-5978(91)90020-T[published Online First: Epub Date]|.

For peer review only - http://bmjopen.bmj.com/site/about/guidelines.xhtml

16. Ajzen I. Perceived Behavioral Control, Self-Efficacy, Locus of Control, and the Theory of Planned Behavior1. Journal of Applied Social Psychology 2002;**32**(4):665-83 doi: 10.1111/j.1559-1816.2002.tb00236.x[published Online First: Epub Date]|.
17. O'Connell N, Dai L, Jiang Y, Speiser J, Ward R, et al. Methods for Analysis of Pre-Post Data in Clinical Research: A Comparison of Five Common Methods. . J Biom Biostat 2017;**8**(334):doi:10.4172/2155-6180.1000334
18. Sunguya B, Poudel K, Mlunde L, Urassa D, Yasuoka J, Jimba M. Nutrition Training Improves Health Workers' Nutrition Knowledge and Competence to Manage Child Undernutrition: A Systematic Review. Frontiers in public health 2013:10.3389/fpubh.2013.00037
19. Bailey C, Blake C, Schriver M, Cubaka V, Thomas T, Martin Hilber A. A systematic review of supportive supervision as a strategy to improve primary healthcare services in Sub-Saharan Africa. Int J Gynaecol Obstet. 2016;**132**(1):117-25. doi: 10.1016/j.ijgo.2015.10.004.
20. World Health Organisation. Management of Childhood Illness in Developing Countries: Rationale for an integrated approach. 1999 ed, 1999.
21. Goga A, Muhe L. Global Challenges with Scale-Up of the Integrated Management of Childhood Illness Strategy: Results of a multi-country Survey. . BMC Public Health. 2011;**11**(503):doi: 10.1186/471-2458-11-503
22. Schwerdte P, Morphet, Hall H. A scoping review of mentorship of health personnel to improve the quality of health care i low and middle-income countries. Globalisation and Health 2017;**13**(77):doi 10.1186/s12992-017-0301-1

For peer review only

For peer review only - http://bmjopen.bmj.com/site/about/guidelines.xhtml

Figure 1: Study districts: Tshwane Metropolitan Municipality in Gauteng Province and Ugu District in KwaZulu-Natal Province of South Africa

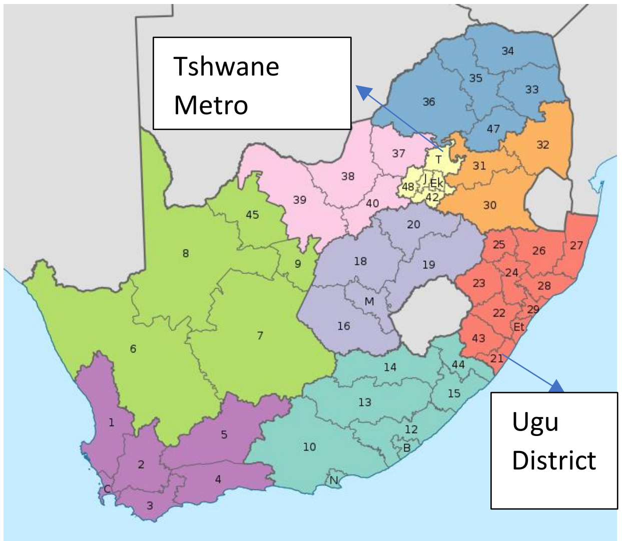

For peer review only - http://bmjopen.bmj.com/site/about/guidelines.xhtml

Figure 2: Theoretical frameworks which informed the development of the intervention

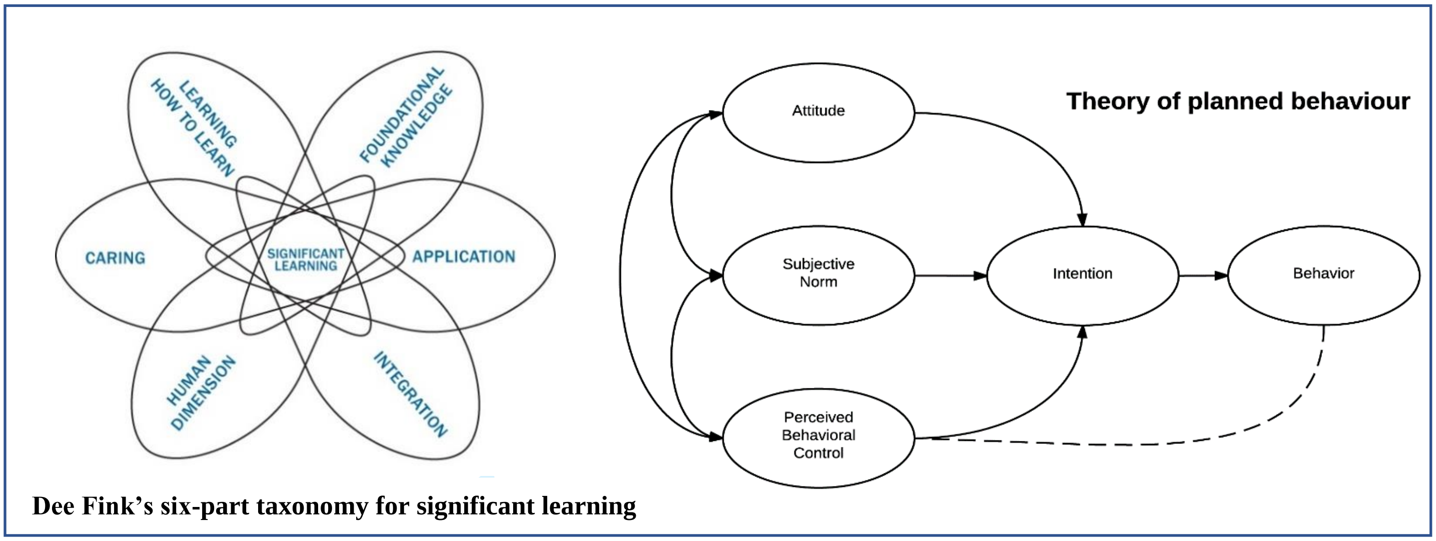

er review only

Figure 3: Study population at baseline and follow-up for intervention and comparison sites

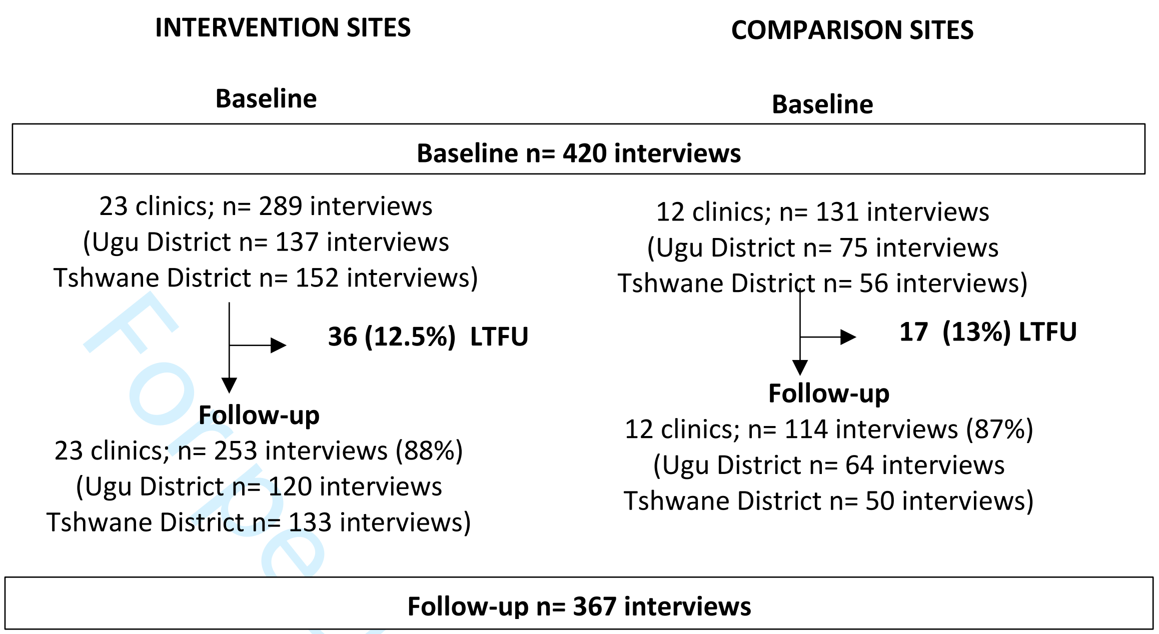

LTFU: lost to follow up

For peer review only

Supplementary Figure 1: Prior training reported by participants at baseline (intervention versus comparison groups)

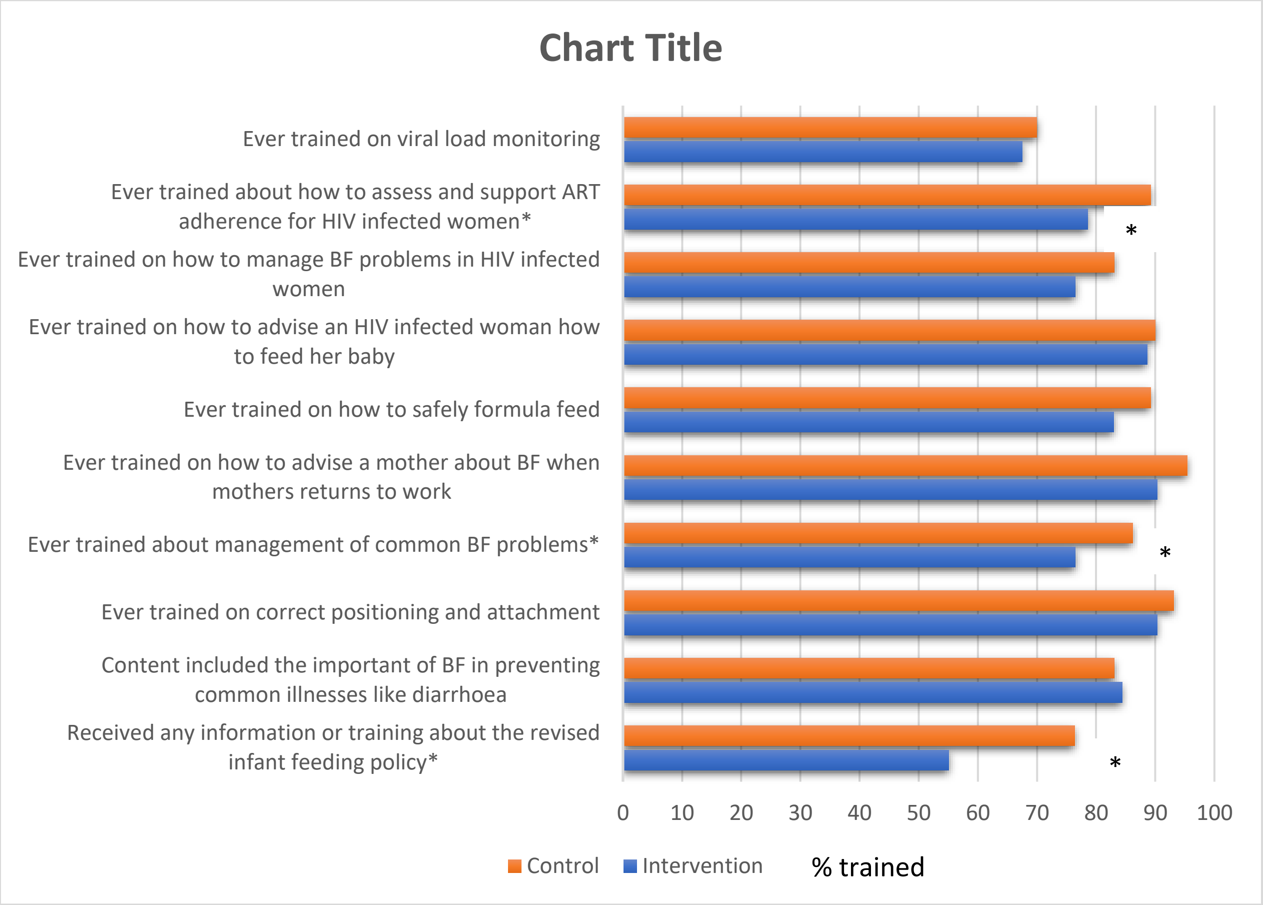

*p<0.05

Abbreviations: ART= antiretroviral therapy; BF= breastfeeding

For peer review only - http://bmjopen.bmj.com/site/about/guidelines.xhtml

Supplementary Table 1: Attitude scores of health workers at baseline and follow-up

Attitude statements	Number (%) who agreed or strongly agreed with the statement at baseline			Number (%) who agreed or strongly agreed with the statement at follow-up		
	Intervention (n=289)	Comparison (n=128)	p-value *	Intervention (n=250)	Comparison (n=112)	p-value*
There have been so many changes to the infant feeding guidelines and breastfeeding guidelines however I am NOT confused about what to tell mothers who are HIV-infected about breastfeeding	155 (53.6)	74 (56.9)	0.09	174 (69.6)	63 (56.3)	0.14
When a baby cries all the time it is NOT usually because the baby is hungry and needs more food than just breastmilk	260 (90.0)	107 (83.0)	0.14	229 (91.6)	90 (80.4)	0.18
Exclusive breastfeeding in the first 6 months of life is the best choice for all mothers and babies in South Africa	256 (88.6)	114 (88.4)	0.06	238 (95.2)	69 (85.7)	0.22
For an HIV-exposed infant any breastfeeding is better than no breastfeeding at all, as long as the mother is virally suppressed and on antiretroviral therapy	189 (65.4)	79 (61.2)	0.06	195 (78.0)	73 (65.2)	0.18
The benefits of breastfeeding for protecting children from illness such as diarrhoea and pneumonia outweighs the risk of acquiring HIV if the mother is on antiretroviral treatment	224 (77.5)	93 (72.1)	0.12	216 (86.4)	86 (76.8)	0.17
I feel that an HIV-infected mother who has not disclosed to her partner is NOT at high risk of non-adherence to ART and should NOT stop breastfeeding as soon as possible	128 (44.3)	51 (39.5)	0.09	136 (54.4)	44 (39.3)	0.17
I should support all mothers, regardless of HIV status, to continue breastfeeding until 2 years, as long as HIV-infected women are virally suppressed	137 (82.0)	101 (78.3)	0.08	236 (94.4)	86 (76.8)	0.30
I should NOT advise an HIV-positive virally suppressed mother who has cracked and bleeding nipples to temporarily stop breastfeeding	80 (27.7)	40 (31.3)	0.07	96 (38.4)	24 (21.4)	0.18
HIV-exposed babies who are PCR negative must NOT stop breastfeeding as soon as possible	238 (82.4)	104 (81.3)	0.11	220 (88.0)	93 (83.0)	0.14
Formula feeding is NOT the best choice for mothers living in good socio-economic circumstances who are going back to work	202 (70.0)	95 (74.2)	0.07	197 (78.8)	72 (64.3)	0.17
For an HIV-positive mother on antiretroviral treatment and virally suppressed mixed feeding is better than not breastfeeding at all	39 (13.5)	15 (11.7)	0.08	57 (22.8)	15 (13.5)	0.12
Exclusive breastfeeding for six months is an achievable goal for the majority of mothers	249 (86.2)	105 (82.0)	0.10	225 (90.0)	94 (83.9)	0.13
It is safer for HIV-positive mothers to breastfeed than to formula feed	231 (79.9)	89 (69.5)	0.06	225 (90.0)	83 (74.1)	0.27

For peer review only

For peer review only - http://bmjopen.bmj.com/site/about/guidelines.xhtml

In our community working mothers can successfully maintain exclusive breast feeding while going to work	219 (75.8)	89 (69.5)	0.08	207 (82.8)	90 (80.4)	0.11
An HIV-positive mother who is on ART and not virally suppressed and is mixed feeding is putting her child at risk of acquiring HIV	256 (88.6)	109 (85.2)	0.11	219 (87.6)	95 (84.8)	0.07
It is NOT very difficult for mothers to express breastmilk while they are at work or school	164 (53.0)	57 (44.5)	0.12	168 (67.2)	50 (44.7)	0.23
If an HIV-positive mother can afford to buy formula it is NOT better for her to formula feed her baby	170 (58.8)	83 (64.9)	0.06	184 (73.6)	66 (58.9)	0.19
Promoting breastfeeding for two years for HIV-exposed infants is NOT a risk because mothers will be able to maintain good ART adherence for that long	197 (68.2)	85 (66.4)	0.11	209 (83.6)	78 (69.6)	0.18
In South Africa it is possible to improve exclusive breastfeeding rates	244 (84.7)	108 (84.4)	0.08	229 (91.6)	95 (84.8)	0.15
There are exceptional circumstances where an HIV-positive mother would be advised not to breastfeed, such as failure of 2nd or 3rd line ART treatment, but these are not common	225 (77.9)	93 (72.7)	0.08	200 (80.0)	86 (76.8)	0.12
Formula feeding is NOT more convenient for a mother than breastfeeding	253 (87.5)	109 (85.2)	0.09	211 (84.4)	98 (87.5)	0.11
**Number (%) participants whose attitude was to at least agree (Attitude score ≥84)***	**71 (24.6)**	**23 (17.9)**	**0.12**	**123 (49.2)**	**27 (24.1)**	**<0.01**
**Mean attitude score out of 105 (95% CI)**	**76.9 (75.9 – 77.9)**	**75.0 (73.0 – 77.0)**	**0.07**	**82.7 (81.6 – 83.8)**	**76.8 (75.0 – 78.5)**	**<0.01**

*84 was the minimum score obtainable if a participant at least agreed with all statements

For peer review only - http://bmjopen.bmj.com/site/about/guidelines.xhtml

Supplementary Table 2: Confidence statements of health workers at baseline and follow-up

Confidence statements	Number (%) who felt confident or very confident at baseline			Number (%) who felt confident or very confident at follow-up		
	Intervention (n=289)	Comparison (n=128)	p-value	Intervention (n=250)	Comparison (n=112)	p-value
How confident do you feel about counselling an HIV-positive pregnant woman about how she will feed her baby	265 (91.7)	116 (90.6)	0.03	239 (95.6)	98 (87.5)	0.19
How confident do you feel about giving information about the risks and benefits of breastfeeding to an HIV-infected mother	268 (92.7)	120 (93.6)	0.07	236 (94.4)	100 (89.3)	0.11
How confident do you feel about assessing whether there is good positioning and attachment during breastfeeding	263 (91.0)	125 (97.7)	0.13	241 (96.8)	105 (93.8)	0.07
How confident do you feel about advising an HIV-positive mother about how to continue to breastfeed her baby when she returns to work or school	258 (89.3)	117 (91.4)	0.08	240 (96.0)	99 (88.4)	0.18
How confident do you feel about advising an HIV-infected mother who is virally suppressed who is mixed feeding her infant	243 (84.1)	113 (88.3)	0.06	224 (89.6)	97 (86.6)	0.05
How confident do you feel about advising an HIV-infected mother to continue breastfeeding for two years	216 (74.7)	105 (82.0)	0.09	234 (93.6)	91 (81.3)	0.21
How confident do you feel about advising an HIV-infected mother about how to stop breastfeeding	214 (74.1)	89 (69.5)	0.09	188 (75.2)	84 (75.0)	0.05
How confident do you feel about advising an HIV-positive mother about starting complementary feeds	251 (86.9)	115 (89.9)	0.05	227 (90.8)	101 (90.2)	0.12
How confident do you feel about assessing ART compliance in an HIV-positive mother	240 (83.1)	111 (86.7)	0.08	229 (91.6)	92 (82.1)	0.16
How confident do you feel about identifying when an HIV-positive mother is not adhering to her ART treatment	224 (77.5)	104 (81.3)	0.09	216 (86.4)	88 (78.6)	0.18
How confident do you feel about reassuring a mother living with HIV who is virally suppressed that a shorter duration of breastfeeding is better than never initiating breastfeeding	227 (78.5)	103 (80.5)	0.08	214 (85.6)	94 (83.9)	0.06
How confident do you feel about explaining the risks of HIV transmission through breastmilk to an HIV-infected mother with high viral load	247 (85.5)	114 (89.1)	0.06	218 (87.2)	103 (92.0)	0.09
How confident do you feel about assisting a mother with HIV to safely formula feed her baby	212 (73.4)	102 (79.7)	0.12	190 (76.0)	93 (83.0)	0.11

For peer review only - http://bmjopen.bmj.com/site/about/guidelines.xhtml

How confident do you feel about advising an HIV-infected mother who is exclusively breastfeeding and has cracked nipples with bloody milk about how to feed her baby	196 (67.8)	84 (65.6)	0.11	200 (80.0)	78 (69.6)	0.13
How confident do you feel about using the guidelines for safe replacement feeding when you counsel a mother who is not adherent to ART and has a viral load above 1000 copies/ml	191 (66.1)	99 (77.4)	0.17	184 (73.6)	74 (66.1)	0.13
How confident do you feel about advising an HIV-infected mother who is exclusively breastfeeding and has defaulted from her ART about how to feed her baby	205 (70.9)	97 (75.8)	0.11	187 (74.8)	79 (70.5)	0.08
How confident do you feel about explaining to a mother about expressing and storing milk	269 (93.1)	124 (96.9)	0.10	235 (94.0)	107 (95.5)	0.06
How confident do you feel about managing poor ART compliance in an HIV-infected breastfeeding mother	215 (74.4)	104 (81.3)	0.15	207 (82.8)	87 (77.7)	0.11
A mother is not adherent to ART and her last viral load is 1000 copies per ml. How confident do you feel about counselling her about feeding her infant?	199 (68.9)	98 (76.6)	0.10	196 (78.4)	78 (69.9)	0.12
**Number (%) participants who were confident or very confident (Sum Score ≥ 57)**	**164 (56.8)**	**86 (67.2)**	**0.09**	**175 (70.0)**	**72 (64.3)**	**0.28**
**Mean confidence score out of 76 (95% CI)**	**59.1 (58.0-60.2)**	**59.1 (57.0-61.3)**	**1.0**	**61.2 (60.8-63.1)**	**59.9 (58.1-61.7)**	**0.05**

For peer review only - http://bmjopen.bmj.com/site/about/guidelines.xhtml

BMJ Open

Translating new evidence into clinical practice: the effect of a novel outreach mentoring approach on the knowledge, attitudes and confidence of health workers providing HIV and infant feeding counselling in South Africa

Journal:	*BMJ Open*
Manuscript ID	bmjopen-2019-034770.R1
Article Type:	Original research
Date Submitted by the Author:	22-May-2020
Complete List of Authors:	Goga, Ameena; Medical Research Council of South Africa, Health Systems Research Unit and HIV Prevention Research Unit; University of Pretoria Faculty of Health Sciences, Paediatrics<br>Doherty, Tanya; South African Medical Research Council<br>Manda, Samuel; South African Medical Research Council<br>Nkwenika, Tshifhiwa; SAMRC<br>Haskins, Lyn; University of KwaZulu-Natal<br>John, Vaughn; University of KwaZulu-Natal, School of Education<br>Engebretsen, Ingunn; University of Bergen Faculty of Medicine and Dentistry, Centre for International Health<br>Feucht, Ute; University of Pretoria<br>Dhansay, Ali; Burden of Disease Research Unit<br>Rollins, Nigel; World Health Organization, Department of Maternal, Newborn, Child and Adolescent Health<br>Kroon, Max; University of Cape Town Faculty of Health Sciences, Neonatology<br>Sanders, David; University of the Western Cape, South Africa , School of Public Health<br>Kauchali, Shuaib; South African National Department of Health<br>Tylleskär, Thorkild; Universitetet i Bergen, Centre for International health<br>Horwood, Christiane ; University of KwaZulu-Natal,
**Primary Subject Heading**:	Global health
Secondary Subject Heading:	Public health
Keywords:	Public health < INFECTIOUS DISEASES, HIV & AIDS < INFECTIOUS DISEASES, Community child health < PAEDIATRICS

SCHOLARONE™
Manuscripts

I, the Submitting Author has the right to grant and does grant on behalf of all authors of the Work (as defined in the below author licence), an exclusive licence and/or a non-exclusive licence for contributions from authors who are: i) UK Crown employees; ii) where BMJ has agreed a CC-BY licence shall apply, and/or iii) in accordance with the terms applicable for US Federal Government officers or employees acting as part of their official duties; on a worldwide, perpetual, irrevocable, royalty-free basis to BMJ Publishing Group Ltd ("BMJ") its licensees and where the relevant Journal is co-owned by BMJ to the co-owners of the Journal, to publish the Work in this journal and any other BMJ products and to exploit all rights, as set out in our licence.

The Submitting Author accepts and understands that any supply made under these terms is made by BMJ to the Submitting Author unless you are acting as an employee on behalf of your employer or a postgraduate student of an affiliated institution which is paying any applicable article publishing charge ("APC") for Open Access articles. Where the Submitting Author wishes to make the Work available on an Open Access basis (and intends to pay the relevant APC), the terms of reuse of such Open Access shall be governed by a Creative Commons licence – details of these licences and which Creative Commons licence will apply to this Work are set out in our licence referred to above.

Other than as permitted in any relevant BMJ Author's Self Archiving Policies, I confirm this Work has not been accepted for publication elsewhere, is not being considered for publication elsewhere and does not duplicate material already published. I confirm all authors consent to publication of this Work and authorise the granting of this licence.

For peer review only

Translating new evidence into clinical practice: the effect of a novel outreach mentoring approach on the knowledge, attitudes and confidence of health workers providing HIV and infant feeding counselling in South Africa

Ameena Goga[1, 2, 3], Tanya Doherty[1,4,5], Samuel Manda[6, 7], Tshifhiwa Nkwenika[6], Lyn Haskins[8], Vaughn John[9], Ingunn M.S. Engebretsen[10], Ute Feucht[2, 11,12, 13], Ali Dhansay[14, 15] Nigel Rollins[16], Max Kroon[17, 18], David Sanders[4,19], Shuaib Kauchali[20], Thorkild Tylleskar[10,] Christiane Horwood[8]

Affiliations

[1]Health Systems Research Unit, South African Medical Research Council, South Africa
[2]Department of Paediatrics, University of Pretoria, South Africa
[3]HIV Prevention Research Unit, South African Medical Research Council, South Africa
[4]School of Public Health, University of the Western Cape, South Africa
[5]School of Public Health, University of the Witwatersrand
[6]Biostatistics Unit, South African Medical Research Council, South Africa
[7]Department of Statistics, University of Pretoria, South Africa
[8]Centre for Rural Health, University of KwaZulu-Natal, South Africa
[9]School of Education, University of KwaZulu Natal, Pietermaritzburg, South Africa
[10]Centre for International Health, University of Bergen, Norway
[11]Tshwane District Health Services, Gauteng Department of Health, city? South Africa
[12]Research Centre for Maternal, Fetal, Newborn and Child Health Care Strategies, University of Pretoria, Pretoria, South Africa
[13]Maternal and Infant Health Care Strategies Research Unit, South African Medical Research Council, South Africa
[14]Division of Human Nutrition and Department of Paediatrics and Child Health, Stellenbosch University, South Africa.
[15]Burden of Disease Research Unit, South African Medical Research Council, South Africa
[16]Department of Maternal, Newborn, Child and Adolescent Health, World Health Organization, Geneva, Switzerland
[17]Department of Neonatology, Faculty of Health Sciences, University of Cape Town
[18]Mowbray Maternity Hospital, Cape Town, South Africa
[19]Department of Paediatrics and Child Health, University of Cape Town, South Africa
[20]National Department of Health, South Africa

Corresponding author: Ameena.Goga@mrc.ac.za

For peer review only - http://bmjopen.bmj.com/site/about/guidelines.xhtml

Abstract

Objectives: We report the effectiveness of a facility-based mentoring approach to improve health workers' knowledge, attitudes and confidence with counselling on HIV and infant feeding.
Design: A quasi-experimental controlled before-after study.
Setting: Primary health care clinics were randomly selected (n=24 intervention, n=12 comparison) from two districts, South Africa.
Participants: Health workers (HW) providing infant feeding counselling.
Intervention: three 1-2 hour, on-site workshops over 3-6 weeks.
Primary outcome measures: a) knowledge scores: 22 statements each scored 1 if correct or 0; b) attitude scores: 21 statements with 5 possible responses: completely disagree (1), disagree (2), neutral (3), agree (4) and completely agree (5); c) confidence scores: 19 statements with 4 possible responses: not at all confident (1), not confident (2), confident (3) and very confident (4). Analysis was at the HW level, with clinic as a random effect to adjust for clustering. Data were analyzed using STATA/SE version 15.
Results: In intervention and comparison sites, respectively: 289 and 131 baseline and 253 and 114 follow-up interviews were conducted (August-December 2017). At follow-up, the mean score for the 22 knowledge questions was 15.2 (69%) in comparison and 17.2 (78.2%) in intervention sites (p<0.001). Knowledge scores among HW who attended 3 (versus <3) workshops was significantly better (p<0.0001). At follow-up, the mean attitude and confidence scores were significantly better in intervention versus comparison sites (p<0.001 and p=0.05, respectively). Controlling for other factors there was a significant 5.1-point higher attitude score, and a non-significant 1.5 point increase in confidence score in the intervention group compared with the comparison group.
Conclusion: A participatory, low intensity on-site mentoring approach to disseminating updated infant feeding guidelines improved HWs' knowledge, attitudes and confidence more than standard dissemination. Further research is required to evaluate the effectiveness, feasibility and sustainability of implementing such a mentoring approach at scale.

Strengths and limitations of the study:

1. Fieldwork was conducted in two geographically and historically different provinces, facilitating generalisability of results.
2. The intervention was participatory, low intensity, on-site and integrated into routine facilities.
3. Several types of analyses were conducted which all yielded congruent results.
4. However, limitations were that (i) we purposively selected districts for inclusion (ii) we could not control for HWs' personal breastfeeding experience as we did not gather these data (iii) the follow-up evaluation was undertaken 3 months after the intervention - thus, we measured short term benefits, and did not measure the direct effect of improved HWs' knowledge, attitudes and confidence on mothers' infant feeding practices.
5. The finding that knowledge scores amongst participants who attended 3 workshops were significantly better than knowledge scores amongst participants who attended less than 3 workshops, may simply reflect better motivation amongst attendees of more workshops, rather than the effect of the workshops themselves. We could not tease out these effects.

Funding statement

For peer review only - http://bmjopen.bmj.com/site/about/guidelines.xhtml

This study was funded by the World Health Organization (Ref: 2017/712509-0). Tanya Doherty receives a small grant from the National Research Foundation, South Africa. Ameena Goga and Tanya Doherty's time was funded by the South African Medical Research Council. The publication of paper was funded by the South African Medical Research Council.

Competing interests statement

No author has declared any competing interests.

For peer review only

Introduction

Policies and clinical practice guidelines are updated regularly following the accumulation of recent evidence. In the area of HIV and infant feeding, strategies to facilitate the effective dissemination of these updated guidelines have lagged behind. Research demonstrates that ,multi-component dissemination strategies, which aim to increase the reach, ability and motivation of health workers, are more effective than one strategy alone.[1] However, the reality is that there are few published studies to inform strategies for guideline dissemination, and most of these studies have been conducted in high income countries. Their results may therefore not be relevant to low-middle income settings, which have unique challenges.[1] We studied the effectiveness of a mentorship approach to disseminate updated HIV and infant feeding guidelines amongst health workers in a middle income setting with high HIV prevalence.

The benefits of breastfeeding in all settings, and particularly in low-middle income settings with high HIV prevalence, are undisputed.[2 3] In 2010, the World Health Organization (WHO) issued HIV and infant feeding recommendations, in support of six to 12 months of breastfeeding under antiretroviral cover.[4] This followed a difficult period during which effective triple antiretroviral therapy (ART) was not available and breastfeeding avoidance was recommended if specific conditions conducive to the safe use of replacement feeds were met. In 2009, data emerged that maternal ART and infant antiretroviral prophylaxis greatly reduce breast milk HIV transmission risk.[5 6] In 2011 the Tshwane Declaration of support for breastfeeding was adopted in South Africa and the provision of free commercial infant formula to prevent vertical HIV transmission (MTCT) was phased out.[7] These recommendations were followed by the implementation of PMTCT Option B+ in 2015, including lifelong ART for all pregnant and lactating women living with HIV, and support for continued breastfeeding for one year.[8] In July 2016, the WHO guidelines on HIV and infant feeding were updated to recommend exclusive breastfeeding (EBF) for the first six months, appropriate complementary foods from about 6 month and continued breastfeeding for at least 12 to 24 months amongst HIV-negative mothers and mothers living with HIV who were fully supported for ART adherence.[9] In June 2017, these feeding recommendations were adopted in South Africa, and revisions were communicated through a circular issued by the National Department of Health, necessitating health worker re-training.

For peer review only - http://bmjopen.bmj.com/site/about/guidelines.xhtml

Research has demonstrated that improving HWs' capacity can significantly improve their skills, self-efficacy and confidence to counsel, support and promote breastfeeding among mothers living with HIV.[10] .[11] Consequently, a key question was: What learning approach could best develop health worker capacity and confidence to implement the updated HIV and infant feeding guideline, using a methodology that is sustainable and feasible to implement at scale. Pedagogical research highlights the advantage of participatory training compared with standard didactic teaching for improving health worker skills.[12 13] Thus, we sought to determine whether a participatory outreach mentorship approach to disseminate the updated HIV and infant feeding guidelines, using simple low-technology activities, improves health workers' knowledge of, attitudes towards and confidence with counselling on HIV and infant feeding..

Methods

Study design

A quasi-experimental before-after design with intervention and comparison sites was used. Two purposively-selected districts (Ugu and Tshwane District) in South Africa in each of two geographically disparate provinces, KwaZulu-Natal (KZN) and Gauteng (Figure 1), were included for their differing infant feeding historical contexts: KZN has a history of strong political will to support breastfeeding, whilst Gauteng has historically supported formula feeding amongst mothers living with HIV. Both provinces experienced a policy change when infant feeding guidelines were updated in 2017.

Sampling

In Ugu District all four sub-districts were selected; within Tshwane District two of the seven service delivery regions were randomly selected.

Twelve intervention and six comparison primary health care clinics were randomly sampled in Ugu District and Tshwane District (separately). Only clinics with above the median number of annual clinic visits for children under-5 years in the district were eligible for inclusion in the sampling frame. The comparison clinics served to capture any temporal changes in health worker knowledge, confidence and attitudes due to other interventions or trainings; hence a smaller sample was required in comparison versus intervention sites as the latter required more precise estimates of the intervention effect. A two-stage process was used to recruit participants. Firstly, research staff explained the study and participant inclusion and exclusion criteria to each facility manager during face-to-face on-site introductory meetings. The facility manager compiled a list of all eligible health workers involved in the care of pregnant women and children, including nurses, midwives, visiting doctors, lay counsellors, dieticians, nutritionists, facility managers and community health workers (CHWs). In the second stage, research staff approached eligible health workers and invited them to participate in the research. We aimed to recruit a manageable size of 8-10 health workers per clinic for participation in the intervention, and in the evaluation. The same staff were approached for the baseline and follow-up evaluations.

Sample size

The sample size was determined based on 80% power and alpha 0.05 to measure a 15-percentage points difference in health worker confidence in HIV and infant feeding counselling between the intervention and comparison clinics comparing baseline and follow-up. The expected effect was based on the researchers' experience and data from recent studies in South Africa with the baseline

For peer review only - http://bmjopen.bmj.com/site/about/guidelines.xhtml

level of high confidence to counsel HIV-positive women on breastfeeding duration set at 45%.[14] It was assumed that the confidence score would remain unchanged in the comparison clinics, implying a two-sample test in the post-intervention period. Clinic-level analyses were used for the sample size calculations, assuming a sampling ratio of 2:1 for the intervention clinics and a standard deviation of 15% in the mean score between clinics. Based on these assumptions, and adjusting for clustering, the sample size was determined to be 24 intervention clinics and 12 comparison clinics.[15]

Description of the intervention

We designed a participatory intervention comprising on-site mentoring through three workshops in each clinic, involving selected health workers who provide care for pregnant women, breastfeeding mothers and their infants. The intervention was delivered by the same trained facilitator (a nurse in Gauteng and nutritionist in KZN) in each intervention clinic. Each workshop lasted 1-2 hours over a 3-6-week period and had well-defined learning outcomes. The intervention has been described elsewhere .[16] In summary, our participatory intervention was guided by evidence that health workers' attitudes and practices are influenced by various factors, not just exposure to training and information.[17] We used Dee Fink's six part taxonomy as a guiding theory. This proposes that significant learning only occurs by developing foundational knowledge, applying skills, integrating ideas, developing new feelings/interests and values, and learning how to learn (encouraging the spirit of enquiry) (Figure 2).[18] Additionally, we applied the theory of planned behaviour to the intervention design (Figure 2).[19 20] This states that an individual's intention to perform a behaviour is influenced by the person's attitudes towards performing the behaviour, their beliefs about whether people who are important to them will approve of the behaviour (subjective norms), and their beliefs about how likely they are to be able to implement the behaviour successfully. According to this theory, if health workers are to provide infant feeding counselling and support in accordance with updated infant feeding guidelines to HIV-positive or negative mothers, they need to agree with the change, believe that their colleagues and other stakeholders will approve of the action, and believe in their ability to implement it successfully. The workshops were tailored to achieve these goals: Workshop 1 covered knowledge gaps reported by participants, controversial statements, and advantages of breastfeeding. Following workshop 1, a poster or cards with key messages were placed in a prominent place in the clinic. Workshop 2 comprised a progressive case study discussed in small groups. Workshop 3 involved one-to-one mentorship: each participant was observed providing infant feeding counselling or a case study was discussed if no mothers were available for counselling. The same facilitator conducted all three workshops at each clinic. In addition, a WhatsApp cell phone messaging group was established to support participants in intervention sites to facilitate sharing of concerns, tips for counselling and dealing with difficult situations. Key messages were posted on the group approximately weekly. Comparison sub-districts were exposed to routine supervision and training activities that took place at district level. The study team documented that the June 2017 circular issued by the National Department of Health, informing health facilities of the change in Infant and Young Child feeding policy, was disseminated to comparison clinics as an announcement via e-mail and other electronic communication as well as during meetings or trainings. We documented that in Tshwane, 15 of the 18 clinics had received the circular; 11 via

For peer review only - http://bmjopen.bmj.com/site/about/guidelines.xhtml

e-mail and three at a meeting. In Ugu nine of 17 clinics had received the circular; 8 received it via hand delivery and one via e-mail.

.

Patient and Public Involvement

Patients and the public were not involved in the design of this study, as the main population of interest were health workers.. The intervention and tool were piloted amongst a separate group of health workers to determine length, complexity of questions and level of understanding. These details are explained in our intervention paper..[16]

Data collection

Data were collected between August and December 2017 by dedicated trained non-nurse data collectors who were independent of the intervention staff. As per study design, data collection staff were not part of any intervention activities and had never been exposed to the intervention. The primary outcome measure for the study was confidence level of health workers to counsel on infant feeding, evaluated using a Likert-scale tool, developed after reviewing the WHO Breastfeeding Counselling Course, and the WHO HIV and Infant feeding counselling courses.[9 21-24].Secondary outcomes included health worker knowledge and attitude about breastfeeding counselling. A baseline assessment amongst all participating health workers in intervention and comparison sites was undertaken prior to the start of the intervention (August 2017). Health workers self-completed the assessment on study-provided electronic tablets at their workplaces. Questions covered basic demographic information, types of activities undertaken at work, knowledge, attitudes and confidence around counselling on infant feeding. A mean of 12 weeks after the baseline assessment, a follow-up assessment using the same tool was conducted amongst the same group of health workers. Health workers who were not in the clinic on the day of the follow-up assessments were included in a special catch-up assessment. Questionnaire software had built in range and skip logic and data were transferred automatically to a database held at the University of KwaZulu-Natal.

Data analysis

There were three outcomes in the study: a) 22 knowledge statements which were scored 1 if correctly answered and 0 if not; answers were based on existing literature and guidelines (binary outcomes); b) 21 attitude questions whose responses were measured on a 5-point Likert scale - given as completely disagree (1); disagree (2); neutral (3); agree (4) and completely agree (5); positive attitudes received higher scores; and c) 19 statements on confidence item questions which were also measured on a Likert scale, scored as such: not at all confident (1), not confident (2), confident (3) and very confident (4). For both attitude and confidence domains, a participant outcome was measured by the sum of the responses to the respective items (we verified that there was not a missing response on the items). Thus, the ranges for the attitude and confidence scores were 5 to 105, and 4 to 75, respectively.

Participants baseline and follow-up characteristics and outcomes between the intervention and control areas were compared using Chi-squared tests for categorical variables and two-sample t tests for continuous measures, after confirming that data were normally distributed. To assess the effect of the proposed intervention, several analysis methods for comparing treatment effect in pre-

For peer review only - http://bmjopen.bmj.com/site/about/guidelines.xhtml

post quasi-experimental designs were considered. These include using post-measures and change from pre-treatment to post-treatment as the response variables. Approaches that use change and post measurements as the outcome , adjusting for pre-treatment measurements are recommended, and often give similar results.[25] In this paper, we considered three methods for estimating and testing the intervention effect using the sum of individual altitude or confidence scores as an outcome variable in a linear regression. Model 1 used the post-treatment measurements as the outcome variable, but adjusts for the pre-treatment values; Model 2 analyses the change score as an outcome variable with an adjustment for the pre-treatment values; and Model 3 analysed all the pre-and post-measurements as the outcome variable, and uses time (coded : 1 at follow-up and 0 at baseline) as a covariate with an interaction term for time and treatment, in addition to an adjustment for the pre-treatment values). Using Models 1 and 2, the coefficient for the intervention (coded:1 intervention group and 0: comparison group) estimates the differences in the post intervention means and differences in the mean of change of sum scores mean between of the treatment groups, controlling for the pre-treatment measurement. Using Model 3, the sum of coefficients of intervention and the interaction terms is taken as the mean difference between treatment groups post-treatment, allowing for pre-treatment mean differences between the groups. All analyses also controlled for baseline participant characteristics and prior training . The analyses were adjusted for the possible clustering effect at the site level, using a variance-correction method.[15] Data can be obtained by e-mailing the corresponding author.

Ethics

Ethics approval was obtained from the South African Medical Research Council (EC028-9/2016), the University of KwaZulu-Natal (RECIP348/17) and the WHO Ethics Review Committee (ERC0002833). Permission for undertaking the study was obtained from Tshwane and KZN Districts. Informed consent was sought from all study participants and no personal identifying information was captured in the questionnaires, only a study identification number.

Results

At baseline and follow-up, 23 intervention clinics (one large clinic was sampled twice with two rounds of data collection per time point) and 12 comparison clinics were visited; 289 and 131 health worker interviews were conducted at baseline in intervention and comparison clinics, respectively (Figure 3). Loss to follow-up between baseline and follow-up did not differ between intervention and comparison sites: 17 (13%) in comparison sites versus 36 (12.5%) in intervention sites.

Tshwane and Ugu Districts did not differ in the main outcome measures at baseline (knowledge, attitude and confidence). Additionally, they were similar in all health worker characteristics except three:: Tshwane had significantly more participants with less than 2 years employment (14.4% versus 6.2%, p=0.007), more registered nurses (57% versus 26.2%, p=0.02), and fewer lay counsellors/CHWs (7.3% versus 50.0%, respectively, p=0.02). Given the lack of significant difference in the main outcome variables at baseline, data from the two sites were combined for the analysis.

All staff approached agreed to participate. There were no significant differences between intervention and comparison sites at baseline, regarding district of origin, median age of

For peer review only - http://bmjopen.bmj.com/site/about/guidelines.xhtml

respondent, gender, cadre of health worker, and working duration (Table 1). The proportion of participants who had received previous training (through the routine health system) on specific topics was similar in intervention versus comparison sites, except for three topics which had better coverage in comparison sites (Supplementary Figure 1). These were: ever trained on how to assess and support ART adherence for HIV positive women (78.6% in intervention sites versus 89.2% in comparison sites, p=0.01); ever trained about managing breastfeeding problems (76.5% in intervention sites and 86.2% in comparison sites, p=0.02); and received any information or training about the revised infant feeding policy (55.1% in intervention sites versus 67.4% in comparison sites p=0.02). At baseline, activities around breastfeeding counselling and management were similar between comparison and intervention sites in all respects except that comparison site participants reportedly spoke more frequently to HIV positive pregnant women about feeding than intervention participants (67% versus 71.6% spoke more than 1-3 times per month, p=0.04, data not shown).

For peer review only

Table 1: Characteristics of the participants in the intervention and comparison groups at baseline

Characteristic	Intervention group (n=289) (N (%))	Comparison group (n=131) (N (%))	p-value
**District:**			
- Tshwane	152 (52.6)	56 (42.8)	0.06
- Ugu	137 (47.4)	75 (57.3)	
**Age categories:**			
- 23 to 35 years	56 (19.4)	38 (29.7)	
- 36 to 41 years	61 (21.2)	25 (19.5)	
- 42 to 46 years	53 (18.4)	26 (20.3)	0.11
- 47 to 54 years	64 (22.2)	18 (14.1)	
- Over 54 years	54 (18.8)	21 (16.4)	
**Gender**			
- Female	267 (92.7)	118 (91.5)	0.66
- Male	21 (7.3)	11 (8.5)	
**Cadre of health worker**			
- Community level worker	84 (29.5)	52 (40,0)	
- Trained health professional*	151 (53.0)	64 (49.2)	0.05
- Enrolled nurse	50 (17.4)	14 (10.8)	
**Work experience in yrs**			
- Less than 1 yr	4 (1.4)	3 (2.3)	
- 1 to <2 yrs	23 (8.0)	12 (9.3)	
- 2 to less than 5 yrs	36 (12.5)	18 (14.0)	0.20
- 5 to less than 10 yrs	71 (24.7)	43 (33.3)	
- 10 yrs or more	154 (53.5)	53 (41.1)	

Abbreviations:; *includes 68% nurses in the intervention arm and 58% nurses in comparison arm. This group also includes operation managers, dieticians, doctors and nutritionists.

In intervention sites, workshops were attended by 84-88% of participants interviewed at follow-up (Table 2).

For peer review only

Table 2: Attendance at workshops 1-3 measured at follow-up in intervention sites

	Attended workshop n	Attended catch-up n	Total attended n/N (%)
**Number of staff attending each workshop:**			
Group workshop 1	202	63	265/303 (87.5)
Group workshop 2	223	34	257/303 (84.8)
Workshop 3 (Clinical mentoring)	216	40	256/303 (84.5)
**Number of workshops attended:**			
	number	%	
No workshop	18	7.2	
1-2 workshops	10	4.0	
All 3 workshops	221	88.8	
**Total**	**249**	**100**	

Effect of the intervention on health worker knowledge

At baseline, knowledge about key infant feeding statements or facts was similar between intervention and comparison sites, except for knowledge about soft porridge (Table 3). Although at baseline, more than 90% of intervention and comparison site participants knew that a baby under 4 months should not be given soft porridge if hungry, significantly more intervention site participants knew this recommendation (Table 3). The percentage of participants at baseline correctly answering the more difficult questions (on bottle sterilisation, storing expressed breastmilk, feeding HIV exposed infants) was low (Table 3). At follow-up significantly more intervention site participants correctly answered knowledge questions, regarding the leading cause of death in children under 5, the risk of formula feeding, duration of breastfeeding for HIV-negative mothers and mothers living with HIV, how to stop breastfeeding, complementary feeding, storing expressed breastmilk, feeding whilst at work, breastfeeding and viral suppression, mixed feeding in mothers living with HIV, adherence to ART and breastfeeding, breastfeeding difficulties in mothers living with HIV and managing mothers living with HIV who are breastfeeding, than comparison site participants (Table 3). The significant differences between intervention and comparison sites regarding soft porridge were not present at follow-up. Although improvements were seen in knowledge related to the risks of mixed feeding for mothers living with HIV, most health workers still provided incorrect responses at follow-up. At baseline, the mean knowledge score was 15.0 (68%) in comparison sites versus 15.2 (69%) in intervention sites, $p=0.89$ (Table 3). At follow-up the mean knowledge score was to 15.2 (69%) in comparison sites and 17.2 (78.2%) in intervention sites, $p<0.001$ (Table 3). For the two questions measuring knowledge about the 2017 change in infant feeding guidelines, namely,
"Continued breastfeeding for 2 years is the recommended infant method in SA for ALL children, regardless of mother's HIV status" and "In South Africa, HIV-infected women who are breastfeeding should be supported to adhere to antiretroviral treatment and should introduce complementary foods around 6 months and be supported to continue breastfeeding for at least two years. (True)", there was a 36% improvement in knowledge in the intervention group at follow-up compared with a 13% increase in knowledge in the control group. For the second question there was a 15% increase in correct knowledge in the intervention group at follow-up while for the comparison group knowledge decreased from 89-81%. The difference in difference in the mean

For peer review only - http://bmjopen.bmj.com/site/about/guidelines.xhtml

knowledge scores at baseline and follow-up between intervention and control sites was significant ($p<0.001$, data not shown). At follow-up, knowledge scores of participants who attended 3 workshops compared with knowledge scores of participants who attended less than 3 workshops was significantly better ($p<0.001$).

For peer review only

For peer review only - http://bmjopen.bmj.com/site/about/guidelines.xhtml

Table 3: Knowledge of health workers about breastfeeding in the intervention and comparison sites at baseline and follow-up

Knowledge statements	Number (%) with correct answers at BASELINE			Number (%) with correct answers at FOLLOW-UP		
	Intervention (n=289)	Comparison (n=128)	p-value *	Intervention (n=250)	Comparison (n=112)	p-value *
**General breastfeeding**						
Exclusive breastfeeding is the recommended infant feeding method for ALL infants aged 0-6 months in SA, regardless of mother's HIV status (True)	271 (93.8)	118 (90.1)	0.18	234 (93.6)	102 (91.1)	0.39
Giving any formula milk during the first six months of life increases the risk of death from diarrhoea and/or pneumonia (True)	246 (85.1)	104 (79.4)	0.14	232 (92.8)	95 (84.8)	0.02
A mother who is working and giving formula milk should mix the milk herself and leave for the carer to give during the day (False)	218 (75.4)	94 (71.8)	0.42	189 (75.6)	68 (60.7)	<0.01
When sterilising feeding bottles cover the bottles with water in a saucepan and place on the heat. As soon as the water boils remove from heat and do not leave the bottles in the water until completely cool (False)	64 (22.2)	27 (20.6)	0.72	53 (21.2)	25 (22.3)	0.81
In South Africa, the leading cause of death amongst children under 5 is pneumonia (True)	189 (65.4)	82 (62.6)	0.58	230 (92.0)	75 (67.0)	<0.01
Continued breastfeeding for 2 years is the recommended infant method in SA for ALL children, regardless of mother's HIV status (True)ǂ	190 (65.7)	91 (69.5)	0.45	224 (89.6)	88 (78.6)	<0.01
A baby under 4 months should be given soft porridge once he/she seems hungry (False)	284 (98.3)	124 (94.7)	0.04	247 (98.8)	108 (96.4)	0.13
Giving a baby expressed breastmilk is not as good as breastfeeding (False)	234 (81.0)	106 (80.9)	0.99	218 (87.2)	96 (85.7)	0.70
It is safe to give the baby expressed breastmilk that has been kept outside the fridge for 8 hours (True)	106 (36.7)	43 (32.8)	0.44	120 (48.0)	38 (33.9)	<0.05
There are long term health benefits of breastfeeding for mother and child that last beyond the breastfeeding period (True)	264 (91.4)	116 (88.6)	0.37	232 (92.8)	100 (89.3)	0.26
**Breastfeeding and HIV**						
Mothers living with HIV who are receiving antiretroviral treatment and are virally suppressed should be advised not to breastfeed their infants (False)	252 (87.2)	115 (87.8)	0.87	230 (92.0)	99 (88.4)	0.27
When an HIV-infected mother is ready to add complementary feeds she should stop breastfeeding rapidly over a 24-hour period (False)	214 (74.1)	103 (78.6)	0.31	217 (86.8)	86 (76.8)	<0.05

For peer review only - http://bmjopen.bmj.com/site/about/guidelines.xhtml

If an HIV exposed baby is receiving both breastmilk and formula milk, the mother should choose either breastfeeding or formula feeding if she is adherent to ART (False)	69 (23.9)	29 (22.1)	0.70	75 (30.0)	28 (25.0)	0.33
An HIV-positive mother who is virally suppressed on antiretroviral treatment should breastfeed her child rather than not breastfeed to improve the child's survival (True)	237 (82.0)	108 (82.4)	0.91	236 (94.4)	96 (85.7)	<0.01
In South Africa, HIV-infected women who are breastfeeding should be supported to adhere to antiretroviral treatment and should be counselled and supported to exclusively breastfeed their infants for the first six months of life whilst maintaining an undetectable viral load (True) ⟊	281 (97.2)	123 (93.9)	0.10	242 (96.8)	109 (97.3)	0.79
A mother living with HIV and adherent to antiretroviral treatment cannot exclusively breastfeed her 4-month old infant because she is working. It is better for this mother to give formula during the day and breastfeed at night rather than giving no breast milk at all (True)	22 (7.6)	14 (10.7)	0.30	40 (16.0)	14 (12.5)	0.38
If a mother misses 2 doses of her ART in one month, she should be classified as a treatment failure (False)**	185 (64.0)	82 (62.6)	0.78	191 (76.4)	73 (65.2)	<0.05
An HIV-positive mother who has cracked nipples should continue to breastfeed unless they are bleeding (True)	143 (49.5)	64 (48.9)	0.91	187 (74.8)	59 (52.7)	<0.01
An HIV-exposed baby who is exclusively breastfeeding should be given some water when the weather is very hot (False)**	270 (93.4)	122 (93.13)	0.91	239 (95.6)	105 (93.8)	0.45
If a baby has a positive PCR (HIV test) at birth the mother should stop breastfeeding if this is affordable and feasible in her situation (False)**	224 (81.0)	106 (80.9)	0.99	214 (85.6)	82 (73.2)	<0.01
A mother who has missed 6 tablets of FDC in one month is considered to be poorly adherent and should stop breastfeeding immediately (False)**	181 (62.6)	89 (67.9)	0.29	201 (80.4)	72 (64.3)	<0.01
In South Africa, HIV-infected women who are breastfeeding should be supported to adhere to antiretroviral treatment and should introduce complementary foods around 6 months and be supported to continue breastfeeding for at least two years. (True)	245 (84.8)	116 (88.6)	0.30	244 (97.6)	91 (81.3)	<0.01
**Mean knowledge score (standard deviation) out of 22**	15.2 (2.6)	15.0 (3.1)	0.89*	17.2 (2.1)	15.2 (2.8)	<0.001

**The Statement is false; thus, the scales were inverted during data analysis. ⟊ - these questions measure the change in knowledge relating to the 2017 circular and updated HIV and Infant feeding guidelines
*Mann-Whitney U test comparing intervention and comparison sites at the relevant time point.
Note: the tables displays numbers with correct knowledge

For peer review only - http://bmjopen.bmj.com/site/about/guidelines.xhtml

Effect of the intervention on attitudes

At baseline, intervention and comparison sites were similar in HW attitudes except for attitudes towards feeding a crying baby and expressing breastmilk, which were significantly better in intervention sites (Supplementary Table 1). At follow-up attitudes to breastfeeding and HIV were significantly better in the intervention group for 13 of the 21 questions, and the mean attitude score towards breastfeeding was significantly better in intervention sites ($p<0.001$) (Supplementary Table 1). All three approaches to analysis demonstrated that, after controlling for other variables, final attitude (measured as attitude at follow-up, change in attitude between intervention and comparison sites or change in attitude between baseline and follow-up between intervention and comparison sites) was significantly better in intervention compared with comparison sites (Table 4). In Model 1 analysis, attitude at follow-up was 5.4 points higher in the intervention group than the comparison group; using Model 3, analysis showed a significant 5.1-point higher score in the intervention compared with the comparison group (Table 4). Model 1 demonstrated that being an enrolled nurse, and being in the youngest (36-41 years) or oldest (>54 years) age group was associated with a significantly lower attitude score; Model 3 analysis demonstrated that, controlling for other factors, trained health professionals had a significantly higher attitude score at follow-up (Table 4, $p<0.05$). We did not detect a dose-association in intervention sites when comparing 0-1 or 1-2 versus 3 workshops ($p=0.4$); but numbers in each group may have been too small to reliably assess any dose effect.

Table 4: Adjusted effect of the intervention on health worker attitude score using different methods (Effect estimate and 95% confidence interval (CI))

Variable	Model 1		Model 3	
	Effect estimate	95% CI	Effect estimate	95% CI
**Attitude score at baseline**	**0.5**	**0.3; 0.7***	N/A	N/A
**Intervention**	**5.4**	**3.9; 6.9***	**5.1**	**2.1; 8.1***
**Follow-up Period**	N/A	N/A	1.8	0.2-3.4*
**Professional role: vs community level**				
- Trained health professional	1.6	-0.05; 3.2	**4.8**	**2.8; 6.7***
- Enrolled nurse	**-2.4**	**-5.0; -0.2***	0.9	-1.4; 3.2
**Ugu District vs Tshwane District**	-0.83	-2.2; 0.5	-1.4	-3.1; 0.2
**Age category: vs 23-35 yrs**				
- 36 to 41 yrs	**-2.8**	**-5.4; -0.2***	-1.8	-4.1; 0.6
- 42 to 46 yrs	-0.9	-3.3; 1.5	-0.2	-2.5; 2.2
- 47 to 54 yrs	0.5	-2.0; 2.9	-1.2	-3.1; 0.8
- over 54 yrs	**-3.3**	**-5.7; -1.0***	-2.2	-4.8; 0.3
**Work experience <5 yrs vs ≥5yrs**	-0.3	-2.5; 1.9	-1.3	-3.4; 0.8
**Received training or information at work about the revised policy**	0.5	-1.4; 2.3	1.7	0.1-3.24
**Received any training about managing common breastfeeding problems?**	0.3	-2.5; 3.0	3.2	0.9-5.5
**Ever received any training about how to assess and**	-0.1	-2.2; 2.0	1.6	-0.5-3.8

For peer review only - http://bmjopen.bmj.com/site/about/guidelines.xhtml

**support ART adherence for HIV positive women?**				

*p<0.05 N/A: not applicable. NB: All analyses are adjusted for clustering

Effect of the intervention on confidence

At baseline there was no difference in the percentage of participants in the intervention and control sites who were confident or very confident in counselling mothers on HIV / infant feeding (Supplementary Table 2). However, at follow-up HWs from intervention sites were significantly more confident in counselling an HIV positive women, advising HIV positive omen about breastfeeding and return to school/work, advising HIV positive mothers to continue breastfeeding for two years, assessing ART adherence in HIV positive mothers, and advising HIV positive mothers about breastfeeding with cracked nipples (Supplementary table 2). The mean confidence score at follow-up was significantly higher in the intervention compared with the comparison sites at follow-up (p=0.05) (Supplementary Table 2). All three approaches to analysis demonstrated that confidence was significantly better in intervention compared with comparison sites (Table 5). Using Model 1, confidence at follow-up was 2.4 points higher in the intervention arm than the comparison arm. Using Model 3, the results showed a significant 1.5 point higher score in the intervention compared with the comparison group, although this was not significant (Table 5). Our analysis demonstrated that, controlling for other factors, being a trained health professional significantly increased confidence score by 3.1 (Model 1) or 3.7 (Model 3). Additionally, Model 3 demonstrated that, controlling for other factors, working for less than 5 years significantly reduced the confidence score. We did not measure a dose-effect (one or two versus three workshops (p=0.4)); but numbers in each group may have been too small to assess this.

Table 5: Adjusted effect of the intervention on health worker confidence scores, using different multivariable analysis methods (Effect estimate and 95% confidence interval (CI))

Variable	Model 1		Model 3	
	Effect estimate	95% CI	Effect estimate	95% CI
**Confidence score at baseline**	**0.4**	**0.3; 0.6***	N/A	N/A
**Intervention**	**2.4**	**0.3; 4.5***	1.5	-2.2; 5.1
**Follow-up time**	N/A	N/A	0.5	-1.5; 2.5
**Cadre of heath professional: vs community level**				
- Trained health professional	**3.1**	**0.3; 5.9***	**3.7**	**1.5; 5.9***
- Enrolled nurse	-0.8	-4.3; 2.7	-0.7	-3.1; 1.6
**Ugu District vs Tshwane District**	0.00	-2.1; 2.1	-1.	-3.2; 1.2
**Age category vs 23-35 yrs**				

For peer review only - http://bmjopen.bmj.com/site/about/guidelines.xhtml

- 36 to 41 yrs	-1.0	-3.7; 1.6	-0.1	-2.7; 2.5
- 42 to 46 yrs	0.3	-2.9; 3.4	0.4	-1.2; 2.9
- 47 to 54 yrs	1.4	-0.7; 3.5	-1.3	-3.4; 0.8
- over 54 yrs	-2.5	-5.7; 0.7	-0.9	-4.0; 2.2
**Work experience <5 yrs vs ≥5 yrs**	-0.5	-3.4; 2.4	**-1.9**	**-3.7; -0.2***
**Received training or information at work about the revised policy**	0.05	-1.5; 1.6	1.7	-0.3; 3.6
**Received any training about managing common breastfeeding problems?**	-0.6	-3.2; 2.1	1.8	-0.5; 4.1
**Ever received any training about how to assess and support ART adherence for HIV positive women?**	0.8	-2.1; 3.7	5.7	3.5; 7.9

*p<0.005 N/A= not applicable yrs= years. NB: All analyses are adjusted for clustering

Discussion

We demonstrate that a participatory, team-based mentoring approach to disseminating updated HIV and infant feeding guidelines was associated with an improvement in health workers' attitudes, controlling for other factors, an improvement in confidence scores, and a non-significant improvement in confidence when controlling for other factors. There was also a significant improvement in mean knowledge score between intervention and control sites at follow-up: In particular, there was a 36% improvement in knowledge of breastfeeding duration in the intervention group compared with 13% in the control group, and a 15% increase in knowledge about ART adherence and complementary feeding in the intervention group, compared with a decline in knowledge in the control group. These two questions speak specifically to the changes in the 2017 guidelines.

The mentoring approach had five distinct features: 1) it was on-site so that learning occurred in context 2) it was open to all cadres of health workers; 3) it was team-based; all participants learned together; 4) content was led by the gaps in knowledge identified by participants themselves, and 5) activities were piloted and rooted in a theoretical framework.

This mentorship approach was significantly associated with increased knowledge, especially around HIV and infant feeding, better attitudes and more confidence compared with the standard approach to disseminating infant feeding guidelines. Although some knowledge items did not change, we noted key improvements in the important knowledge items relating to HIV and infant feeding, however further reinforcement is needed regarding the issue of mixed feeding for mothers living with HIV which is possibly the biggest practice change in the 2016 guidelines. Although a few individual attitude and confidence items did not change, or only changed marginally, all our analyses indicated an improvement in follow-up attitude and confidence scores. Although the intervention group still performed poorly on some of the more difficult confidence questions such as confidence with counselling when a mother is not ART adherent, managing high viral loads during breastfeeding, explaining HIV transmission risks to a mother with a high viral load,

For peer review only - http://bmjopen.bmj.com/site/about/guidelines.xhtml

assisting mothers with HIV to safely formula feed and advising that some breastfeeding is better than no breastfeeding, we hypothesise that this could be attributed to the short duration of the intervention – three one-hour workshops over a period of three to six weeks.

There is evidence that in-service training, supervision and follow-up improves the knowledge, skills and practices of health workers managing childhood undernutrition, and can improve health worker job satisfaction and motivation, but no data exist on how to improve health worker knowledge, skills and confidence in the tricky area of HIV and infant feeding.[26-28]; however, implementation challenges have been described, including inadequately trained or shortages of supervisors, inappropriate job aids for follow-up, and poor alignment between community views/ practice and health programmes.[29] Our approach used a low technology, model for skills development at clinic level. We worked with health workers by acknowledging that they are members of their community: we discussed their fears and beliefs, and then introduced facts and evidence to extend their knowledge, change their attitudes and increase their confidence to implement updated guidelines on HIV and infant feeding. This approach can be used by routinely employed primary health care supervisors and adapted to different settings to conduct in-service training on HIV and infant feeding or other topics.

We used a team-based mentorship approach as we aimed to empower health workers.[30] A team-based approach allowed collaborative learning between different cadres of health workers, facilitating any future change in practice. In accordance with Dee Fink's theory, a participatory mentorship approach allows participants to develop foundational knowledge, apply skills, integrate ideas, develop new feelings/ interests and values and learn how to learn.[17] Our experience suggests that such an approach allowed discussion of participant's attitudes towards performing the behaviour, beliefs about whether critical, important people will approve of the behaviour (subjective norms), and about their likelihood of successfully implementing the behaviour.[19 20] Our findings corroborate a scoping review which demonstrated that mentorship improves certain quality of care outcomes [30]; in our study it improved knowledge, attitudes and confidence. However, only four studies were included in this scoping review, and the nature of the mentorship varied from video-conferencing to monthly, six-weekly or annual visits interspersed with other contact forums, conducted over one day to an entire week. A list of desirable features of mentorship interventions, include at least one dedicated mentor per facility, ensuring an adequate mentor:mentee ratio so that all staff can be supported, forming meaningful relationships between mentors and mentees, ensuring cultural congruency between mentee and mentor, and using mentors for protocol-driven programmes, such as IMCI or HIV.[30] Our intervention related to HIV and infant feeding guidelines, was low cost and low technology (one mentor working with pen, flip chart and paper in the health facility), and was implemented by a dedicated mentor from the same cultural background as the mentees. She provided onsite support during the workshops, which lasted approximately one hour, and additional support through a WhatsApp messaging group.

Given the ongoing heath worker crisis in resource limited settings, including maldistribution of staff, an imbalance in skills mix, increasingly complicated health programmes and complicated socio-cultural-political-economic environments, questions about the feasibility of an on-site

For peer review only - http://bmjopen.bmj.com/site/about/guidelines.xhtml

mentorship approach to guidelines dissemination arise. We argue that strengthening investment in on-site mentorship rather than off-site training, may be a cost-saving approach:. In our setting, all clinics receive regular visits from district primary health care (PHC) supervisors, but they mostly focus on administration and clinic management matters. These supervisors, as well as existing district PHC trainers, could be capacitated to provide clinical mentoring for health workers in the clinics they oversee. Our model of team-based learning and mentoring can be used for on-site mentoring, and avoids accommodation and travel costs, and absence from work that off-site training requires.

Our study had several limitations: We purposively selected districts for inclusion. We could not control for previous breastfeeding experience of health workers as we did not gather these data. The study tools were piloted before finalisation, but no factor analyses or validation exercises were conducted. The follow-up evaluation was conducted 3 months after the intervention; thus, we were only able to measure short term benefits and did not measure the effect of improved knowledge, attitudes and confidence on actual infant feeding practices. We could not tease out whether the relationship between number of workshops and outcomes was due to staff motivation (more motivated staff attended more workshops) or the workshops themselves. Our study's strength is that we conducted several types of analyses and all yielded congruent results.

Conclusion: We demonstrated improved knowledge, attitudes and confidence of health workers following a participatory mentorship approach to HIV and infant feeding guideline dissemination compared with a standard approach. More research is needed to better understand how to change health worker practices, which may then improve breastfeeding practices.

For peer review only

For peer review only - http://bmjopen.bmj.com/site/about/guidelines.xhtml

Authors contributions:

AG: Study conceptualization and tool development, protocol writing including intervention development, oversight of sampling and field work, writing of the first draft of this manuscript, receiving and incorporating co-author comments, finalization of the paper
TD: Study conceptualization and tool development, protocol writing including intervention development, set up the sample frame and sampling, contributed to the manuscript, reviewed and approved the final version of the manuscript
SM: Led the statistical components of the protocol; provided overall oversight on the statistical analysis, contributed to the manuscript, reviewed and approved the final version of the manuscript
TN: Performed the work on the statistical components of the protocol, under SM's guidance; provided data analysis under SM's guidance, contributed to the manuscript, reviewed and approved the final version of the manuscript
LH: Contributed to study conceptualization and tool development, protocol writing including intervention development; was overall Project Manager; established, managed and cleaned the database; contributed to the manuscript, reviewed and approved the final version of the manuscript
VJ: Provided guidance on intervention development. Contributed to the manuscript, reviewed and approved the final version of the manuscript
IMSE: Contributed to study conceptualization and tool development, protocol writing including intervention development; contributed to the manuscript, reviewed and approved the final version of the manuscript
UF: Contributed to study conceptualization, assisted with district level buy-in in Tshwane District, provided information on how routine dissemination of updated infant feeding guidelines; contributed to the manuscript, reviewed and approved the final version of the manuscript
MAD: Contributed to study conceptualization and tool development; contributed to the manuscript, reviewed and approved the final version of the manuscript
NR: Contributed to study conceptualization; contributed to the manuscript, reviewed and approved the final version of the manuscript
MK: Contributed to study conceptualization and tool development; contributed to the manuscript, reviewed and approved the final version of the manuscript
DS: Contributed to study conceptualization and tool development; contributed to the manuscript, reviewed and approved the final version of the manuscript
SK: Contributed to study conceptualization, assisted with national level buy-in, provided information on how routine dissemination of updated infant feeding guidelines; contributed to the manuscript, reviewed and approved the final version of the manuscript
TT: Contributed to study conceptualization and tool development; contributed to the manuscript, reviewed and approved the final version of the manuscript
CH: Study conceptualization, protocol writing including intervention development, high level oversight of study implementation, contributed to the manuscript, reviewed and approved the final version of the manuscript

Data sharing statement: Data can be obtained by e-mailing the corresponding author, and upon reasonable request

For peer review only - http://bmjopen.bmj.com/site/about/guidelines.xhtml

Figure legends:

Figure 1: Study districts: Tshwane District in Gauteng Province and Ugu District in KwaZulu-Natal Province of South Africa

Figure 2: Theoretical frameworks which informed the development of the intervention

Figure 3: Study population at baseline and follow-up for intervention and comparison sites

Supplementary Figure 1: Prior training reported by participants at baseline (intervention versus comparison groups)

For peer review only

References

1. McCormack L, Sheridan S, Lewis M, et al. Communication and dissemination strategies to facilitate the use of health-related evidence. Secondary Communication and dissemination strategies to facilitate the use of health-related evidence. 2013. https://www.ncbi.nlm.nih.gov/books/NBK179104/pdf/Bookshelf_NBK179104.pdf.
2. Victora C, Bahl R, Barros A, et al. Breastfeeding in the 21st century: epidemiology, mechanisms, and lifelong effect. The Lancet 2016;**387**(10017):475-90
3. Black RE, Allen LH, Bhutta ZA, et al. Maternal and child undernutrition: global and regional exposures and health consequences. Lancet. 2008.;**37**(9608):243-60
4. World Health Organisation. Antiretroviral drugs for treating pregnant women and preventing HIV infection in infants: towards universal access. Recommendations for a public health approach (2010 revision). Available from http://www.who.int/hiv/pub/mtct/arv_guidelines_mtct.pdf. Accessed 22 May 2020.
5. Six week extended dose nevirapine (SWEN) Study team et al. Extended dose nevirapine at 6 weeks of age for infants to prevent HIV transmission via breastfeeding in Ethiopia, India and Uganda: an analysis of 3 randomised controlled trials. Lancet 2008;**372**:300-13
6. Jamieson D, Chasela C, Hudgens M, et al. Maternal and infant antiretroviral regimens to prevent postnatal HIVtransmission: 48 weeks follow-up of the BAN randomised controlled trial. Lancet 2012;**379**:2449-258
7. National Department of Health Breastfeeding Summit. The Tshwane Declaration of Support for breastfeeding in South Africa. S Afr J Clin Nutr 2011;**24**(4):214-16
8. National Department of Health. National conslidated guidelines for the prevention of mother to child transmission of HIV (PMTCT) and the management of HIV in children, adolescents and adults. . Available from https://www.health-e.org.za/2015/07/02/guidelines-national-consolidated-guidelines-for-pmtct-and-the-management-of-hiv-in-children-adolescents-and-adults/. Accessed 22 May 2020.
9. World Health Organisation, UNICEF. 2016 Guideline Update on HIV and Infant Feeding. Available from https://www.who.int/nutrition/publications/hivaids/guideline_hiv_infantfeeding_2016/en/. Accessed 22 May 2020.
10. Academy of Nutrition and Dietetics, Handu D, Acosta A, et al. Effectiveness of interventions to promote exclusive breastfeeding in women living with HIV that are on antiretroviral therapy living in areas that promote exclusive breastfeeding due to limited resources for safe replacement feeding. 2016. Available from https://www.who.int/maternal_child_adolescent/documents/hiv-infant-feeding-2016-ax2-breastfeeding-promotion_systematic-review.pdf?ua=1. Accessed 22 May 2020.
11. World Health Organization, United Nations Children's Fund. Guideline: updates on HIV and infant feeding: the duration of breastfeeding, and support from health services to improve feeding practices among mothers living with HIV. Avalable from http://apps.who.int/iris/bitstream/handle/10665/246260/9789241549707-eng.pdf;sequence=1. Accessed 22 May 2020.

For peer review only - http://bmjopen.bmj.com/site/about/guidelines.xhtml

12. Barker M, Baird J, Lawrence W, et al. The Southampton Initiative for Health: a complex intervention to improve the diets and increase the physical activity levels of women from disadvantaged communities. . J Health Psychol 2011;**16**:178-91
13. Baird J, Jarman M, Lawrence W, al. e. The effect of a behaviour change intervention on the diets and physical activity levels of women attending Sure Start Children's Centres: results from a complex public health intervention. . BMJ Open 2014;**4:e005290.**
14. Janse van Rensburg L, Nel R, Walsh CM. Knowledge, opinions and practices of healthcare workers related to infant feeding in the context of HIV. Health SA Gesondheid 2016;**21**:129-36 doi: http://dx.doi.org/10.1016/j.hsag.2015.12.001[published Online First: Epub Date]|.
15. Eldridge S, Ashby D, Kerry S. Sample size for cluster randomized trials: effect of coefficient of variation of cluster size and analysis method. International Journal of Epidemiology 2006;**35**(1292-1300):doi:10.1093/ije/dyl129
16. Horwood C, Haskins L, Goga A, et al. An educational intervention to update health workers about HIV and infant feeding. . Maternal & Child Nutrition. 2019:https://doi.org/10.1111/mcn.12922
17. GW. P, Gouws E, Bryce J, Burnham G, Team FtUIIS. Improving facility-based care for sick children in Uganda: training is not enough ⍰ Health Policy Plan 2005;**20**(Suppl_1):i58-i68
18. Dee Fink L. A self-directed guide to designing courses for significant learning. Secondary A self-directed guide to designing courses for significant learning 2003. https://www.deefinkandassociates.com/GuidetoCourseDesignAug05.pdf.
19. Ajzen I. The theory of planned behavior. Organizational Behavior and Human Decision Processes 1991;**50**(2):179-211 doi: http://dx.doi.org/10.1016/0749-5978(91)90020-T[published Online First: Epub Date]|.
20. Ajzen I. Perceived Behavioral Control, Self-Efficacy, Locus of Control, and the Theory of Planned Behavior1. Journal of Applied Social Psychology 2002;**32**(4):665-83 doi: 10.1111/j.1559-1816.2002.tb00236.x[published Online First: Epub Date]|.
21. World Health Organization, UNICEF. Breastfeeding Counselling: A Training Course. Geneva, 1993. Available from https://www.who.int/maternal_child_adolescent/documents/who_cdr_93_3/en/. Accessed 22 May 2020.
22. World Healh Organization, UNICEF. HIV and Infant Feeding Counselling: A Training Course. Geneva, 2000. Available from https://apps.who.int/iris/bitstream/handle/10665/66194/WHO_FCH_CAH_00.4.pdf;sequence=5. Accessed 22 May 2020.
23. Doherty T, Sanders D, Goga A, Jackson D. Implications of the new WHO Guidelines on HIV and infant feeding for child survival in South Africa. . Bull World Health Organ. 2011;**89**:62-67
24. Goga A, Doherty T, Jackson D, et al. Infant feeding practices at routine PMTCT sites, South Africa: Results of a prospective observational study amongst HIV exposed and unexposed infants - birth to 9 months. Int Breastfeeding J. 2012;**7**(4):doi:10.1186/746-4358-7-4.

For peer review only - http://bmjopen.bmj.com/site/about/guidelines.xhtml

25. O'Connell N, Dai L, Jiang Y, Speiser J, Ward R, et al. Methods for Analysis of Pre-Post Data in Clinical Research: A Comparison of Five Common Methods. . J Biom Biostat 2017;**8**(334):doi:10.4172/2155-6180.1000334
26. Sunguya B, Poudel K, Mlunde L, Urassa D, Yasuoka J, Jimba M. Nutrition Training Improves Health Workers' Nutrition Knowledge and Competence to Manage Child Undernutrition: A Systematic Review. Frontiers in public health 2013:10.3389/fpubh.2013.00037
27. Bailey C, Blake C, Schriver M, Cubaka V, Thomas T, Martin Hilber A. A systematic review of supportive supervision as a strategy to improve primary healthcare services in Sub-Saharan Africa. Int J Gynaecol Obstet. 2016;**132**(1):117-25. doi: 10.1016/j.ijgo.2015.10.004.
28. World Health Organisation. Management of Childhood Illness in Developing Countries: Rationale for an integrated approach. 1999. Available from http://www.who.int/child_adolescent_health/documents/chs_cah_98_1a/en/. Accessed 22 May 2020.
29. Goga A, Muhe L. Global Challenges with Scale-Up of the Integrated Management of Childhood Illness Strategy: Results of a multi-country Survey. . BMC Public Health. 2011;**11**(503):doi: 10.1186/471-2458-11-503
30. Schwerdte P, Morphet, Hall H. A scoping review of mentorship of health personnel to improve the quality of health care i low and middle-income countries. Globalisation and Health 2017;**13**(77):doi 10.1186/s12992-017-0301-1

For peer review only

For peer review only - http://bmjopen.bmj.com/site/about/guidelines.xhtml

Figure 1: Study districts: Tshwane Metropolitan Municipality in Gauteng Province and Ugu District in KwaZulu-Natal Province of South Africa

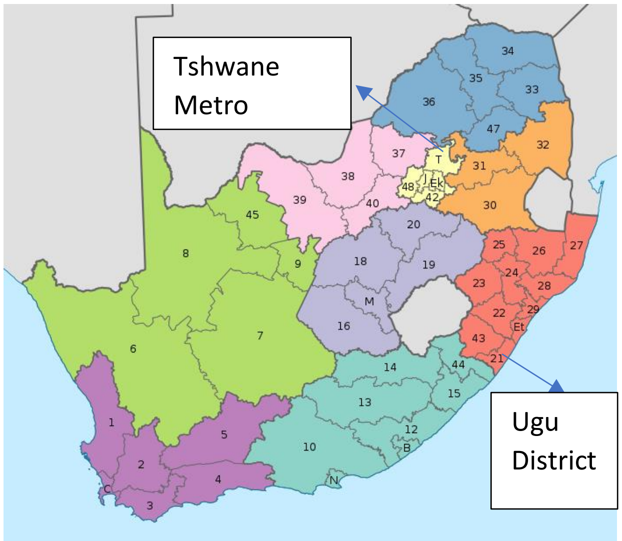

For peer review only - http://bmjopen.bmj.com/site/about/guidelines.xhtml

Figure 2: Theoretical frameworks which informed the development of the intervention

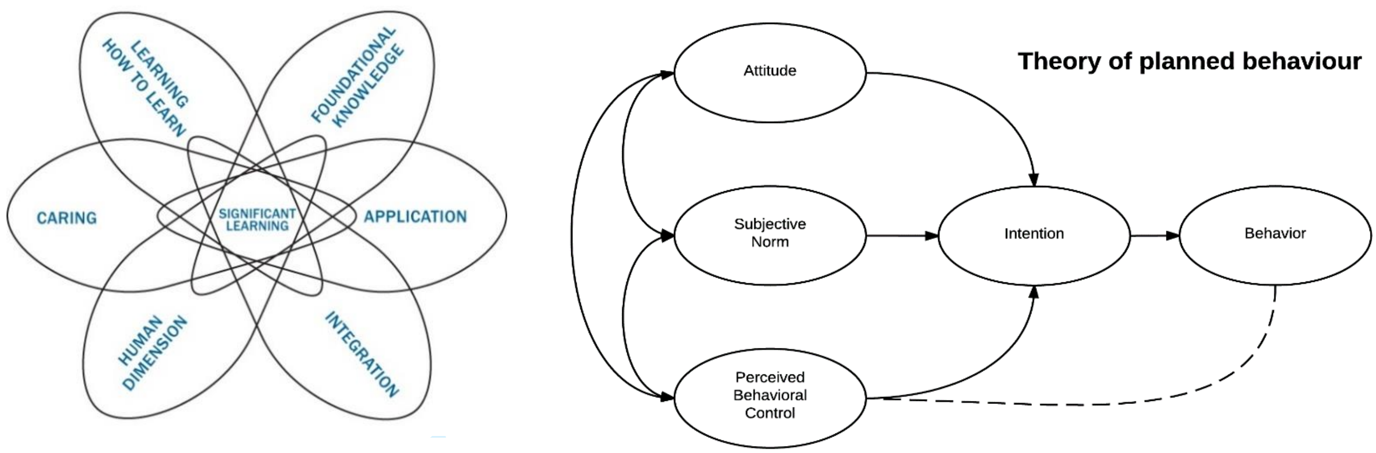

Dee Fink's six-part taxonomy for significant learning

For peer review only

Figure 3: Study population at baseline and follow-up for intervention and comparison sites

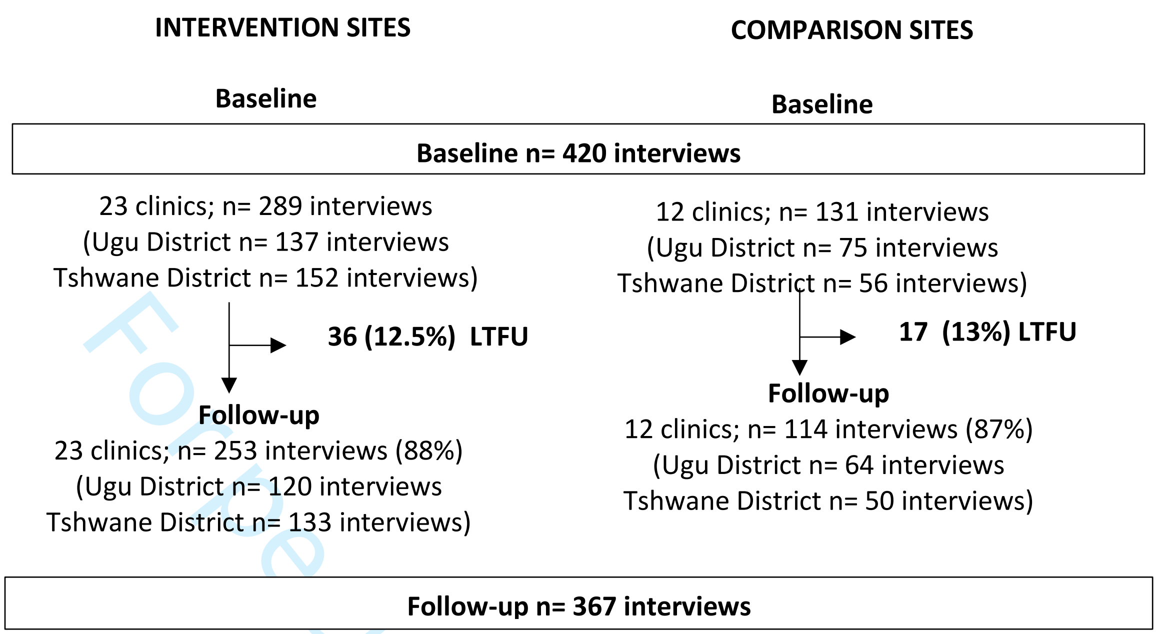

LTFU: lost to follow up

For peer review only

Supplementary Figure 1: Prior training reported by participants at baseline (intervention versus comparison groups)

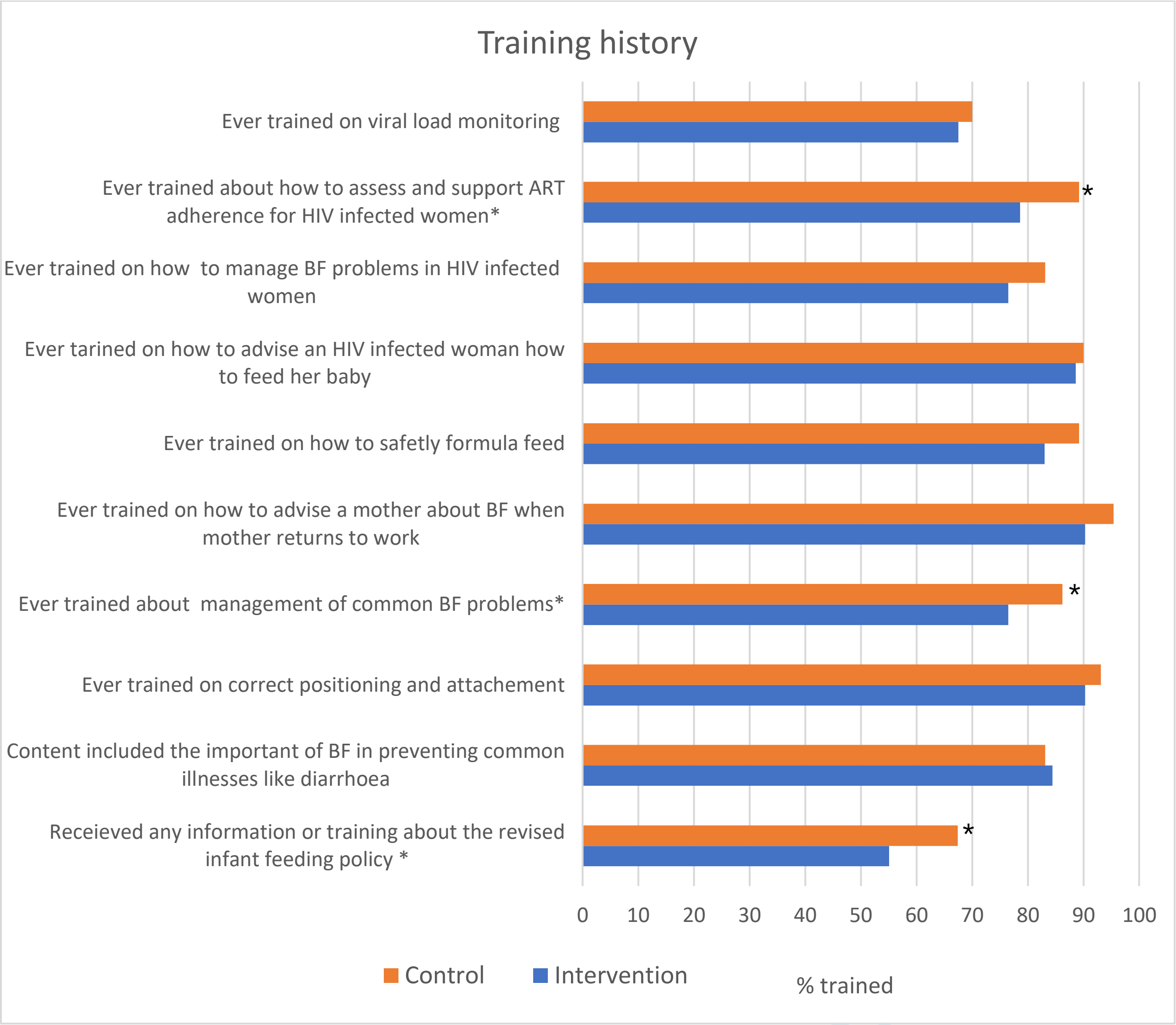

*p<0.05

Abbreviations: ART= antiretroviral therapy; BF= breastfeeding

Supplementary Table 1: Attitude scores of health workers at baseline and follow-up

Attitude statements	Number (%) who agreed or strongly agreed with the statement at baseline			Number (%) who agreed or strongly agreed with the statement at follow-up		
	Intervention (n=289)	Comparison (n=131)	p-value *	Intervention (n=252)	Comparison (n=114)	p-value*
There have been so many changes to the infant feeding guidelines and breastfeeding guidelines however I am NOT confused about what to tell mothers who are HIV-infected about breastfeeding	155 (53.6)	74 (56.9)	059	175 (69.4)	65 (57.0)	0.02
When a baby cries all the time it is NOT usually because the baby is hungry and needs more food than just breastmilk	260 (90.0)	107 (83.0)	0.02	230 (91.3)	91(79.8)	<0.01
Exclusive breastfeeding in the first 6 months of life is the best choice for all mothers and babies in South Africa	256 (88.6)	114 (88.4)	0.65	239 (94.8)	98 (86.0)	<0.01
For an HIV-exposed infant any breastfeeding is better than no breastfeeding at all, as long as the mother is virally suppressed and on antiretroviral therapy	189 (65.4)	79 (61.2)	0.31	196 (77.8)	74 (64.9)	0.01
The benefits of breastfeeding for protecting children from illness such as diarrhoea and pneumonia outweighs the risk of acquiring HIV if the mother is on antiretroviral treatment	224 (77.5)	93 (72.1)	0.15	218 (86.5)	87 (76.3)	0.02
I feel that an HIV-infected mother who has not disclosed to her partner is NOT at high risk of non-adherence to ART and should NOT stop breastfeeding as soon as possible	128 (44.3)	51 (39.5)	0.30	137 (54.4)	46 (40.4)	0.01
I should support all mothers, regardless of HIV status, to continue breastfeeding until 2 years, as long as HIV-infected women are virally suppressed	237 (82.0)	101 (78.3)	0.24	237 (94.1)	87 (76.3)	<0.01
I should NOT advise an HIV-positive virally suppressed mother who has cracked and bleeding nipples to temporarily stop breastfeeding	80 (27.7)	40 (31.3)	0.55	97 (38.5)	24 (21.1)	<0.01
HIV-exposed babies who are PCR negative must NOT stop breastfeeding as soon as possible	238 (82.4)	104 (81.3)	0.47	221 (87.7)	95 (83.3)	0.26
Formula feeding is NOT the best choice for mothers living in good socio-economic circumstances who are going back to work	202 (70.0)	95 (74.2)	0.58	198 (78.6)	74 (64.9)	<0.01
For an HIV-positive mother on antiretroviral treatment and virally suppressed mixed feeding is better than not breastfeeding at all	39 (13.5)	15 (11.7)	0.56	57 (22.6)	16 (14.0)	0.06
Exclusive breastfeeding for six months is an achievable goal for the majority of mothers	249 (86.2)	105 (82.0)	0.11	227 (90.1)	96 (84.1)	0.11
It is safer for HIV-positive mothers to breastfeed than to formula feed	231 (79.9)	89 (69.5)	0.63	226 (89.7)	85 (74.6)	<0.01

For peer review only - http://bmjopen.bmj.com/site/about/guidelines.xhtml

In our community working mothers can successfully maintain exclusive breast feeding while going to work	219 (75.8)	89 (69.5)	0.09	208 (82.5)	92 (80.7)	0.67
An HIV-positive mother who is on ART and not virally suppressed and is mixed feeding is putting her child at risk of acquiring HIV	256 (88.6)	109 (85.2)	0.13	219 (86.9)	97 (85.1)	0.64
It is NOT very difficult for mothers to express breastmilk while they are at work or school	164 (53.0)	57 (44.5)	0.01	1689(67.1)	51 (44.7)	<0.01
If an HIV-positive mother can afford to buy formula it is NOT better for her to formula feed her baby	170 (58.8)	83 (64.9)	0.38	185 (73.4)	68 (59.7)	<0.01
Promoting breastfeeding for two years for HIV-exposed infants is NOT a risk because mothers will be able to maintain good ART adherence for that long	197 (68.2)	85 (66.4)	0.51	210 (83.3)	79 (69.3)	<0.01
In South Africa it is possible to improve exclusive breastfeeding rates	244 (84.7)	108 (84.4)	0.61	230 (91.3)	97 (85.1)	0.08
There are exceptional circumstances where an HIV-positive mother would be advised not to breastfeed, such as failure of 2nd or 3rd line ART treatment, but these are not common	225 (77.9)	93 (72.7)	0.13	201 (79.7)	87 (76.3)	0.46
Formula feeding is NOT more convenient for a mother than breastfeeding	253 (87.5)	109 (85.2)	0.23	211 (83.7)	100(87.7)	0.32
**Number (%) participants whose attitude was to at least agree (Attitude score ≥84)***	**71 (24.6)**	**23 (17.9)**	**0.12**	**123 (49.2)**	**27 (24.1)**	**<0.01**
**Mean attitude score out of 105 (95% CI)**	**76.9 (75.9 – 77.9)**	**75.0 (73.0 – 77.0)**	**0.07**	**82.7 (81.6 – 83.8)**	**76.8 (75.0 – 78.5)**	**<0.01**

*84 was the minimum score obtainable if a participant at least agreed with all statements

For peer review only - http://bmjopen.bmj.com/site/about/guidelines.xhtml

Supplementary Table 2: Confidence statements of health workers at baseline and follow-up

Confidence statements	Number (%) who felt confident or very confident at baseline			Number (%) who felt confident or very confident at follow-up		
	Intervention (n=289)	Comparison (n=131)	p-value	Intervention (n=252)	Comparison (n=114)	p-value
How confident do you feel about counselling an HIV-positive pregnant woman about how she will feed her baby	265 (91.7)	116 (88.6)	0.30	240 (95.2)	100 (87.7)	0.01
How confident do you feel about giving information about the risks and benefits of breastfeeding to an HIV-infected mother	268 (92.7)	120 (91.6)	0.67	238 (94.4)	102 (89.5)	0.09
How confident do you feel about assessing whether there is good positioning and attachment during breastfeeding	263 (91.0)	125 (95.4)	0.11	243(96.4)	107 (93.9)	0.27
How confident do you feel about advising an HIV-positive mother about how to continue to breastfeed her baby when she returns to work or school	258 (89.3)	117 (89.3)	0.99	242 (96.0)	101 (88.6)	<0.01
How confident do you feel about advising an HIV-infected mother who is virally suppressed who is mixed feeding her infant	243 (84.1)	113 (86.3)	0.57	226 (89.7)	99 (86.8)	0.43
How confident do you feel about advising an HIV-infected mother to continue breastfeeding for two years	216 (74.7)	105 (80.2)	0.23	236 (93.6)	93 (81.6)	<0.01
How confident do you feel about advising an HIV-infected mother about how to stop breastfeeding	214 (74.1)	89 (67.9)	0.20	188 (74.6)	86 (75.4)	0.87
How confident do you feel about advising an HIV-positive mother about starting complementary feeds	251 (86.9)	115 (87.8)	0.79	229 (90.9)	103 (90.4)	0.87
How confident do you feel about assessing ART compliance in an HIV-positive mother	240 (83.1)	111 (84.7)	0.67	230 (91.3)	94 (82.5)	0.02
How confident do you feel about identifying when an HIV-positive mother is not adhering to her ART treatment	224 (77.5)	104 (79.4)	0.67	218 (86.5)	90 (79.0)	0.07
How confident do you feel about reassuring a mother living with HIV who is virally suppressed that a shorter duration of breastfeeding is better than never initiating breastfeeding	227 (78.5)	103 (78.6)	0.99	215 (85.3)	96 (84.2)	0.78
How confident do you feel about explaining the risks of HIV transmission through breastmilk to an HIV-infected mother with high viral load	247 (85.5)	114 (87.0)	0.67	220 (87.3)	105 (92.1)	0.17
How confident do you feel about assisting a mother with HIV to safely formula feed her baby	212 (73.4)	102 (77.9)	0.33	191 (75.8)	95 (83.3)	0.11

For peer review only

How confident do you feel about advising an HIV-infected mother who is exclusively breastfeeding and has cracked nipples with bloody milk about how to feed her baby	196 (67.8)	84 (64.1)	0.45	201 (79.8)	80 (70.2)	0.04
How confident do you feel about using the guidelines for safe replacement feeding when you counsel a mother who is not adherent to ART and has a viral load above 1000 copies/ml	191 (66.1)	99 (75.6)	0.05	185 (73.4)	75 (65.8)	0.14
How confident do you feel about advising an HIV-infected mother who is exclusively breastfeeding and has defaulted from her ART about how to feed her baby	205 (70.9)	97 (74.1)	0.51	189 (75.0)	80 (70..2)	0.33
How confident do you feel about explaining to a mother about expressing and storing milk	269 (93.1)	124 (94.7)	0.54	237 (94.1)	109 (95.6)	0.54
How confident do you feel about managing poor ART compliance in an HIV-infected breastfeeding mother	215 (74.4)	104 (79.4)	0.27	209 (82.9)	89 (78.1)	0.27
A mother is not adherent to ART and her last viral load is 1000 copies per ml. How confident do you feel about counselling her about feeding her infant?	199 (68.9)	98 (74.8)	0.21	198 (78.6)	80 (70.2)	0.08
**Number (%) participants who were confident or very confident (Sum Score ≥ 57)**	**164 (56.8)**	**86 (67.2)**	**0.09**	**175 (70.0)**	**72 (64.3)**	**0.28**
**Mean confidence score out of 76 (95% CI)**	**59.1 (58.0-60.2)**	**59.1 (57.0-61.3)**	**1.0**	**61.2 (60.8-63.1)**	**59.9 (58.1-61.7)**	**0.05**

For peer review only - http://bmjopen.bmj.com/site/about/guidelines.xhtml

BMJ Open

Translating new evidence into clinical practice: A quasi-experimental controlled before-after study evaluating the effect of a novel outreach mentoring approach on knowledge, attitudes and confidence of health workers providing HIV and infant feeding counselling in South Africa

Journal:	*BMJ Open*
Manuscript ID	bmjopen-2019-034770.R2
Article Type:	Original research
Date Submitted by the Author:	29-Jun-2020

Complete List of Authors:	Goga, Ameena; Medical Research Council of South Africa, Health Systems Research Unit and HIV Prevention Research Unit; University of Pretoria Faculty of Health Sciences, Paediatrics<br>Doherty, Tanya; South African Medical Research Council<br>Manda, Samuel; South African Medical Research Council<br>Nkwenika, Tshifhiwa; SAMRC<br>Haskins, Lyn; University of KwaZulu-Natal<br>John, Vaughn; University of KwaZulu-Natal, School of Education<br>Engebretsen, Ingunn; University of Bergen Faculty of Medicine and Dentistry, Centre for International Health<br>Feucht, Ute; University of Pretoria<br>Dhansay, Ali; Burden of Disease Research Unit<br>Rollins, Nigel; World Health Organization, Department of Maternal, Newborn, Child and Adolescent Health<br>Kroon, Max; University of Cape Town Faculty of Health Sciences, Neonatology<br>Sanders, David; University of the Western Cape, South Africa , School of Public Health<br>Kauchali, Shuaib; South African National Department of Health<br>Tylleskär, Thorkild; Universitetet i Bergen, Centre for International health<br>Horwood, Christiane ; University of KwaZulu-Natal,

**Primary Subject Heading**:	Global health
Secondary Subject Heading:	Public health
Keywords:	Public health < INFECTIOUS DISEASES, HIV & AIDS < INFECTIOUS DISEASES, Community child health < PAEDIATRICS

I, the Submitting Author has the right to grant and does grant on behalf of all authors of the Work (as defined in the below author licence), an exclusive licence and/or a non-exclusive licence for contributions from authors who are: i) UK Crown employees; ii) where BMJ has agreed a CC-BY licence shall apply, and/or iii) in accordance with the terms applicable for US Federal Government officers or employees acting as part of their official duties; on a worldwide, perpetual, irrevocable, royalty-free basis to BMJ Publishing Group Ltd ("BMJ") its licensees and where the relevant Journal is co-owned by BMJ to the co-owners of the Journal, to publish the Work in this journal and any other BMJ products and to exploit all rights, as set out in our licence.

The Submitting Author accepts and understands that any supply made under these terms is made by BMJ to the Submitting Author unless you are acting as an employee on behalf of your employer or a postgraduate student of an affiliated institution which is paying any applicable article publishing charge ("APC") for Open Access articles. Where the Submitting Author wishes to make the Work available on an Open Access basis (and intends to pay the relevant APC), the terms of reuse of such Open Access shall be governed by a Creative Commons licence – details of these licences and which Creative Commons licence will apply to this Work are set out in our licence referred to above.

Other than as permitted in any relevant BMJ Author's Self Archiving Policies, I confirm this Work has not been accepted for publication elsewhere, is not being considered for publication elsewhere and does not duplicate material already published. I confirm all authors consent to publication of this Work and authorise the granting of this licence.

For peer review only

Translating new evidence into clinical practice: A quasi-experimental controlled before-after study evaluating the effect of a novel outreach mentoring approach on knowledge, attitudes and confidence of health workers providing HIV and infant feeding counselling in South Africa

Ameena Goga[1, 2, 3], Tanya Doherty[1,4,5], Samuel Manda[6, 7], Tshifhiwa Nkwenika[6], Lyn Haskins[8], Vaughn John[9], Ingunn M.S. Engebretsen[10], Ute Feucht[2, 11,12, 13], Ali Dhansay[14, 15] Nigel Rollins[16], Max Kroon[17, 18], David Sanders[4,19], Shuaib Kauchali[20], Thorkild Tylleskar[10,] Christiane Horwood[8]

Affiliations

[1]Health Systems Research Unit, South African Medical Research Council, South Africa
[2]Department of Paediatrics, University of Pretoria, South Africa
[3]HIV Prevention Research Unit, South African Medical Research Council, South Africa
[4]School of Public Health, University of the Western Cape, South Africa
[5]School of Public Health, University of the Witwatersrand
[6]Biostatistics Unit, South African Medical Research Council, South Africa
[7]Department of Statistics, University of Pretoria, South Africa
[8]Centre for Rural Health, University of KwaZulu-Natal, South Africa
[9]School of Education, University of KwaZulu Natal, Pietermaritzburg, South Africa
[10]Centre for International Health, University of Bergen, Norway
[11]Tshwane District Health Services, Gauteng Department of Health, city? South Africa
[12]Research Centre for Maternal, Fetal, Newborn and Child Health Care Strategies, University of Pretoria, Pretoria, South Africa
[13]Maternal and Infant Health Care Strategies Research Unit, South African Medical Research Council, South Africa
[14]Division of Human Nutrition and Department of Paediatrics and Child Health, Stellenbosch University, South Africa.
[15]Burden of Disease Research Unit, South African Medical Research Council, South Africa
[16]Department of Maternal, Newborn, Child and Adolescent Health, World Health Organization, Geneva, Switzerland
[17]Department of Neonatology, Faculty of Health Sciences, University of Cape Town
[18]Mowbray Maternity Hospital, Cape Town, South Africa
[19]Department of Paediatrics and Child Health, University of Cape Town, South Africa
[20]National Department of Health, South Africa

Corresponding author: Ameena.Goga@mrc.ac.za

Key words:

For peer review only

Abstract

Objectives: We report the effectiveness of a mentoring approach to improve health workers' knowledge, attitudes and confidence with counselling on HIV and infant feeding.
Design: A quasi-experimental controlled before-after study.
Setting: Primary health care clinics were randomly selected (n=24 intervention, n=12 comparison) from two districts, South Africa.
Participants: All health workers (HW) providing infant feeding counselling in selected facilities were invited to participate.
Interventions: three 1-2 hour, on-site workshops were conducted over 3-6 weeks.
Primary outcome measures: Knowledge, attitude and confidence scores (separately) scores. To estimate the effect of the intervention the sum of attitude or confidence scales were modeled using a linear regression. This allowed us to estimate the mean score difference between treatment groups post-intervention, adjusting for the mean score difference between groups at baseline. Analyses were adjusted for baseline characteristics of the participants and clustering at the health facility level.
Results: In intervention and comparison sites, respectively: 289 and 131 baseline and 253 and 114 follow-up interviews were conducted (August-December 2017). At baseline there was no difference in mean number of correctly answered knowledge questions (out of 22), but this differed significantly at follow-up (15.2 in comparison and 17.2 in intervention sites ($p<0.001$)). At follow-up, the mean attitude and confidence scores towards breastfeeding were significantly better in intervention sites versus comparison sites ($p<0.001$ and $p=0.05$, respectively). Controlling for confounders, interactions between time and intervention status and pre-intervention values, attitude score was 5.1-points significantly higher for intervention compared with comparison groups.
Conclusion: A participatory, low intensity on-site mentoring approach to disseminating updated infant feeding guidelines improved HWs' knowledge, attitudes and confidence more than standard dissemination via a circular. Further research is required to evaluate the effectiveness, feasibility and sustainability of implementing such a mentoring approach at scale.

Strengths and Limitations of this study:

1. Fieldwork was conducted in two geographically and historically different provinces, facilitating generalisability of results.
2. The intervention was participatory, low intensity, on-site and integrated into routine facilities.
3. Several types of analyses were conducted which all yielded congruent results.
4. However, limitations were that (i) we purposively selected districts for inclusion (ii) we could not control for HWs' personal breastfeeding experience as we did not gather these data (iii) the follow-up evaluation was undertaken 3 months after the intervention - thus, we measured short term benefits, and (iv) we did not measure the direct effect of improved HWs' knowledge, attitudes and confidence on health workers' counselling and mothers' infant feeding practices and (v) we did not co-design the intervention with women living with HIV.
5. The finding that knowledge scores amongst participants who attended 3 workshops were significantly better than knowledge scores amongst participants who attended less than 3

For peer review only - http://bmjopen.bmj.com/site/about/guidelines.xhtml

workshops, may simply reflect better motivation amongst attendees of more workshops, rather than the effect of the workshops themselves. We could not tease out these effects.

Funding statement

This study was funded by the World Health Organization (Ref: 2017/712509-0). Tanya Doherty receives a from the National Research Foundation, South Africa. Ameena Goga and Tanya Doherty's time was funded by the South African Medical Research Council. The publication of paper was funded by the South African Medical Research Council.

Competing interests statement

No author has declared any competing interests.

Introduction

The benefits of breastfeeding in all settings, and particularly in low-middle income settings with high HIV prevalence, are undisputed.[1 2] Policies and clinical practice guidelines on preventing mother to child transmission of HIV (PMTCT) and infant feeding have undergone frequent evidence-based revisions. For example South African PMTCT policy and its accompanying infant feeding recommendations have been revised five times since 2001 (2008, 2010, 2013, 2015, 2019).[3-7]. Additionally, in 2011 a national infant feeding declaration withdrew free commercial infant formula as part of the PMTCT programme [8], and in 2017 the infant and young child feeding policy was updated to recommend that women living with HIV may continue breastfeeding for up to 24 months or longer (similar to the general population) while being fully supported for antiretroviral therapy (ART) adherence. This followed a 2016 World Health Organization (WHO) update which also stated that mixed feeding is not a reason to stop breastfeeding in the presence of ARV drugs.[9] However, a key gap is that these policies have not been effectively communicated to all health workers – a requirement of the Mother-Baby Friendly Initiative.[10 11] Health workers play a critical role in guiding infant feeding choices and sustaining infant feeding practices [11-13]; they wield power and authority [12 14] but their potentially positive influence on infant feeding is compromised by confusion over HIV and infant feeding, which has eroded their confidence.[11 13] Identifying and implementing optimal sstrategies to effectively disseminate updated guidelines have lagged behind. Multi-component dissemination strategies, which aim to increase the reach, ability and motivation of health workers, are more effective than one strategy alone.[15] However, in reality there are few published studies that inform guideline dissemination. Most of these are from high-income settings and may not be relevant to low-income settings which have unique challenges.[15]

Research has demonstrated that improving HWs' capacity can significantly improve their skills, self-efficacy and confidence to counsel, support and promote breastfeeding among women living with HIV.[16 17] Consequently, a key question was: What learning approach could best develop health worker capacity and confidence to implement the updated HIV and infant feeding guideline, using a methodology that is sustainable and feasible to implement at scale? Pedagogical research

For peer review only - http://bmjopen.bmj.com/site/about/guidelines.xhtml

For peer review only

highlights the advantage of participatory training compared with standard didactic teaching for improving health worker skills.[18 19] Thus, we sought to determine whether a participatory outreach mentorship approach to disseminate the updated HIV and infant feeding guidelines, using simple low-technology activities, improves health workers' knowledge of, attitudes towards and confidence with counselling on HIV and infant feeding. We chose to focus on health workers knowledge, attitudes and confidence as health workers in South Africa consider themselves as advocates for babies. [20] Additionally, they are one of the key influential groups in the complex socio-ecology of infant feeding. [12 13 21 22]

Methods

Study design

A quasi-experimental before-after design with intervention and comparison sites was used. Two purposively-selected districts (Ugu and Tshwane District) in South Africa in each of two geographically disparate provinces, KwaZulu-Natal (KZN) and Gauteng (Figure 1), were included. Both provinces experienced a policy change in June 2017, when the 2013 South African Infant and Young Child Feeding Policy was amended to align with the 2016 WHO/UNICEF update on HIV and Infant feeding guideline.

Sampling

In Ugu District all four sub-districts were selected; within Tshwane District two of the seven service delivery regions were randomly selected.

Twelve intervention and six comparison primary health care clinics were randomly sampled in Ugu District and Tshwane District (separately). Only clinics with above the median number of annual clinic visits for children under-5 years in the district were eligible for inclusion in the sampling frame. The comparison clinics served to capture any temporal changes in health worker knowledge, confidence and attitudes due to other interventions or trainings; hence a smaller sample was required in comparison versus intervention sites as the latter required more precise estimates of the intervention effect. A two-stage process was used to recruit participants. Firstly, research staff explained the study and participant inclusion and exclusion criteria to each facility manager during face-to-face on-site introductory meetings. The facility manager compiled a list of all eligible health workers involved in the care of pregnant women and children, including nurses, midwives, visiting doctors, lay counsellors, dieticians, nutritionists, facility managers and community health workers (CHWs). In the second stage, research staff approached eligible health workers and invited them to participate in the research. We aimed to recruit a manageable size of 8-10 health workers per clinic for participation in the intervention, and in the evaluation. The same staff were approached for the baseline and follow-up evaluations.

Sample size

The sample size was determined based on 80% power and alpha 0.05 to measure a 15-percentage points difference in health worker confidence in HIV and infant feeding counselling between the intervention and comparison clinics comparing baseline and follow-up. The expected effect was based on the researchers' experience and data from recent studies in South Africa with the baseline level of high confidence to counsel HIV-positive women on breastfeeding duration set at 45%.[23] It was assumed that the confidence score would remain unchanged in the comparison clinics,

For peer review only - http://bmjopen.bmj.com/site/about/guidelines.xhtml

implying a two-sample test in the post-intervention period. Clinic-level analyses were used for the sample size calculations, assuming a sampling ratio of 2:1 for the intervention clinics and a standard deviation of 15% in the mean score between clinics. Based on these assumptions, and adjusting for clustering, the sample size was determined to be 24 intervention clinics and 12 comparison clinics.[24] Within the intervention and comparison clinics, all health workers (nurses, midwives, visiting doctors, lay counsellors, dieticians, nutritionists, facility managers and community health workers (CHWs)), involved in caring for pregnant women and children were invited to participate in the study - we anticipated a mean number of health workers per participating facility to be 8-10.

Description of the intervention

We designed a participatory intervention comprising on-site mentoring through three workshops in each clinic, involving 303 selected health workers who provide care for pregnant women, breastfeeding mothers and their infants. This mentoring approach had five distinct features: 1) on-site: learning occurred in context 2) open to all cadres of health workers; 3) team-based; participants learned together; 4) content was led by self-identified gaps in knowledge and 5) activities were piloted and rooted in a theoretical framework. The intervention was delivered by the same trained facilitator (a nurse in Gauteng and nutritionist in KZN) in each intervention clinic. Each workshop lasted 1-2 hours over a 3-6-week period and had well-defined learning outcomes. The intervention has been described elsewhere.[25] In summary, our participatory intervention was guided by evidence that health workers' attitudes and practices are influenced by various factors, not just exposure to training and information.[26] We used Dee Fink's six part taxonomy as a guiding theory. This proposes that significant learning only occurs by developing foundational knowledge, applying skills, integrating ideas, developing new feelings/interests and values, and learning how to learn (encouraging the spirit of enquiry) (Figure 2).[27] Additionally, we applied the theory of planned behaviour to the intervention design (Figure 2).[28 29] This states that an individual's intention to perform a behaviour is influenced by the person's attitudes towards performing the behaviour, their beliefs about whether people who are important to them will approve of the behaviour (subjective norms), and their beliefs about how likely they are to be able to implement the behaviour successfully. According to this theory, if health workers are to provide infant feeding counselling and support in accordance with updated infant feeding guidelines to HIV-positive or negative mothers, they need to agree with the change, believe that their colleagues and other stakeholders will approve of the action, and believe in their ability to implement it successfully. The workshops were tailored to achieve these goals: Workshop 1 covered knowledge gaps reported by participants, controversial statements, and advantages of breastfeeding. Following workshop 1, a poster or cards with key messages were placed in a prominent place in the clinic. Workshop 2 comprised a progressive case study discussed in small groups. Workshop 3 involved one-to-one mentorship: each participant was observed providing infant feeding counselling or a case study was discussed if no mothers were available for counselling. The same facilitator conducted all three workshops at each clinic. In addition, a WhatsApp cell phone messaging group was established to support participants in intervention sites to facilitate sharing of concerns, tips for counselling and dealing with difficult situations. Key messages were posted on the group approximately weekly. Comparison and intervention sub-districts were exposed to routine supervision and training activities that took place at district level. The study team

For peer review only - http://bmjopen.bmj.com/site/about/guidelines.xhtml

documented that the June 2017 circular issued by the National Department of Health, informing health facilities of the change in Infant and Young Child feeding policy, was disseminated to comparison clinics as an announcement via e-mail and other electronic communication as well as during meetings or trainings. We documented that in Tshwane, 15 of the 18 clinics had received the circular; 11 via e-mail and three at a meeting. In Ugu nine of 17 clinics had received the circular; 8 received it via hand delivery and one via e-mail.

Patient and Public Involvement

Patients and the public were not involved in the design of this study, as the main population of interest were health workers.. The intervention and tool were piloted amongst a separate group of health workers to determine length, complexity of questions and level of understanding. These details are explained in our intervention paper.[25]

Data collection

Data were collected between August and December 2017 by dedicated trained non-nurse data collectors who were independent of the intervention staff. As per study design, data collection staff were not part of any intervention activities and had never been exposed to the intervention. The primary outcome measure for the study was confidence level of health workers to counsel on infant feeding, evaluated using a Likert-scale tool, developed after reviewing the WHO Breastfeeding Counselling Course, and the WHO HIV and Infant feeding counselling courses.[9 30-33].Secondary outcomes included health worker knowledge and attitude about breastfeeding counselling. A baseline assessment amongst all participating health workers in intervention and comparison sites was undertaken prior to the start of the intervention (August 2017). Health workers self-completed the assessment on study-provided electronic tablets at their workplaces. Questions covered basic demographic information, types of activities undertaken at work, knowledge, attitudes and confidence around counselling on infant feeding. Approximately 12 weeks after the baseline assessment, a follow-up assessment using the same tool was conducted amongst the same group of health workers. Health workers who were not in the clinic on the day of the follow-up assessments were included in a special catch-up assessment. Questionnaire software had built in range and skip logic and data were transferred automatically to a database held at the University of KwaZulu-Natal.

Data analysis

There were three outcomes in the study: a) 22 knowledge statements which were scored 1 if correctly answered and 0 if not; evaluation of answers were based on existing literature and guidelines (binary outcomes); b) 21 attitude questions whose responses were measured on a 5-point Likert scale - given as completely disagree (1); disagree (2); neutral (3); agree (4) and completely agree (5); positive attitudes received higher scores; and c) 19 statements on confidence item questions which were also measured on a Likert scale, scored as such: not at all confident (1), not confident (2), confident (3) and very confident (4). For both attitude and confidence domains, a participant outcome was measured by the sum of the responses to the respective items (we verified that there was not a missing response on the items). Thus, the ranges for the attitude and confidence scores were 21 to 105, and 19 to 75, respectively.

Participants baseline and follow-up characteristics and outcomes between the intervention and control areas were compared using Chi-squared tests for categorical variables and two-sample t

For peer review only - http://bmjopen.bmj.com/site/about/guidelines.xhtml

tests for continuous measures, after confirming that data were normally distributed. To assess the effect of the proposed intervention, several analysis methods for comparing intervention effect in before (pre)-after (post) quasi-experimental designs were considered. These included using post-measures and change from pre-intervention to post-intervention as the response variables. These approaches that use change and post measurements as the outcome, adjusting for pre-intervention measurements are recommended, and often give similar results.[34] In this paper, we considered three methods for estimating and testing the intervention effect using the sum of individual attitude or confidence scores as an outcome variable in a linear regression. The first method used the post-intervention measurements as the outcome variable, but adjusted for the pre-intervention values; The second method analysed the change score as an outcome variable adjusting for pre-treatment values. The third method analysed the vectors of pre-and post-measurements as the outcome variable, and used time (coded : 1 at follow-up and 0 at baseline) and treatment (coded:1 intervention group and 0: comparison group) as a covariates with an interaction term for time and treatment, in addition to an adjustment for pre-treatment values). Using methods 1 and 2 the coefficient for the intervention estimated the differences in the post intervention means and differences in the mean change of sum scores mean between the groups, controlling for the pre-intervention measurement. Using the third method, the sum of coefficients of intervention and the interaction terms was taken as the mean difference between groups post-treatment, allowing for pre-treatment mean differences between the groups. All analyses also controlled for baseline participant characteristics and prior training. Analyses adjusted for possible clustering effect at the site level, using a variance-correction method.[24] Data can be obtained by e-mailing the corresponding author.

Ethics

Ethics approval was obtained from the South African Medical Research Council (EC028-9/2016), the University of KwaZulu-Natal (RECIP348/17) and the WHO Ethics Review Committee (ERC0002833)**.** Permission for undertaking the study was obtained from Tshwane and KZN Districts. Informed consent was sought from all study participants and no personal identifying information was captured in the questionnaires, only a study identification number.

Results

At baseline and follow-up, 23 intervention clinics (one large clinic was sampled twice with two rounds of data collection per time point) and 12 comparison clinics were visited; 289 and 131 health worker interviews were conducted at baseline in intervention and comparison clinics, respectively (Figure 3). Loss to follow-up between baseline and follow-up did not differ between intervention and comparison sites: 17 (13%) in comparison sites versus 36 (12.5%) in intervention sites.

Tshwane and Ugu Districts did not differ in the main outcome measures at baseline (knowledge, attitude and confidence). Additionally, they were similar in all health worker characteristics except three:: Tshwane had significantly more participants with less than 2 years employment (14.4% versus 6.2%, p=0.007), more registered nurses (57% versus 26.2%, p=0.02), and fewer lay counsellors/CHWs (7.3% versus 50.0%, respectively, p=0.02). Given the lack of significant difference in the main outcome variables at baseline, data from the two sites were combined for the analysis.

For peer review only - http://bmjopen.bmj.com/site/about/guidelines.xhtml

All staff approached agreed to participate in the interviews. There were no significant differences between intervention and comparison sites at baseline, regarding district of origin, median age of respondent, gender, cadre of health worker, and working duration (Table 1). The proportion of participants who had received previous training (through the routine health system) on specific topics was similar in intervention versus comparison sites, except for three topics which had better coverage in comparison sites (Supplementary Figure 1). These were: ever trained on how to assess and support ART adherence for HIV positive women (78.6% in intervention sites versus 89.2% in comparison sites, p=0.01); ever trained about managing breastfeeding problems (76.5% in intervention sites and 86.2% in comparison sites, p=0.02); and received any information or training about the revised infant feeding policy (55.1% in intervention sites versus 67.4% in comparison sites p=0.02). At baseline, activities around breastfeeding counselling and management were similar between comparison and intervention sites in all respects except that comparison site participants reportedly spoke more frequently to HIV positive pregnant women about feeding than intervention participants (67% versus 71.6% spoke more than 1-3 times per month, p=0.04, data not shown).

For peer review only

For peer review only - http://bmjopen.bmj.com/site/about/guidelines.xhtml

Table 1: Characteristics of the participants in the intervention and comparison groups at baseline

Characteristic	Intervention group (n=289) (N (%))	Comparison group (n=131) (N (%))	p-value
**District:**			
- Tshwane	152 (52.6)	56 (42.8)	0.06
- Ugu	137 (47.4)	75 (57.3)	
**Age categories:**			
- 23 to 35 years	56 (19.4)	38 (29.7)	
- 36 to 41 years	61 (21.2)	25 (19.5)	
- 42 to 46 years	53 (18.4)	26 (20.3)	0.11
- 47 to 54 years	64 (22.2)	18 (14.1)	
- Over 54 years	54 (18.8)	21 (16.4)	
**Gender**			
- Female	267 (92.7)	118 (91.5)	0.66
- Male	21 (7.3)	11 (8.5)	
**Cadre of health worker**			
- Community level worker	84 (29.5)	52 (40,0)	
- Trained health professional*	151 (53.0)	64 (49.2)	0.05
- Enrolled nurse	50 (17.4)	14 (10.8)	
**Work experience in year (yr)/ years (yrs)**			
- Less than 1 yr	4 (1.4)	3 (2.3)	
- 1 to <2 yrs	23 (8.0)	12 (9.3)	
- 2 to less than 5 yrs	36 (12.5)	18 (14.0)	0.20
- 5 to less than 10 yrs	71 (24.7)	43 (33.3)	
- 10 yrs or more	154 (53.5)	53 (41.1)	

Abbreviations: *includes 68% nurses in the intervention arm and 58% nurses in comparison arm. This group also includes operation managers, dieticians, doctors and nutritionists.

In intervention sites, workshops were attended by 84-88% of participants interviewed at follow-up (Table 2).

Table 2: Attendance at workshops 1-3 measured at follow-up in intervention sites

	Attended workshop n	Attended catch-up n	Total attended n/N (%)
**Number of staff attending each workshop:**			
Group workshop 1	202	63	265/303 (87.5)
Group workshop 2	223	34	257/303 (84.8)
Workshop 3 (Clinical mentoring)	216	40	256/303 (84.5)
**Number of workshops attended:**			
	number	%	
No workshop	42	13.9	
1 workshop	8	2.6	
2 workshops	6	2.0	
All 3 workshops	247	81.5	
**Total**	**303**	**100**	

For peer review only

Effect of the intervention on health worker knowledge

At baseline, knowledge about key infant feeding statements or facts was similar between intervention and comparison sites, except for knowledge about soft porridge (Table 3). Although at baseline, more than 90% of intervention and comparison site participants knew that a baby under 4 months should not be given soft porridge if hungry, significantly more intervention site participants knew this recommendation (Table 3). The percentage of participants at baseline correctly answering the more difficult questions (on bottle sterilisation, storing expressed breastmilk, feeding HIV exposed infants) was low (Table 3). At follow-up significantly more intervention site participants correctly answered knowledge questions, regarding the leading cause of death in children under 5, the risk of formula feeding, duration of breastfeeding for HIV-negative mothers and women living with HIV, how to stop breastfeeding, complementary feeding, storing expressed breastmilk, feeding whilst at work, breastfeeding and viral suppression, mixed feeding in women living with HIV, adherence to ART and breastfeeding, breastfeeding difficulties in women living with HIV and managing women living with HIV who are breastfeeding, than comparison site participants (Table 3). The significant differences between intervention and comparison sites regarding soft porridge were not present at follow-up. Although improvements were seen in knowledge related to the risks of mixed feeding for women living with HIV, most health workers still provided incorrect responses at follow-up. At baseline, the mean number of correctly answered knowledge questions was 15.0 (68%) in comparison sites versus 15.2 (69%) in intervention sites, p=0.89 (Table 3). At follow-up the mean number was to 15.2 (69%) in comparison sites and 17.2 (78.2%) in intervention sites, p<0.001 (Table 3). For the two questions measuring knowledge about the 2017 change in infant feeding guidelines, namely, "Continued breastfeeding for 2 years is the recommended infant method in SA for ALL children, regardless of mother's HIV status" and "In South Africa, HIV-infected women who are breastfeeding should be supported to adhere to antiretroviral treatment and should introduce complementary foods at 6 months and be supported to continue breastfeeding for at least two years. (True)", there was a 36% improvement in knowledge in the intervention group at follow-up compared with a 13% increase in knowledge in the control group. For the second question there was a 15% increase in correct knowledge in the intervention group at follow-up while for the comparison group knowledge decreased from 89-81%. At follow-up, knowledge scores of participants who attended 3 workshops compared with knowledge scores of participants who attended less than 3 workshops was significantly better (p<0.001).

For peer review only - http://bmjopen.bmj.com/site/about/guidelines.xhtml

Table 3: Knowledge of health workers about breastfeeding in the intervention and comparison sites at baseline and follow-up

Knowledge statements	Number (%) with correct answers at BASELINE			Number (%) with correct answers at FOLLOW-UP		
	Intervention (n=289)	Comparison (n=131)	p-value *	Intervention (n=250)	Comparison (n=112)	p-value *
**Knowledge relating to updates in the HIV and Infant feeding guidelines**						
Significant improvements between intervention and comparison groups at follow-up						
Continued breastfeeding for 2 years is the recommended infant method in SA for ALL children, regardless of mother's HIV status (True)ǂ	190 (65.7)	91 (70.0)	0.39	224 (89.6)	88 (78.6)	<0.01
An HIV-positive mother who is virally suppressed on antiretroviral treatment should breastfeed her child rather than not breastfeed to improve the child's survival (True) ǂ	237 (82.0)	108 (83.1)	0.79	236 (94.4)	96 (85.7)	<0.01
A mother who has missed 6 tablets of Fixed Dose Combination ART in one month is considered to be poorly adherent and should stop breastfeeding immediately (False)** ǂ	181 (62.6)	89 (68.5)	0.25	201 (80.4)	72 (64.3)	<0.01
In South Africa, HIV-infected women who are breastfeeding should be supported to adhere to antiretroviral treatment and should introduce complementary foods at 6 months and be supported to continue breastfeeding for at least two years. (True) ǂ	245 (84.8)	116 (89.2)	0.220	244 (97.6)	91 (81.3)	<0.01
When an HIV-infected mother is ready to add complementary feeds she should stop breastfeeding rapidly over a 24-hour period (False) ǂ	214 (74.1)	103 (79.2)	0.25	217 (86.8)	86 (76.8)	<0.05
If a mother misses 2 doses of her ART in one month, she should be classified as a treatment failure (False)** ǂ	185 (64.0)	82 (63.1)	0.85	191 (76.4)	73 (65.2)	<0.05
Low levels of knowledge (<80%) at baseline in both groups - – no significant differences between intervention and comparison groups at follow-up [concept that this relates to]						
If an HIV exposed baby is receiving both breastmilk and formula milk, the mother should choose either breastfeeding or formula feeding if she is adherent to ART (False) ǂ [mixed feeding with formula and breastmilk]	69 (23.9)	29 (22.3)	0.71	75 (30.0)	28 (25.0)	0.33
A mother living with HIV and adherent to antiretroviral treatment cannot exclusively breastfeed her 4-month old infant because she is working. It is better for this mother to give formula during the day and breastfeed at night rather than giving no breast milk at all (True) ǂ [mixed feeding with formula and breastmilk]	22 (7.6)	14 (10.8)	0.29	40 (16.0)	14 (12.5)	0.38

For peer review only

High levels of knowledge (≥80%) at baseline in both groups – no significant differences between intervention and comparison groups at follow-up						
In South Africa, HIV-infected women who are breastfeeding should be supported to adhere to antiretroviral treatment and should be counselled and supported to exclusively breastfeed their infants for the first six months of life whilst maintaining an undetectable viral load (True)ǂ	281 (97.2)	123 (94.6)	0.18	242 (96.8)	109 (97.3)	0.79
Mothers living with HIV who are receiving antiretroviral treatment and are virally suppressed should be advised not to breastfeed their infants (False) ǂ	252 (87.2)	115 (88.5)	0.85	230 (92.0)	99 (88.4)	0.27
**General breastfeeding**						
Significant improvements between intervention and comparison groups at follow-up						
In South Africa, the leading cause of death amongst children under 5 is pneumonia (True)	189 (65.4)	82 (63.1)	0.65	230 (92.0)	75 (67.0)	<0.01
Giving any formula milk during the first six months of life increases the risk of death from diarrhoea and/or pneumonia (True)	246 (85.1)	104 (80.0)	0.17	232 (92.8)	95 (84.8)	0.02
It is safe to give the baby expressed breastmilk that has been kept outside the fridge for 8 hours (True)	106 (36.7)	43 (33.1)	0.48	120 (48.0)	38 (33.9)	<0.05
A mother who is working and giving formula milk should mix the milk herself and leave for the carer to give during the day (False)	218 (75.4)	94 (72.3)	0.50	189 (75.6)	68 (60.7)	<0.01
Low levels of knowledge (<80%) at baseline in both groups - – no significant differences between intervention and comparison groups at follow-up						
When sterilising feeding bottles cover the bottles with water in a saucepan and place on the heat. As soon as the water boils remove from heat and do not leave the bottles in the water until completely cool (False)	64 (22.2)	27 (20.8)	0.75	53 (21.2)	25 (22.3)	0.81
High levels of knowledge (≥80%) at baseline in both groups – no significant differences between intervention and comparison groups at follow-up						
Exclusive breastfeeding is the recommended infant feeding method for ALL infants aged 0-6 months in SA, regardless of mother's HIV status (True)	271 (93.8)	118 (90.8)	0.27	234 (93.6)	102 (91.1)	0.32
A baby under 4 months should be given soft porridge once he/she seems hungry (False)	284 (98.3)	124 (95.4)	0.09	247 (98.8)	108 (96.4)	0.13
Giving a baby expressed breastmilk is not as good as breastfeeding (False)	234 (81.0)	106 (81.5)	0.89	218 (87.2)	96 (85.7)	0.70
There are long term health benefits of breastfeeding for mother and child that last beyond the breastfeeding period (True)	264 (91.4)	116 (89.2)	0.49	232 (92.8)	100 (89.3)	0.26

For peer review only - http://bmjopen.bmj.com/site/about/guidelines.xhtml

**Breastfeeding and HIV**						
Significant improvements between intervention and comparison groups at follow-up						
An HIV-positive mother who has cracked nipples should continue to breastfeed unless they are bleeding (True)	143 (49.5)	64 (49.2)	0.96	187 (74.8)	59 (52.7)	<0.01
If a baby has a positive PCR (HIV test) at birth the mother should stop breastfeeding if this is affordable and feasible in her situation (False)**	224 (81.0)	100 (76.9)	0.90	214 (85.6)	82 (73.2)	<0.01
High levels of knowledge (<80%) at baseline in both groups – no significant differences between intervention and comparison groups at follow-up						
An HIV-exposed baby who is exclusively breastfeeding should be given some water when the weather is very hot (False)**	270 (93.4)	122 (93.9)	0.87	239 (95.6)	105 (93.8)	0.45
**Mean knowledge score (standard deviation) out of 22**	15.2 (2.6)	15.0 (3.1)	0.61*	17.2 (2.1)	15.2 (2.8)	<0.01

**The Statement is false; thus, the scales were inverted during data analysis. ǂ - these questions measure the change in knowledge relating to the South African Department of Health June 2017 circular and the WHO 2016 updated HIV and Infant feeding guidelines

*independent t-test comparing intervention and comparison sites at the relevant time point.

Note: the tables displays numbers with correct knowledge

Effect of the intervention on attitudes

At baseline, intervention and comparison sites were similar in HW attitudes except for attitudes towards feeding a crying baby and expressing breastmilk, which were significantly better in intervention sites (Supplementary Table 1). At follow-up attitudes to breastfeeding and HIV were significantly better in the intervention group for 13 of the 21 questions and the mean attitude score towards breastfeeding was significantly better in intervention sites (p<0.001) (Supplementary Table 1). At follow-up HW in the intervention group were significantly less confused about what to tell women living with HIV about infant feeding mothers about HIV and infant feeding. All three analysis methods demonstrated that, after controlling for other variables, final attitude (measured as attitude at follow-up, change in attitude between intervention and comparison sites or change in attitude between baseline and follow-up between intervention and comparison sites) was significantly better in intervention compared with comparison sites (Table 4). The first two methods yielded almost exactly the same results. In methods 1 and 2, attitude at follow-up was 5.4 points higher in the intervention group than the comparison group; Method 3 analysis showed a significant 5.1-point higher score in the intervention compared with the comparison group Using method 1, being an enrolled nurse, and being in the youngest (36-41 years) or oldest (>54 years) age group was associated with a significantly lower attitude score. Results from application of methods 3 show that trained health professionals had a significantly higher attitude score at follow-up (Table 4, p<0.05). We did not detect a dose-association in intervention sites when comparing 0-1 or 1-2 versus 3 workshops (p=0.4); but numbers in each group may have been too small to reliably assess any dose effect.

For peer review only - http://bmjopen.bmj.com/site/about/guidelines.xhtml

Table 4: Adjusted effect of the intervention on health worker attitude score using different methods (Effect estimate and 95% confidence interval (CI))

Variable	Method 1		Method 3	
	Effect estimate	95% CI	Effect estimate	95% CI
**Attitude score at baseline**	**0.5**	**0.3; 0.7***	N/A	N/A
**Intervention**	**5.4**	**3.9; 6.9***	**5.1**	**2.1; 8.1***
**Follow-up Period**	N/A	N/A	1.8	0.2-3.4*
**Professional role: vs community level**				
- Trained health professional	1.6	-0.05; 3.2	**4.8**	**2.8; 6.7***
- Enrolled nurse	**-2.4**	**-5.0; -0.2***	0.9	-1.4; 3.2
**Ugu District vs Tshwane District**	-0.83	-2.2; 0.5	-1.4	-3.1; 0.2
**Age category: vs 23-35 yrs**				
- 36 to 41 yrs	**-2.8**	**-5.4; -0.2***	-1.8	-4.1; 0.6
- 42 to 46 yrs	-0.9	-3.3; 1.5	-0.2	-2.5; 2.2
- 47 to 54 yrs	0.5	-2.0; 2.9	-1.2	-3.1; 0.8
- over 54 yrs	**-3.3**	**-5.7; -1.0***	-2.2	-4.8; 0.3
**Work experience <5 yrs vs ≥5yrs**	-0.3	-2.5; 1.9	-1.3	-3.4; 0.8
**Received training or information at work about the revised policy**	0.5	-1.4; 2.3	1.7	0.1-3.24
**Received any training about managing common breastfeeding problems?**	0.3	-2.5; 3.0	3.2	0.9-5.5
**Ever received any training about how to assess and support ART adherence for HIV positive women?**	-0.1	-2.2; 2.0	1.6	-0.5-3.8

*p<0.05 N/A: not applicable. Note: All analyses are adjusted for clustering

Effect of the intervention on confidence

At baseline there was no difference in the percentage of participants in the intervention and control sites who were confident or very confident in counselling mothers on HIV / infant feeding (Supplementary Table 2). However, at follow-up HWs from intervention sites were significantly more confident in counselling an HIV positive women about HIV and infant feeding, advising HIV positive women about return to school/work, advising HIV positive mothers to continue breastfeeding for two years, assessing ART adherence in HIV positive mothers, and advising HIV positive mothers about breastfeeding with cracked nipples (Supplementary table 2). Confidence had not shifted about how to stop breastfeeding, identifying when a mother is not ART adherent and managing poor adherence, advising on formula feeding and counselling that a shorter breastfeeding duration is better than no breastfeeding. The mean confidence score at follow-up was significantly higher in the intervention compared with the comparison sites at follow-up (p=0.05) (Supplementary Table 2). All three analysis methods demonstrated that confidence was significantly better in intervention compared with comparison sites (Table 5). Analysis based on method 1 (Table 5), showed that confidence at follow-up was 2.4 points higher in the intervention arm than the comparison arm. In However, under method 3 (Table 5), the estimate of the effect

For peer review only - http://bmjopen.bmj.com/site/about/guidelines.xhtml

was not significant, thought it was 1.5 point higher score in the intervention compared with the comparison group, (Table 5). Our analysis demonstrated that, controlling for other factors, being a trained health professional significantly increased confidence score by 3.1 (Method 1) or 3.7 (Method 3). Additionally, Methods 3 demonstrated that, controlling for other factors, working for less than 5 years significantly reduced the confidence score. We did not measure a dose-effect (one or two versus three workshops (p=0.4)); but numbers in each group may have been too small to assess this.

Table 5: Adjusted effect of the intervention on health worker confidence scores, using different multivariable analysis methods (Effect estimate and 95% confidence interval (CI))

Variable	Method 1		Method 3	
	Effect estimate	95% CI	Effect estimate	95% CI
**Confidence score at baseline**	**0.4**	**0.3; 0.6***	N/A	N/A
**Intervention**	**2.4**	**0.3; 4.5***	1.5	-2.2; 5.1
**Follow-up time**	N/A	N/A	0.5	-1.5; 2.5
**Cadre of heath professional: vs community level**				
- Trained health professional	**3.1**	**0.3; 5.9***	**3.7**	**1.5; 5.9***
- Enrolled nurse	-0.8	-4.3; 2.7	-0.7	-3.1; 1.6
**Ugu District vs Tshwane District**	0.00	-2.1; 2.1	-1.	-3.2; 1.2
**Age category vs 23-35 yrs**				
- 36 to 41 yrs	-1.0	-3.7; 1.6	-0.1	-2.7; 2.5
- 42 to 46 yrs	0.3	-2.9; 3.4	0.4	-1.2; 2.9
- 47 to 54 yrs	1.4	-0.7; 3.5	-1.3	-3.4; 0.8
- over 54 yrs	-2.5	-5.7; 0.7	-0.9	-4.0; 2.2
**Work experience <5 yrs vs ≥5 yrs**	-0.5	-3.4; 2.4	**-1.9**	**-3.7; -0.2***
**Received training or information at work about the revised policy**	0.05	-1.5; 1.6	1.7	-0.3; 3.6
**Received any training about managing common breastfeeding problems?**	-0.6	-3.2; 2.1	1.8	-0.5; 4.1
**Ever received any training about how to assess and support ART adherence for HIV positive women?**	0.8	-2.1; 3.7	5.7	3.5; 7.9

*p<0.005 N/A= not applicable yrs= years. NB: All analyses are adjusted for clustering

Discussion

We demonstrate that a participatory, side-by side, team-based mentoring approach to disseminating updated HIV and infant feeding guidelines was associated with an improvement in health workers' attitudes. when controlling for other factors. There was also a significant improvement in mean knowledge score between intervention and control sites at follow-up. However, we were not successful in shifting knowledge and attitudes about mixed feeding

For peer review only - http://bmjopen.bmj.com/site/about/guidelines.xhtml

(breastmilk and formula milk) and health workers at the end of the study were not confident in advising that a shorter duration of breastfeeding is better than no breastfeeding at all. This demonstrates the success of at least 15 years of frequent publicity about the dangers of mixed feeding in the context of HIV and no ART, given that the two seminal papers on feeding practices and HIV were led by South African researchers.[35 36] A new pervasive broadcast highlighting the acceptability of mixed feeding in the context of ART and maternal viral load suppression is needed to facilitate a shift in knowledge about mixed feeding. Although some individual attitude and confidence items did not change, or only changed marginally, the overall analyses demonstrated an improvement in follow-up attitude and confidence scores. However, confidence in the intervention group was still low and health workers performed poorly on some of the more difficult confidence questions such as confidence with counselling when a mother is not ART adherent, managing high viral loads during breastfeeding, explaining HIV transmission risks to a mother with a high viral load, assisting mothers with HIV to safely formula feed and advising that some breastfeeding is better than no breastfeeding. The complexity of changing health workers' attitudes and confidence towards breastfeeding has been documented repeatedly in many African settings including South Africa.[11 25 37-40] We hypothesise that poor performance on some of the individual items or on the overall confidence score may be attributed to the short duration of the intervention. An alternative hypothesis is that HW's low confidence, around topics like non-adherence and high viral load, reflect more complex dynamics that are not easily addressed through counselling / mentoring interventions.[38] In fact a study from South Africa demonstrated how health workers personal beliefs affect their ability to provide supportive counselling.[11]

There is evidence that in-service training, supervision and follow-up improves the knowledge, skills and practices of health workers managing childhood undernutrition, and can improve health worker job satisfaction and motivation, but no data exist on how to improve health worker knowledge, skills and confidence in the tricky area of HIV and infant feeding.[41-43] For training / supervision interventions, implementation challenges include inadequately trained or shortages of supervisors, inappropriate job aids for follow-up, and poor alignment between community views/ practice and health programmes.[44] Our approach attempted to circumvent these challenges by using a low technology, mentorship model for skills development at clinic level. At the outset of the intervention we acknowledged that health workers were members of their community: we discussed their fears and beliefs, and then introduced facts and evidence to extend their knowledge, change their attitudes and increase their confidence to implement updated guidelines on HIV and infant feeding. Thus, we aimed to change inherent, deep seated beliefs and attitudes that are sustained in the absence of outside supervision.

We used a side-by-side mentorship approach, as reviewed by Schwerdte et.al. to conduct team-based mentoring to empower health workers.[45] A team-based approach allowed collaborative learning between different cadres of health workers, facilitating any future change in practice. In accordance with Dee Fink's theory, a participatory mentorship approach allows participants to develop foundational knowledge, apply skills, integrate ideas, develop new feelings/ interests and values and learn how to learn.[26] Our experience suggests that such an approach allowed discussion of participant's attitudes towards performing the behaviour, beliefs about whether critical, important people will approve of the behaviour (subjective norms), and about their likelihood of successfully implementing the behaviour.[28 29] Our findings corroborate a scoping

For peer review only - http://bmjopen.bmj.com/site/about/guidelines.xhtml

review which demonstrated that mentorship improves certain quality of care outcomes [45]; in our study it improved knowledge, attitudes and confidence. However, only four studies were included in this scoping review, and the nature of the mentorship varied from video-conferencing to monthly, six-weekly or annual visits interspersed with other contact forums, conducted over one day to an entire week. A list of desirable features of mentorship interventions, include at least one dedicated mentor per facility, ensuring an adequate mentor:mentee ratio so that all staff can be supported, forming meaningful relationships between mentors and mentees, ensuring cultural congruency between mentee and mentor, and using mentors for protocol-driven programmes, such as IMCI or HIV.[45] Our intervention related to HIV and infant feeding guidelines, was low cost and low technology (one mentor working with pen, flip chart and paper in the health facility), and was implemented by a dedicated mentor from the same cultural background as the mentees. She provided onsite support during the workshops, which lasted approximately one hour, and additional support through a WhatsApp messaging group.

There is an ongoing heath worker crisis in resource limited settings, including maldistribution of staff, an imbalance in skills mix, increasingly complicated health programmes and complicated socio-cultural-political-economic environments. Against this background, questions arise about the feasibility of an on-site mentorship approach to guideline dissemination amongst health workers, and an on-site peer-peer mentorship approach to supporting mothers with infant feeding. In this study we chose to focus specifically on an onsite mentorship approach to guideline dissemination amongst health workers. We argue that strengthening investment in on-site mentorship rather than off-site training, may be a cost-saving approach:. In our setting, all clinics receive regular visits from district primary health care (PHC) supervisors, but they mostly focus on administration and clinic management matters. These supervisors, as well as existing district PHC trainers, could be capacitated to provide clinical mentoring for health workers in the clinics they oversee. Our model of team-based learning and mentoring can be used for on-site mentoring, and avoids accommodation and travel costs, and absence from work that off-site training requires.

Our study had several limitations: We purposively selected districts for inclusion. We could not control for previous breastfeeding experience of health workers as we did not gather these data. The study tools were piloted before finalisation, but no factor analyses or validation exercises were conducted. The follow-up evaluation was conducted 3 months after the intervention; thus, we were only able to measure short term benefits. Additionally, we did not co-design the intervention with women living with HIV, did not measure the effect of improved knowledge, attitudes and confidence on health workers' counselling practices and on mothers' infant feeding practices and could not tease out whether the relationship between number of workshops and outcomes was due to staff motivation (more motivated staff attended more workshops) or the workshops themselves. Our study's strengths are that the design was quasi-experimental, measuring not only knowledge, but also attitudes and confidence. Additionally, results are robust as three different analytical methods yielded congruent results.

Conclusion: We demonstrated improved knowledge, attitudes and confidence of health workers following a participatory mentorship approach to HIV and infant feeding guideline dissemination compared with a standard approach. More research is needed to better understand how to change health workers' counselling practices, and whether this changes mothers' feeding practices.

For peer review only - http://bmjopen.bmj.com/site/about/guidelines.xhtml

Authors contributions:

AG: Study conceptualization and tool development, protocol writing including intervention development, oversight of sampling and field work, writing of the first draft of this manuscript, receiving and incorporating co-author comments, finalization of the paper
TD: Study conceptualization and tool development, protocol writing including intervention development, set up the sample frame and sampling, contributed to the manuscript, reviewed and approved the final version of the manuscript
SM: Led the statistical components of the protocol; provided overall oversight on the statistical analysis, contributed to the manuscript, reviewed and approved the final version of the manuscript
TN: Performed the work on the statistical components of the protocol, under SM's guidance; provided data analysis under SM's guidance, contributed to the manuscript, reviewed and approved the final version of the manuscript
LH: Contributed to study conceptualization and tool development, protocol writing including intervention development; was overall Project Manager; established, managed and cleaned the database; contributed to the manuscript, reviewed and approved the final version of the manuscript
VJ: Provided guidance on intervention development. Contributed to the manuscript, reviewed and approved the final version of the manuscript
IMSE: Contributed to study conceptualization and tool development, protocol writing including intervention development; contributed to the manuscript, reviewed and approved the final version of the manuscript
UF: Contributed to study conceptualization, assisted with district level buy-in in Tshwane District, provided information on how routine dissemination of updated infant feeding guidelines; contributed to the manuscript, reviewed and approved the final version of the manuscript
AD: Contributed to study conceptualization and tool development; contributed to the manuscript, reviewed and approved the final version of the manuscript
NR: Contributed to study conceptualization; contributed to the manuscript, reviewed and approved the final version of the manuscript
MK: Contributed to study conceptualization and tool development; contributed to the manuscript, reviewed and approved the final version of the manuscript
DS: Contributed to study conceptualization and tool development; contributed to the manuscript, reviewed and approved the final version of the manuscript
SK: Contributed to study conceptualization, assisted with national level buy-in, provided information on how routine dissemination of updated infant feeding guidelines; contributed to the manuscript, reviewed and approved the final version of the manuscript
TT: Contributed to study conceptualization and tool development; contributed to the manuscript, reviewed and approved the final version of the manuscript
CH: Study conceptualization, protocol writing including intervention development, high level oversight of study implementation, contributed to the manuscript, reviewed and approved the final version of the manuscript

Data sharing statement: Data can be obtained by e-mailing the corresponding author, and upon reasonable request

For peer review only - http://bmjopen.bmj.com/site/about/guidelines.xhtml

Figure legends:

Figure 1: Study districts: Tshwane District in Gauteng Province and Ugu District in KwaZulu-Natal Province of South Africa

Figure 2: Theoretical frameworks which informed the development of the intervention

Figure 3: Study population at baseline and follow-up for intervention and comparison sites

Supplementary Figure 1: Prior training reported by participants at baseline (intervention versus comparison groups)

For peer review only

For peer review only - http://bmjopen.bmj.com/site/about/guidelines.xhtml

References

1. Victora C, Bahl R, Barros A, et al. Breastfeeding in the 21st century: epidemiology, mechanisms, and lifelong effect. The Lancet 2016;**387**(10017):475-90
2. Black RE, Allen LH, Bhutta ZA, et al. Maternal and child undernutrition: global and regional exposures and health consequences. Lancet. 2008.;**37**(9608):243-60
3. National Department of Health. Policy and Guidelines for the Implementation of the PMTCT programme. 2008. Available from http://www.info.gov.za/view/DownloadFileAction?id=77877. Accessed 11 June 2020.
4. National Department of Health. The South African Antiretroviral Treatment Guidelines 2013: PMTCT Guidelines Revised March 2013. 2013. Available from https://sahivsoc.org/Files/2013%20ART%20Treatment%20Guidelines%20Final%2025%20March%202013%20corrected.pdf. Accessed 11 June 2020.
5. National Department of Health. National conslidated guidelines for the prevention of mother to child transmission of HIV (PMTCT) and the management of HIV in children, adolescents and adults. 2015. Available from https://www.health-e.org.za/2015/07/02/guidelines-national-consolidated-guidelines-for-pmtct-and-the-management-of-hiv-in-children-adolescents-and-adults/. Accessed 11 June 2020.
6. National Department of Health. Guideline for the Prevention of Mother to Child Transmission of Communicable Infections. 2019. Available from https://www.knowledgehub.org.za/system/files/elibdownloads/2019-10/PMTCT%20Guideline%2028%20October%20signed.pdf. Accessed 11 June 2020.
7. National Department of Health and SANAC. Clinical Guidelines: PMTCT (Prevention of Mother-to-Child Transmission). 2010. Available from https://www.fidssa.co.za/Content/Documents/PMTCT_Guidelines.pdf. Accessed 11 June 2020.
8. National Department of Health Breastfeeding Summit. The Tshwane Declaration of Support for breastfeeding in South Africa. S Afr J Clin Nutr 2011;**24**(4):214-16
9. World Health Organisation, UNICEF. 2016 Guideline Update on HIV and Infant Feeding. 2016. Available from https://www.who.int/nutrition/publications/hivaids/guideline_hiv_infantfeeding_2016/en. Accessed 11 June 2020.
10. Martin-Wiesnerm, P. A policy-friendly environment for breastfeeding A review of South Africa's progress in systematising its international and national responsibilities to protect, promote and support breastfeeding. In: University of Witwatersrand: DST-NRF Centre of Excellent in Human Development, 2018. Available from https://www.wits.ac.za/media/wits-university/research/coe-human/documents/Breastfeeding%20policy%20review.pdf. Accessed 11 June 2020..
11. Nieuwoudt S, Manderson L. Frontline health workers and exclusive breastfeeding guidelines in an HIV endemic South African community: a qualitative exploration of policy translation. International Breastfeeding Journal (2018) 13:20 2018;**13**(20):https://doi.org/10.1186/s13006-018-0164-y
12. Doherty T, Chopra M, Nkonki L, Jackson d, Greiner T. Effect of the HIV epidemic on infant feeding in South Africa: "When they see me coming with the tins they laugh at me". Bull of the World Health Organ. 2006;**84**(2):90-96

For peer review only - http://bmjopen.bmj.com/site/about/guidelines.xhtml

13. Doherty T, Horwood C, Haskins L, et al. Breastfeeding advice for reality: Women's perspectives on primary care support in South Africa. . Maternal & Child Nutrition 2019;**16**(1):e12877 DOI: 10.1111/mcn.77.
14. Seidel G, Sewpaul V, Dano B. Experiences of breastfeeding and vulnerability among a group of HIV-positive women in Durban, South Africa. Health Policy Plan 2000;**15**:24-33
15. McCormack L, Sheridan S, Lewis M, et al. Communication and dissemination strategies to facilitate the use of health-related evidence. Secondary Communication and dissemination strategies to facilitate the use of health-related evidence. 2013. https://www.ncbi.nlm.nih.gov/books/NBK179104/pdf/Bookshelf_NBK179104.pdf.
16. Academy of Nutrition and Dietetics, Handu D, Acosta A, et al. Effectiveness of interventions to promote exclusive breastfeeding in women living with HIV that are on antiretroviral therapy living in areas that promote exclusive breastfeeding due to limited resources for safe replacement feeding. 2016. Available from https://www.who.int/maternal_child_adolescent/documents/hiv-infant-feeding-2016-ax2-breastfeeding-promotion_systematic-review.pdf?ua=1. Accessed 10 June 2020.
17. World Health Organization, United Nations Children's Fund. Guideline: updates on HIV and infant feeding: the duration of breastfeeding, and support from health services to improve feeding practices among mothers living with HIV. Geneva, 2016. Available from http://apps.who.int/iris/bitstream/handle/10665/246260/9789241549707-eng.pdf;sequence=1. Accessed 9 June 2020.
18. Barker M, Baird J, Lawrence W, et al. The Southampton Initiative for Health: a complex intervention to improve the diets and increase the physical activity levels of women from disadvantaged communities. . J Health Psychol 2011;**16**:178-91
19. Baird J, Jarman M, Lawrence W, al. e. The effect of a behaviour change intervention on the diets and physical activity levels of women attending Sure Start Children's Centres: results from a complex public health intervention. . BMJ Open 2014;**4:e005290.**
20. Heerink F, Krumeich A, Feron F, A. G. We are the advocates for the babies' - understanding interactions between patients and health care providers during the prevention of mother-to-child transmission of HIV in South Africa: a qualitative study. . Global Health Action 2019.;**12**:DOI: 10.1080/16549716.2019.1630100
21. Hector D, Webb K, King L. Factors affecting breastfeeding practices. Applying a conceptual framework. New South Wales Public Health Bulletin 2005:https://www.researchgate.net/profile/Lesley_King2/publication/7654972_Factors_affecting_breastfeeding_practices_Applying_a_conceptual_framework/links/004635254b6bd3b8d7000000/Factors-affecting-breastfeeding-practices-Applying-a-conceptual-framework.pdf?origin=publication_detail
22. Nieuwoudt S, Ngandu C, Norris S. Exclusive breastfeeding policy, practice and influences in South Africa, 1980 to 2018: A mixed-methods systematic review. PLoS One. 2019; 14(10): e0224029. 2019;**14**(10):doi: 10.1371/journal.pone.0224029
23. Janse van Rensburg L, Nel R, Walsh CM. Knowledge, opinions and practices of healthcare workers related to infant feeding in the context of HIV. Health SA Gesondheid 2016;**21**:129-36 doi: http://dx.doi.org/10.1016/j.hsag.2015.12.001[published Online First: Epub Date]|.
24. Eldridge S, Ashby D, Kerry S. Sample size for cluster randomized trials: effect of coefficient of variation of cluster size and analysis method. International Journal of Epidemiology 2006;**35**(1292-1300):doi:10.1093/ije/dyl129

For peer review only - http://bmjopen.bmj.com/site/about/guidelines.xhtml

25. Horwood C, Haskins L, Goga A, et al. An educational intervention to update health workers about HIV and infant feeding. . Maternal & Child Nutrition. 2019:https://doi.org/10.1111/mcn.12922
26. Pariyo GW, Gouws E, Bryce J, Burnham for the Uganda IMCI Impact Study Team,. Improving facility-based care for sick children in Uganda: training is not enough. Health Policy Plan 2005;**20**(Suppl_1):i58-i68
27. Dee Fink L. A self-directed guide to designing courses for significant learning. Secondary A self-directed guide to designing courses for significant learning 2003. https://www.deefinkandassociates.com/GuidetoCourseDesignAug05.pdf.
28. Ajzen I. The theory of planned behavior. Organizational Behavior and Human Decision Processes 1991;**50**(2):179-211 doi: http://dx.doi.org/10.1016/0749-5978(91)90020-T[published Online First: Epub Date]|.
29. Ajzen I. Perceived Behavioral Control, Self-Efficacy, Locus of Control, and the Theory of Planned Behavior1. Journal of Applied Social Psychology 2002;**32**(4):665-83 doi: 10.1111/j.1559-1816.2002.tb00236.x[published Online First: Epub Date]|.
30. World Health Organization, UNICEF. Breastfeeding Counselling: A Training Course. Geneva, 1993. Available from https://www.who.int/maternal_child_adolescent/documents/who_cdr_93_3/en/. Accessed 10 June 2020.
31. World Healh Organization, UNICEF. HIV and Infant Feeding Counselling: A Training Course. Geneva, 2000. Available from https://apps.who.int/iris/bitstream/handle/10665/66194/WHO_FCH_CAH_00.4.pdf;sequence=5. Accessed 11 June 2020
32. Doherty T, Sanders D, Goga A, Jackson D. Implications of the new WHO Guidelines on HIV and infant feeding for child survival in South Africa. . Bull World Health Organ. 2011;**89**:62-67
33. Goga A, Doherty T, Jackson D, et al. Infant feeding practices at routine PMTCT sites, South Africa: Results of a prospective observational study amongst HIV exposed and unexposed infants - birth to 9 months. Int Breastfeeding J. 2012;**7**(4):doi:10.1186/746-4358-7-4.
34. O'Connell N, Dai L, Jiang Y, Speiser J, Ward R, et al. Methods for Analysis of Pre-Post Data in Clinical Research: A Comparison of Five Common Methods. . J Biom Biostat 2017;**8**(334):doi:10.4172/2155-6180.1000334
35. Coovadia H, Rollins N, Bland R, et a. Mother-to-child transmission of HIV-1 infection during exclusive breastfeeding in the first 6 months of life: an intervention cohort study. . Lancet 2007;**369**:1107-16
36. Coutsoudis A, Pillay K, Kuhn L, et a. Method of feeding and transmission of HIV-1 from mothers to children by 15 months of age: prospective cohort study from Durban, South Africa. . AIDS 2001;**15**:379-87
37. Desclaux A, Alfieri C. Counseling and choosing between infant-feeding options: overall limits and local interpretations by health care providers and women living with HIV in resource-poor countries (Burkina Faso, Cambodia, Cameroon). . Soc Sci Med. 2009;69:821–9. 2009;**69**:821-9
38. Tuthill E, Chan J, Butler L. Challenges faced by healthcare providers offering infant feeding counseling to HIV-positive women in sub-Saharan Africa: A review of current research. AIDS Care. 2015;**27**(1):17-24

For peer review only - http://bmjopen.bmj.com/site/about/guidelines.xhtml

39. Buskens I, Jaffe A. Demotivating infant feeding counselling encounters in southern Africa: do counsellors need more or different training? . AIDS Care. 2008;20(3):337–45. 2008;**20**(3):337-45
40. Nieuwoudt S, Manderson L. Frontline health workers and exclusive breastfeeding guidelines in an HIV endemic South African community: a qualitative exploration of policy translation International Breastfeeding Journal 2018;**13**(20):https://doi.org/10.1186/s13006-018-0164-y
41. Sunguya B, Poudel K, Mlunde L, Urassa D, Yasuoka J, Jimba M. Nutrition Training Improves Health Workers' Nutrition Knowledge and Competence to Manage Child Undernutrition: A Systematic Review. Frontiers in public health 2013:10.3389/fpubh.2013.00037
42. Bailey C, Blake C, Schriver M, Cubaka V, Thomas T, Martin Hilber A. A systematic review of supportive supervision as a strategy to improve primary healthcare services in Sub-Saharan Africa. Int J Gynaecol Obstet. 2016;**132**(1):117-25. doi: 10.1016/j.ijgo.2015.10.004.
43. World Health Organisation. Management of Childhood Illness in Developing Countries: Rationale for an integrated approach. 1999. Available from http://www.who.int/child_adolescent_health/documents/chs_cah_98_1a/en/. Accessed 11 June 2020.
44. Goga A, Muhe L. Global Challenges with Scale-Up of the Integrated Management of Childhood Illness Strategy: Results of a multi-country Survey. . BMC Public Health. 2011;**11**(503):doi: 10.1186/471-2458-11-503
45. Schwerdte P, Morphet, Hall H. A scoping review of mentorship of health personnel to improve the quality of health care i low and middle-income countries. Globalisation and Health 2017;**13**(77):doi 10.1186/s12992-017-0301-1

For peer review only - http://bmjopen.bmj.com/site/about/guidelines.xhtml

Figure 1: Study districts: Tshwane Metropolitan Municipality in Gauteng Province and Ugu District in KwaZulu-Natal Province of South Africa

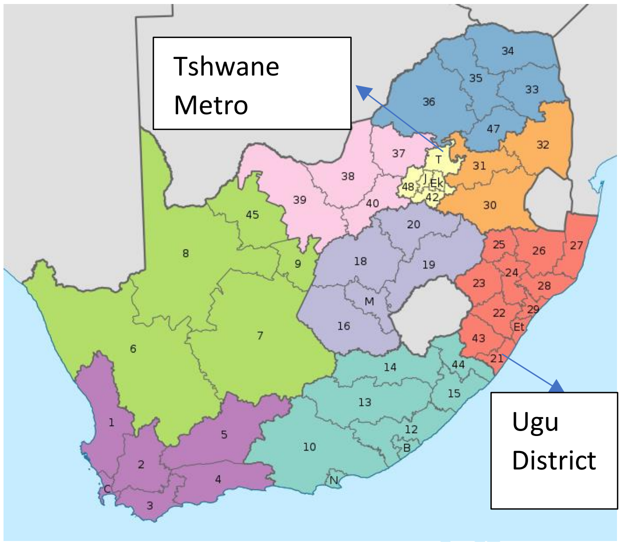

For peer review only - http://bmjopen.bmj.com/site/about/guidelines.xhtml

Figure 2: Theoretical frameworks which informed the development of the intervention

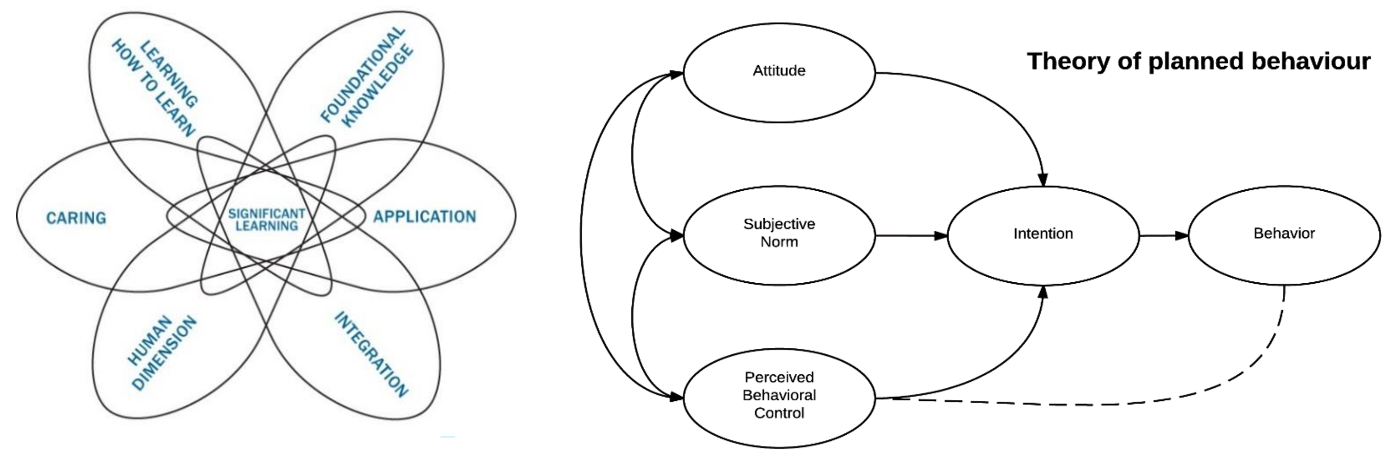

Dee Fink's six-part taxonomy for significant learning

For peer review only

Figure 3: Study population at baseline and follow-up for intervention and comparison sites

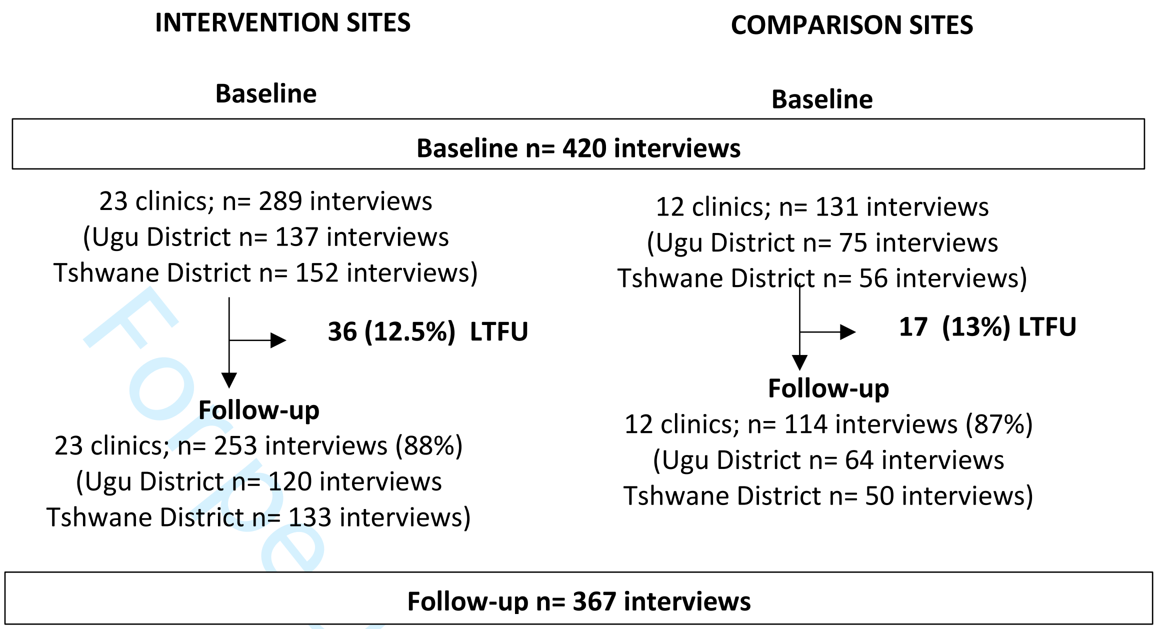

LTFU: lost to follow up

For peer review only

Supplementary Figure 1: Prior training reported by participants at baseline (intervention versus comparison groups)

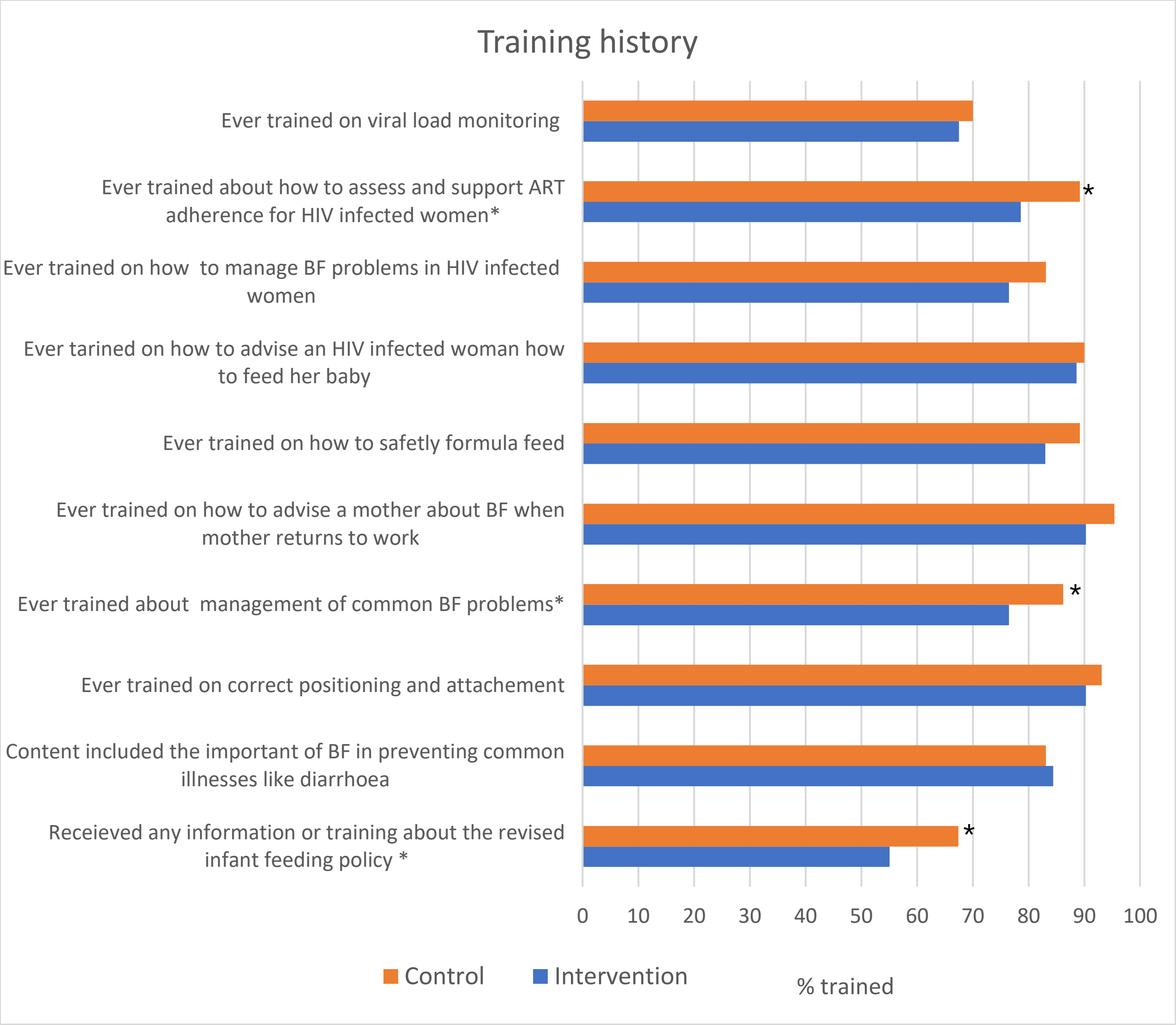

*p<0.05

Abbreviations: ART= antiretroviral therapy; BF= breastfeeding

Supplementary Table 1: Attitude scores of health workers at baseline and follow-up

Attitude statements	Number (%) who agreed or strongly agreed with the statement at baseline			Number (%) who agreed or strongly agreed with the statement at follow-up		
	Intervention (n=289)	Comparison (n=131)	p-value *	Intervention (n=252)	Comparison (n=114)	p-value*
Significant improvements between intervention and comparison groups at follow-up						
There have been so many changes to the infant feeding guidelines and breastfeeding guidelines however I am NOT confused about what to tell mothers who are HIV-infected about breastfeeding	155 (53.6)	74 (56.9)	059	175 (69.4)	65 (57.0)	0.02
For an HIV-exposed infant any breastfeeding is better than no breastfeeding at all, as long as the mother is virally suppressed and on antiretroviral therapy	189 (65.4)	79 (61.2)	0.31	196 (77.8)	74 (64.9)	0.01
When a baby cries all the time it is NOT usually because the baby is hungry and needs more food than just breastmilk	260 (90.0)	107 (83.0)	0.02	230 (91.3)	91(79.8)	<0.01
Exclusive breastfeeding in the first 6 months of life is the best choice for all mothers and babies in South Africa	256 (88.6)	114 (88.4)	0.65	239 (94.8)	98 (86.0)	<0.01
The benefits of breastfeeding for protecting children from illness such as diarrhoea and pneumonia outweighs the risk of acquiring HIV if the mother is on antiretroviral treatment	224 (77.5)	93 (72.1)	0.15	218 (86.5)	87 (76.3)	0.02
I feel that an HIV-infected mother who has not disclosed to her partner is NOT at high risk of non-adherence to ART and should NOT stop breastfeeding as soon as possible	128 (44.3)	51 (39.5)	0.30	137 (54.4)	46 (40.4)	0.01
I should support all mothers, regardless of HIV status, to continue breastfeeding until 2 years, as long as HIV-infected women are virally suppressed	237 (82.0)	101 (78.3)	0.24	237 (94.1)	87 (76.3)	<0.01
I should NOT advise an HIV-positive virally suppressed mother who has cracked and bleeding nipples to temporarily stop breastfeeding	80 (27.7)	40 (31.3)	0.55	97 (38.5)	24 (21.1)	<0.01
Formula feeding is NOT the best choice for mothers living in good socio-economic circumstances who are going back to work	202 (70.0)	95 (74.2)	0.58	198 (78.6)	74 (64.9)	<0.01
It is safer for HIV-positive mothers to breastfeed than to formula feed	231 (79.9)	89 (69.5)	0.63	226 (89.7)	85 (74.6)	<0.01
It is NOT very difficult for mothers to express breastmilk while they are at work or school	164 (53.0)	57 (44.5)	0.01	168 (67.1)	51 (44.7)	<0.01
If an HIV-positive mother can afford to buy formula it is NOT better for her to formula feed her baby	170 (58.8)	83 (64.9)	0.38	185 (73.4)	68 (59.7)	<0.01
Promoting breastfeeding for two years for HIV-exposed infants is NOT a risk because	197 (68.2)	85 (66.4)	0.51	210 (83.3)	79 (69.3)	<0.01

For peer review only - http://bmjopen.bmj.com/site/about/guidelines.xhtml

mothers will be able to maintain good ART adherence for that long						
Low levels of knowledge (<80%) at baseline in both groups - no significant differences between intervention and comparison groups at follow-up [concept that this relates to]						
For an HIV-positive mother on antiretroviral treatment and virally suppressed mixed feeding is better than not breastfeeding at all	39 (13.5)	15 (11.7)	0.56	57 (22.6)	16 (14.0)	0.06
In our community working mothers can successfully maintain exclusive breast feeding while going to work	219 (75.8)	89 (69.5)	0.09	208 (82.5)	92 (80.7)	0.67
There are exceptional circumstances where an HIV-positive mother would be advised not to breastfeed, such as failure of 2nd or 3rd line ART treatment, but these are not common	225 (77.9)	93 (72.7)	0.13	201 (79.7)	87 (76.3)	0.46
High levels of knowledge (≥80%) at baseline in both groups – no significant differences between intervention and comparison groups at follow-up						
HIV-exposed babies who are PCR negative must NOT stop breastfeeding as soon as possible	238 (82.4)	104 (81.3)	0.47	221 (87.7)	95 (83.3)	0.26
Exclusive breastfeeding for six months is an achievable goal for the majority of mothers	249 (86.2)	105 (82.0)	0.11	227 (90.1)	96 (84.1)	0.11
An HIV-positive mother who is on ART and not virally suppressed and is mixed feeding is putting her child at risk of acquiring HIV	256 (88.6)	109 (85.2)	0.13	219 (86.9)	97 (85.1)	0.64
In South Africa it is possible to improve exclusive breastfeeding rates	244 (84.7)	108 (84.4)	0.61	230 (91.3)	97 (85.1)	0.08
Formula feeding is NOT more convenient for a mother than breastfeeding	253 (87.5)	109 (85.2)	0.23	211 (83.7)	100(87.7)	0.32
**Number (%) participants whose attitude was to at least agree (Attitude score ≥84)***	**71 (24.6)**	**23 (17.9)**	**0.12**	**123 (49.2)**	**27 (24.1)**	**<0.01**
**Mean attitude score out of 105 (95% CI)**	**76.9 (75.9 – 77.9)**	**75.0 (73.0 – 77.0)**	**0.07**	**82.7 (81.6 – 83.8)**	**76.8 (75.0 – 78.5)**	**<0.01**

*84 was the minimum score obtainable if a participant at least agreed with all statements

For peer review only

For peer review only - http://bmjopen.bmj.com/site/about/guidelines.xhtml

Supplementary Table 2: Confidence statements of health workers at baseline and follow-up

Confidence statements	Number (%) who felt confident or very confident at baseline			Number (%) who felt confident or very confident at follow-up		
	Intervention (n=289)	Comparison (n=131)	p-value	Intervention (n=252)	Comparison (n=114)	p-value
Significant improvements between intervention and comparison groups at follow-up						
How confident do you feel about counselling an HIV-positive pregnant woman about how she will feed her baby	265 (91.7)	116 (88.6)	0.30	240 (95.2)	100 (87.7)	0.01
How confident do you feel about advising an HIV-positive mother about how to continue to breastfeed her baby when she returns to work or school	258 (89.3)	117 (89.3)	0.99	242 (96.0)	101 (88.6)	<0.01
How confident do you feel about advising an HIV-infected mother to continue breastfeeding for two years	216 (74.7)	105 (80.2)	0.23	236 (93.6)	93 (81.6)	<0.01
How confident do you feel about assessing ART compliance in an HIV-positive mother	240 (83.1)	111 (84.7)	0.67	230 (91.3)	94 (82.5)	0.02
How confident do you feel about advising an HIV-infected mother who is exclusively breastfeeding and has cracked nipples with bloody milk about how to feed her baby	196 (67.8)	84 (64.1)	0.45	201 (79.8)	80 (70.2)	0.04
Low levels of knowledge (<80%) at baseline in both groups - no significant differences between intervention and comparison groups at follow-up [concept that this relates to]						
How confident do you feel about advising an HIV-infected mother about how to stop breastfeeding	214 (74.1)	89 (67.9)	0.20	188 (74.6)	86 (75.4)	0.87
How confident do you feel about identifying when an HIV-positive mother is not adhering to her ART treatment	224 (77.5)	104 (79.4)	0.67	218 (86.5)	90 (79.0)	0.07
How confident do you feel about reassuring a mother living with HIV who is virally suppressed that a shorter duration of breastfeeding is better than never initiating breastfeeding	227 (78.5)	103 (78.6)	0.99	215 (85.3)	96 (84.2)	0.78
How confident do you feel about assisting a mother with HIV to safely formula feed her baby	212 (73.4)	102 (77.9)	0.33	191 (75.8)	95 (83.3)	0.11
How confident do you feel about using the guidelines for safe replacement feeding when you counsel a mother who is not adherent to ART and has a viral load above 1000 copies/ml	191 (66.1)	99 (75.6)	0.05	185 (73.4)	75 (65.8)	0.14
How confident do you feel about advising an HIV-infected mother who is exclusively breastfeeding and has defaulted from her ART about how to feed her baby	205 (70.9)	97 (74.1)	0.51	189 (75.0)	80 (70..2)	0.33
How confident do you feel about managing poor ART compliance in an HIV-infected breastfeeding mother	215 (74.4)	104 (79.4)	0.27	209 (82.9)	89 (78.1)	0.27

For peer review only - http://bmjopen.bmj.com/site/about/guidelines.xhtml

A mother is not adherent to ART and her last viral load is 1000 copies per ml. How confident do you feel about counselling her about feeding her infant?	199 (68.9)	98 (74.8)	0.21	198 (78.6)	80 (70.2)	0.08
High levels of knowledge (≥80%) at baseline in both groups – no significant differences between intervention and comparison groups at follow-up						
How confident do you feel about giving information about the risks and benefits of breastfeeding to an HIV-infected mother	268 (92.7)	120 (91.6)	0.67	238 (94.4)	102 (89.5)	0.09
How confident do you feel about assessing whether there is good positioning and attachment during breastfeeding	263 (91.0)	125 (95.4)	0.11	243(96.4)	107 (93.9)	0.27
How confident do you feel about advising an HIV-infected mother who is virally suppressed who is mixed feeding her infant	243 (84.1)	113 (86.3)	0.57	226 (89.7)	99 (86.8)	0.43
How confident do you feel about advising an HIV-positive mother about starting complementary feeds	251 (86.9)	115 (87.8)	0.79	229 (90.9)	103 (90.4)	0.87
How confident do you feel about explaining the risks of HIV transmission through breastmilk to an HIV-infected mother with high viral load	247 (85.5)	114 (87.0)	0.67	220 (87.3)	105 (92.1)	0.17
How confident do you feel about explaining to a mother about expressing and storing milk	269 (93.1)	124 (94.7)	0.54	237 (94.1)	109 (95.6)	0.54
**Number (%) participants who were confident or very confident (Sum Score ≥ 57)**	**164 (56.8)**	**86 (67.2)**	**0.09**	**175 (70.0)**	**72 (64.3)**	**0.28**
**Mean confidence score out of 76 (95% CI)**	**59.1 (58.0-60.2)**	**59.1 (57.0-61.3)**	**1.0**	**61.2 (60.8-63.1)**	**59.9 (58.1-61.7)**	**0.05**

For peer review only - http://bmjopen.bmj.com/site/about/guidelines.xhtml

Version4_ 14August2017

QUESTIONNAIRE FOR HEALTHCARE WORKERS

For peer review only

For peer review only - http://bmjopen.bmj.com/site/about/guidelines.xhtml

Version4_ 14August2017

Section 1. Admin		
1.1.	Interview Date	☐☐/ ☐☐ ☐ /☐☐☐☐<br>D D M M M Y Y Y Y
1.2.	District	1 OTshwane<br>2 OUgu
1.3.	Sub district	**If Tshwane chosen above:**<br>1 OTshwane 1<br>1 OTshwane 2<br>1 OTshwane 3<br>1 OTshwane 6<br>**If Ugu chosen above:**<br>1 O Omdoni<br>1 O UmZumbe<br>1 O Hibiscus<br>1 O uMuziwabantu
1.4.	Facility	**If Tshwane 1 selected the following clinics can be chosen:**<br>1 O Soshang Block JJ clinic<br>1 O KT Motubatse clinic<br>1 O Boikhutsong clinic<br>1 O Sedilega clinic<br>1 O Shoshanguve 2 clinic<br>1 O Soshang Block TT clinic<br>**If Tshwane 2 selected:**<br>1 O Jubilee gateway clinic<br>1 O Kekanastad clinic<br>1 O Suurman clinic<br>1 O Ramotse clinic<br>1 O Kekana gardens clinic<br>1 O New Eersterus clinic<br>**If Tshwane 3 selected**<br>Atteridgeville Clinic<br>Bophelong Clinic (Tshw 3)<br>Saulsville Clinic

For peer review only

For peer review only - http://bmjopen.bmj.com/site/about/guidelines.xhtml

		**If Tshwane 6 selected**<br>S Bopape CHC<br>Eersterust CHC<br>Nellmapius Clinic<br><br>**If Omdoni selected**<br>GJ Crooke's Gateway<br>Pennington Clinic<br>Philani Clinic<br>Scottburgh Clinic<br>Umzinto Clinic<br><br>**If UmZumbe selected**<br>• Gqayinyanga clinic<br>• St Faiths clinic<br>• Phungashe clinic<br>• Ntimbankulu clinic<br>• Turton CHC<br>• Ndelu clinic<br><br>**If Hibiscus coast selected:**<br>• Gamalakhe CHC<br>• Southport Clinic<br>• Marburg Clinic<br><br>**If uMuziwabantu selected**<br>• Santombe clinic<br>• Meadowsweet clinic<br>• Mbonwa clinic
1.5.	Health worker number	Assigned number to each participant

For peer review only

For peer review only - http://bmjopen.bmj.com/site/about/guidelines.xhtml

Version4_ 14August2017

Section 2. Demographics		
2.1.	What is your date of birth?	□□/□□/□□□□<br>D D M M Y Y Y Y
2.2.	Gender	1 O Male 2 O Female
2.3.	What is your role in this clinic?	1 O Lay counsellor or nutritional advisor<br>2.O Enrolled nurse assistant<br>3.O Enrolled nurse<br>4.O Registered nurse<br>5.O Medical degree (MB ChB or equivalent)<br>6.O community health worker (CCG)<br>7 O: dietician<br>8 O: Registered nurse operational manager<br>9 O: other specify below
2.4.	Other	
2.5.	How long have you been working as a health worker?	1 O less than 1 year<br>2 O 1- <2 years<br>3 O 2- <5 years<br>4 O 5-< 10 years<br>5 O 10 or more years

For peer review only

Version4_ 14August2017

Section 3	**Updated HIV and infant feeding guidelines.** **In this section you will be asked about new infant feeding guidelines adopted in South Africa.**		
3.1	During 2017 have you received any information or training at work about the revised Infant and Young Child Feeding Policy – in the form of a circular, letter, workshop, meeting or lecture	1O Yes	0O No SKIP
3.2	If yes, how did you receive this information?	1. O Circular/letter<br>2. O Meeting<br>3. O Workshop<br>4. O Feedback/information from colleague<br>5. O lecture<br>6. O other	
3.3	Who gave you this information/ training?	1 O District trainer/ DoH staff member<br>2 O Outside/ private company	
3.4	How long was this training?	__________hours	

For peer review only

For peer review only - http://bmjopen.bmj.com/site/about/guidelines.xhtml

Version4_ 14August2017

SECTION 4.	TRAINING Topics<br>Have you received any training on the following topics (either in-service or formal training).<br><br>**Usuke wathola uqeqesho kulezihloko ezilandelayo?**		
4.1	Did the content of your training include the importance of breastfeeding in preventing common childhood illness such as diarrhoea?	1 O Yes	0O No
4.2	Have you ever had any training about correct positioning and attachment of an infant during breastfeeding?	1 O Yes	0O No
4.3	Have you ever had any training about the management of common breastfeeding problems?	1 O Yes	0O No
4.4	Have you ever had any training about advising a mother about how to provide breastmilk for her baby when she returns to work or school	1 O Yes	0O No
4.5	Have you ever had any training about how to advise a mother about formula feeding safely?	1 O Yes	0O No
4.6	Have you ever had any training about how to advise an HIV infected woman about how to feed her baby?	1 O Yes	0O No
4.7	Have you ever had any training about how to manage breastfeeding problems in HIV infected women (cracked nipples, mastitis etc.)?	1 O Yes	5.0 O No
4.8	Have you ever had any training about how to assess and support ART adherence for HIV infected women?	1 O Yes	0 O No
4.9	Have you ever had any training on viral load monitoring?	1 O Yes	0 O No

For peer review only - http://bmjopen.bmj.com/site/about/guidelines.xhtml

Version4_ 14August2017

Section 5	**ACTIVITIES:**<br>***Think carefully about your work in this facility.*** For the activity mentioned consider whether you ever perform this activity and if so how regularly do you perform this activity? If you do not perform this activity at all select the option 'Never'				
5.1	How often do you talk to groups of pregnant women attending the antenatal clinic about infant feeding (group counselling)	1 O one or more times per week	2 O one to three times per month	3 O Less than once a month	4 O Never
5.2	How often do you talk to a pregnant woman individually about her plan for feeding her baby	1 O one or more times per week	2 O one to three times per month	3 O Less than once a month	4 O Never
5.3	How often do you talk to an HIV infected pregnant woman about her plan for feeding her baby	1 O one or more times per week	2 O one to three times per month	3 O Less than once a month	4 O Never
5.4	How often do you assist a mother with breastfeeding within the first hour post delivery	1 O one or more times per week	2 O one to three times per month	3 O Less than once a month	4 O Never
5.5	How often do you talk to a mother about how she is feeding her baby?	1 O one or more times per week	2 O one to three times per month	3 O Less than once a month	4 O Never
5.6	How often do you observe a mother breastfeeding during a clinic or home visit	1 O one or more times per week	2 O one to three times per month	3 O Less than once a month	4 O Never
5.7	How often do you talk to a mother about positioning and attachment of the baby during breastfeeding	1 O one or more times per week	2 O one to three times per month	3 O Less than once a month	4 O Never
5.8	How often do you talk to an HIV infected mother about managing a breastfeeding problem (e.g. cracked nipples, baby crying all the time, mother says she does not have enough milk)	1 O one or more times per week	2 O one to three times per month	3 O Less than once a month	4 O Never
5.9	How often do you talk to a mother about how to maintain breastfeeding when away from the baby (going back to school or work)	1 O one or more times per week	2 O one to three times per month	3 O Less than once a month	4 O Never
5.10	How often do you talk to an HIV infected breastfeeding mother about taking ARVs	1 O one or more times per week	2 O one to three times per month	3 O Less than once a month	4 O Never

For peer review only - http://bmjopen.bmj.com/site/about/guidelines.xhtml

Version4_ 14August2017

SECTION 6	INFANT FEEDING KNOWLEDGE			
please state whether the statement is true or false or you do not know				
	**Statement**	**True**	**False**	**Do not know**
6.1	Exclusive breastfeeding is the recommended infant feeding method for ALL infants aged 0-6 months in SA, regardless of mother's HIV status	1 O	2 O	3 O
6.2	Giving any formula milk during the first six months of life increases the risk of death from diarrhoea and/or pneumonia	1 O	2 O	3 O
6.3	Continued breastfeeding for 2 years is the recommended infant method in SA for ALL children, regardless of mother's HIV status	1 O	2 O	3 O
6.4	Mothers living with HIV who are receiving antiretroviral treatment and are virally suppressed should be advised not to breastfeed their infants	1 O	2 O	3 O
6.5	When an HIV infected mother is ready to add complementary feeds she should stop breastfeeding rapidly over a 24hour period	1 O	2 O	3 O
6.6	If an HIV exposed baby is receiving both breastmilk and formula milk, the mother should chose to either breastfeeding or formula feeding if she is adherent to ART	1 O	2 O	3 O
6.7	A mother who is working and giving formula milk should mix the milk herself and leave for the carer to give during the day	1 O	2 O	3 O
6.8	An HIV positive mother who is virally suppressed on antiretroviral treatment should breastfeed her child rather than not breastfeed to improve the child's survival	1 O	2 O	3 O
6.9	When sterilising feeding bottles cover the bottles with water in a saucepan and place on the heat. As soon as the water boils remove from heat and leave the bottle in the water until completely cool	1 O	2 O	3 O
6.10	In South Africa, the leading cause of death amongst children under 5 is pneumonia	1 O	2 O	3 O
6.11	In South Africa, HIV infected women who are breastfeeding should be supported to adhere to antiretroviral treatment and should be counselled and supported to exclusively breastfeed their infants for the first six months of life whilst maintaining an undetectable viral load	1 O	2 O	3 O
6.12	A baby under 4 months should be given soft porridge once he/she seems hungry	1 O	2 O	3 O

For peer review only - http://bmjopen.bmj.com/site/about/guidelines.xhtml

Version4_ 14August2017

6.13	A mother living with HIV and adherent to antiretroviral treatment cannot exclusively breastfeed her 4-month old infant because she is working. It is better for this mother to give formula during the day and breastfeed at night rather than giving no breast milk at all	1 ○	2 ○	3 ○
6.14	An HIV exposed baby who is exclusively breastfeeding should be given some water when the weather is very hot	1 ○	2 ○	3 ○
6.15	If a baby has a positive PCR (HIV test) at birth the mother should stop breastfeeding if this is affordable and feasible in her situation	1 ○	2 ○	3 ○
6.16	Giving a baby expressed breastmilk is not as good as breastfeeding	1 ○	2 ○	3 ○
6.17	If a mother misses 2 doses of her ART in one month, she should be classified as a treatment failure	1 ○	2 ○	3 ○
6.18	An HIV positive mother who has cracked nipples should continue to breastfeed unless they are bleeding	1 ○	2 ○	3 ○
6.19	A mother who has missed 6 tablets of FDC in one month is considered to be poorly adherent and should stop breastfeeding immediately	1 ○	2 ○	3 ○
6.20	There are long term health benefits of breastfeeding for mother and child that last beyond the breastfeeding period	1 ○	2 ○	3 ○
6.21	In South Africa, HIV infected women who are breastfeeding should be supported to adhere to antiretroviral treatment and should introduce complementary foods around 6 months and be supported to continue breastfeeding for at least two years.	1 ○	2 ○	3 ○
6.22	It is safe to give the baby expressed breastmilk that has been kept outside the fridge for 8 hours	1 ○	2 ○	3 ○

For peer review only

For peer review only - http://bmjopen.bmj.com/site/about/guidelines.xhtml

Version4_ 14August2017

SECTION 7.		INFANT FEEDING ATTITUDE<br><br>Please state whether you completely disagree, disagree, neutral, agree or completely agree with the statement				
		Completely disagree	Disagree	Neutral	Agree	Completely agree
7.1	There have been so many changes to the infant feeding guidelines and breastfeeding guidelines that I am confused about what to tell mothers who are HIV infected about	1 ○	2 ○	3 ○	4 ○	5 ○
7.2	When a baby cries all the time it is usually because the baby is hungry and needs more food than just breastmilk	1 ○	2 ○	3 ○	4 ○	5 ○
7.3	Exclusive breastfeeding in the first 6 months of life is the best choice for all mothers and babies in South Africa	1 ○	2 ○	3 ○	4 ○	5 ○
7.4	For an HIV exposed infant any breastfeeding is better than no breastfeeding at all, as long as the mother is virally suppressed and on antiretroviral therapy	1 ○	2 ○	3 ○	4 ○	5 ○
7.5	The benefits of breastfeeding for protecting children from illness such as diarrhoea and pneumonia outweighs the risk of acquiring HIV if the mother is on antiretroviral treatment	1 ○	2 ○	3 ○	4 ○	5 ○
7.6	I feel that an HIV infected mother who has not disclosed to her partner is at high risk of non-adherence to ART and should stop breastfeeding as soon as [illegible]	1 ○	2 ○	3 ○	4 ○	5 ○
7.7	I should support all mothers, regardless of HIV status, to continue breastfeeding until 2 years, as long as HIV infected women are virally suppressed	1 ○	2 ○	3 ○	4 ○	5 ○
7.8	I should advise an HIV positive virally suppressed mother who has cracked and bleeding nipples to temporarily stop breastfeeding	1 ○	2 ○	3 ○	4 ○	5 ○
7.9	HIV exposed babies who are PCR negative must stop breastfeeding as soon as possible	1 ○	2 ○	3 ○	4 ○	5 ○

For peer review only

Version4_ 14August2017

7.10	Formula feeding is the best choice for mothers living in good socioeconomic circumstances who are going back to work	1 O	2 O	3 O	4 O	5 O
7.11	For an HIV positive mother on antiretroviral treatment and virally suppressed mixed feeding is better than not breastfeeding at all	1 O	2 O	3 O	4 O	5 O
7.12	Exclusive breastfeeding for six months is an achievable goal for the majority of mothers	1 O	2 O	3 O	4 O	5 O
7.13	It is safer for HIV positive mothers to breastfeed than to formula feed	1 O	2 O	3 O	4 O	5 O
7.14	In our community working mothers can successfully maintain exclusive breast feeding while going to work	1 O	2 O	3 O	4 O	5 O
7.15	An HIV positive mother who is on ART and not virally suppressed and is mixed feeding is putting her child at risk of acquiring HIV	1 O	2 O	3 O	4 O	5 O
7.16	It is very difficult for mothers to express breastmilk while they are at work or school	1 O	2 O	3 O	4 O	5 O
7.17	If an HIV positive mother can afford to buy formula it is better for her to formula feed her baby	1 O	2 O	3 O	4 O	5 O
7.18	Promoting breastfeeding for two years for HIV exposed infants is a risk because mothers will be unable to maintain good ART adherence for that long	1 O	2 O	3 O	4 O	5 O
7.19	In South Africa it is possible to improve exclusive breastfeeding rates	1 O	2 O	3 O	4 O	5 O
7.20	There are exceptional circumstances where an HIV positive mother would be advised not to breastfeed, such as failure of 2nd or 3rd line ART treatment, but these are not common	1 O	2 O	3 O	4 O	5 O
7.21	Formula feeding is more convenient for a mother than breastfeeding	1 O	2 O	3 O	4 O	5 O

For peer review only - http://bmjopen.bmj.com/site/about/guidelines.xhtml

Version4_ 14August2017

**SECTION 8.**	**INFANT FEEDING COUNSELLING CONFIDENCE**<br><br>For each activity below, please indicate how confident you feel to undertake each activity. Do you feel "Not at all confident", "Not very confident", "Somewhat confident" or "Very confident".				
		Not at all confident	Not very confident	Confident	Very confident
8.1	How confident do you feel about counselling an HIV positive pregnant woman about how she will feed her baby	1 ○	2 ○	3 ○	4 ○
8.2	How confident do you feel about giving information about the risks and benefits of breastfeeding to an HIV infected mother	1 ○	2 ○	3 ○	4 ○
8.3	How confident do you feel about assessing whether there is good positioning and attachment during breastfeeding	1 ○	2 ○	3 ○	4 ○
8.4	How confident do you feel about advising an HIV positive mother about how to continue to breastfeed her baby when she [illegible]	1 ○	2 ○	3 ○	4 ○
8.5	How confident do you feel about advising an HIV infected mother who is virally suppressed who is mixed feeding her infant	1 ○	2 ○	3 ○	4 ○
8.6	How confident do you feel about advising an HIV infected mother to continue breastfeeding for two years	1 ○	2 ○	3 ○	4 ○
8.7	How confident do you feel about advising an HIV infected mother about how to stop breastfeeding	1 ○	2 ○	3 ○	4 ○
8.8	How confident do you feel about advising an HIV positive mother about starting complementary feeds	1 ○	2 ○	3 ○	4 ○
8.9	How confident do you feel about assessing ART compliance in an HIV positive mother	1 ○	2 ○	3 ○	4 ○
8.10	How confident do you feel about identifying when an HIV positive mother is not adhering to her ART treatment	1 ○	2 ○	3 ○	4 ○
8.11	How confident do you feel about reassuring a mother living with HIV who is virally suppressed that a shorter duration of breastfeeding is better than never initiating breastfeeding	1 ○	2 ○	3 ○	4 ○
8.12	How confident do you feel about explaining the risks of HIV transmission through breastmilk to an HIV infected mother with high viral load	1 ○	2 ○	3 ○	4 ○

For peer review only

Version4_ 14August2017

8.13	How confident do you feel about assisting a mother with HIV to safely formula feed her baby	1 O	2 O	3 O	4 O
8.14	How confident do you feel about advising an HIV infected mother who is exclusively breastfeeding and has cracked nipples with bloody milk about how to feed her baby	1 O	2 O	3 O	4 O
8.15	How confident do you feel about using the guidelines for safe replacement feeding when you counsel a mother who is not adherent to ART and has a viral load above 1000 copies/ml	1 O	2 O	3 O	4 O
8.16	How confident do you feel about advising an HIV infected mother who is exclusively breastfeeding and has defaulted from her ART about how to feed her baby	1 O	2 O	3 O	4 O
8.17	How confident do you feel about explaining to a mother about expressing and storing milk	1 O	2 O	3 O	4 O
8.18	How confident do you feel about managing poor ART compliance in an HIV infected breastfeeding mother	1 O	2 O	3 O	4 O
8.19	A mother is not adherent to ART and her last viral load is 1000 copies per ml. How confident do you feel about counselling her about feeding her infant?	1 O	2 O	3 O	4 O

For peer review only

For peer review only - http://bmjopen.bmj.com/site/about/guidelines.xhtml

Translating new evidence into clinical practice: A quasi-experimental controlled before-after study evaluating the effect of a novel outreach mentoring approach on knowledge, attitudes and confidence of health workers providing HIV and infant feeding counselling in South Africa

Journal:	*BMJ Open*
Manuscript ID	bmjopen-2019-034770.R3
Article Type:	Original research
Date Submitted by the Author:	16-Aug-2020
Complete List of Authors:	Goga, Ameena; Medical Research Council of South Africa, Health Systems Research Unit and HIV Prevention Research Unit; University of Pretoria Faculty of Health Sciences, Paediatrics<br>Doherty, Tanya; South African Medical Research Council<br>Manda, Samuel; South African Medical Research Council<br>Nkwenika, Tshifhiwa; SAMRC<br>Haskins, Lyn; University of KwaZulu-Natal<br>John, Vaughn; University of KwaZulu-Natal, School of Education<br>Engebretsen, Ingunn; University of Bergen Faculty of Medicine and Dentistry, Centre for International Health<br>Feucht, Ute; University of Pretoria<br>Dhansay, Ali; Burden of Disease Research Unit<br>Rollins, Nigel; World Health Organization, Department of Maternal, Newborn, Child and Adolescent Health<br>Kroon, Max; University of Cape Town Faculty of Health Sciences, Neonatology<br>Sanders, David; University of the Western Cape, South Africa , School of Public Health<br>Kauchali, Shuaib; South African National Department of Health<br>Tylleskär, Thorkild; Universitetet i Bergen, Centre for International health<br>Horwood, Christiane ; University of KwaZulu-Natal,
**Primary Subject Heading**:	Global health
Secondary Subject Heading:	Public health
Keywords:	Public health < INFECTIOUS DISEASES, HIV & AIDS < INFECTIOUS DISEASES, Community child health < PAEDIATRICS

SCHOLARONE™
Manuscripts

I, the Submitting Author has the right to grant and does grant on behalf of all authors of the Work (as defined in the below author licence), an exclusive licence and/or a non-exclusive licence for contributions from authors who are: i) UK Crown employees; ii) where BMJ has agreed a CC-BY licence shall apply, and/or iii) in accordance with the terms applicable for US Federal Government officers or employees acting as part of their official duties; on a worldwide, perpetual, irrevocable, royalty-free basis to BMJ Publishing Group Ltd ("BMJ") its licensees and where the relevant Journal is co-owned by BMJ to the co-owners of the Journal, to publish the Work in this journal and any other BMJ products and to exploit all rights, as set out in our licence.

The Submitting Author accepts and understands that any supply made under these terms is made by BMJ to the Submitting Author unless you are acting as an employee on behalf of your employer or a postgraduate student of an affiliated institution which is paying any applicable article publishing charge ("APC") for Open Access articles. Where the Submitting Author wishes to make the Work available on an Open Access basis (and intends to pay the relevant APC), the terms of reuse of such Open Access shall be governed by a Creative Commons licence – details of these licences and which Creative Commons licence will apply to this Work are set out in our licence referred to above.

Other than as permitted in any relevant BMJ Author's Self Archiving Policies, I confirm this Work has not been accepted for publication elsewhere, is not being considered for publication elsewhere and does not duplicate material already published. I confirm all authors consent to publication of this Work and authorise the granting of this licence.

For peer review only

Translating new evidence into clinical practice: A quasi-experimental controlled before-after study evaluating the effect of a novel outreach mentoring approach on knowledge, attitudes and confidence of health workers providing HIV and infant feeding counselling in South Africa

Ameena Goga[1, 2, 3], Tanya Doherty[1,4,5], Samuel Manda[6, 7], Tshifhiwa Nkwenika[6], Lyn Haskins[8], Vaughn John[9], Ingunn M.S. Engebretsen[10], Ute Feucht[2, 11,12, 13], Ali Dhansay[14, 15] Nigel Rollins[16], Max Kroon[17, 18], David Sanders[4,19], Shuaib Kauchali[20], Thorkild Tylleskar[10,] Christiane Horwood[8]

Affiliations

[1]Health Systems Research Unit, South African Medical Research Council, South Africa
[2]Department of Paediatrics, University of Pretoria, South Africa
[3]HIV Prevention Research Unit, South African Medical Research Council, South Africa
[4]School of Public Health, University of the Western Cape, South Africa
[5]School of Public Health, University of the Witwatersrand
[6]Biostatistics Unit, South African Medical Research Council, South Africa
[7]Department of Statistics, University of Pretoria, South Africa
[8]Centre for Rural Health, University of KwaZulu-Natal, South Africa
[9]School of Education, University of KwaZulu Natal, Pietermaritzburg, South Africa
[10]Centre for International Health, University of Bergen, Norway
[11]Tshwane District Health Services, Gauteng Department of Health, city? South Africa
[12]Research Centre for Maternal, Fetal, Newborn and Child Health Care Strategies, University of Pretoria, Pretoria, South Africa
[13]Maternal and Infant Health Care Strategies Research Unit, South African Medical Research Council, South Africa
[14]Division of Human Nutrition and Department of Paediatrics and Child Health, Stellenbosch University, South Africa.
[15]Burden of Disease Research Unit, South African Medical Research Council, South Africa
[16]Department of Maternal, Newborn, Child and Adolescent Health, World Health Organization, Geneva, Switzerland
[17]Department of Neonatology, Faculty of Health Sciences, University of Cape Town
[18]Mowbray Maternity Hospital, Cape Town, South Africa
[19]Department of Paediatrics and Child Health, University of Cape Town, South Africa
[20]National Department of Health, South Africa

Corresponding author: Ameena.Goga@mrc.ac.za

For peer review only

Abstract

Objectives: We report the effectiveness of a mentoring approach to improve health workers' knowledge, attitudes and confidence with counselling on HIV and infant feeding.
Design: Quasi-experimental controlled before-after study.
Setting: Randomly selected primary health care clinics (n=24 intervention, n=12 comparison); two districts, South Africa.
Participants: All health workers (HW) providing infant feeding counselling in selected facilities were invited.
Interventions: three 1-2 hour, on-site workshops over 3-6 weeks.
Primary outcome measures: Knowledge (22 binary questions), attitude (21 questions - 5-point Likert scale) and confidence (19 questions - 3-point Likert scale). Individual item responses were added within each of the attitude and confidence domains. The respective sums were taken to be the domain composite index and used as a dependent variable to evaluate intervention effect. Linear regression models were used to estimate the mean score difference between intervention and comparison groups post-intervention, adjusting for the mean score difference between them at baseline. Analyses were adjusted for participant baseline characteristics and clustering at health facility level.
Results: In intervention and comparison sites, respectively: 289 and 131 baseline and 253 and 114 follow-up interviews were conducted (August-December 2017). At baseline there was no difference in mean number of correctly answered knowledge questions; this differed significantly at follow-up (15.2 in comparison; 17.2 in intervention sites ($p<0.001$)). At follow-up, the mean attitude and confidence scores towards breastfeeding were better in intervention versus comparison, sites ($p<0.001$ and $p=0.05$, respectively). Controlling for possible confounders, interactions between time and intervention group and pre-intervention values, the attitude score was 5.1-points significantly higher in intervention versus comparison groups.
Conclusion: A participatory, low intensity on-site mentoring approach to disseminating updated infant feeding guidelines improved HWs' knowledge, attitudes and confidence more than standard dissemination via a circular. Further research is required to evaluate the effectiveness, feasibility and sustainability of this approach at scale.

Strengths and Limitations of this study:

1. Fieldwork was conducted in two geographically and historically different provinces, facilitating generalisability of results.
2. The intervention was participatory, low intensity, on-site and integrated into routine services.
3. Several types of analyses were conducted which all yielded congruent results.
4. However, limitations were that (i) we purposively selected districts for inclusion (ii) we could not control for HWs' personal breastfeeding experience as we did not gather these data (iii) the follow-up evaluation was undertaken 3 months after the intervention - thus, we measured short term benefits, and (iv) we did not measure the direct effect of improved HWs' knowledge, attitudes and confidence on health workers' counselling and mothers' infant feeding practices and (v) we did not co-design the intervention with women living with HIV. Co-designing the intervention with others living with HIV may have resulted in a different intervention and results, and needs to be undertaken in future work.

For peer review only - http://bmjopen.bmj.com/site/about/guidelines.xhtml

5. The finding that knowledge scores amongst participants who attended 3 workshops were significantly better than knowledge scores amongst participants who attended less than 3 workshops, may simply reflect better motivation amongst attendees of more workshops, rather than the effect of the workshops themselves. We could not tease out these effects.

Funding statement

This study was funded by the World Health Organization (Ref: 2017/712509-0). Tanya Doherty receives a from the National Research Foundation, South Africa. Ameena Goga and Tanya Doherty's time was funded by the South African Medical Research Council. The publication of paper was funded by the South African Medical Research Council.

Competing interests statement

No author has declared any competing interests.

Introduction

The benefits of breastfeeding in all settings, and particularly in low-middle income settings with high HIV prevalence, are undisputed.[1 2] Policies and clinical practice guidelines on preventing vertical transmission of HIV (PVT), also known as preventing mother to child transmission of HIV (PMTCT) and infant feeding have undergone frequent evidence-based revisions. For example South African PMTCT policy and its accompanying infant feeding recommendations have been revised five times since 2001 (2008, 2010, 2013, 2015, 2019).[3-7] Additionally, in 2011 a national infant feeding declaration withdrew free commercial infant formula as part of the PVT programme [8], and in 2017 the infant and young child feeding policy was updated to recommend that women living with HIV may continue breastfeeding for up to 24 months or longer (similar to the general population) while being fully supported for antiretroviral therapy (ART) adherence. This followed a 2016 World Health Organization (WHO) update which also stated that mixed feeding is not a reason to stop breastfeeding in the presence of ARV drugs.[9] However, a key gap is that these policies have not been effectively communicated to all health workers – a requirement of the Mother-Baby Friendly Initiative.[10 11] Health workers play a critical role in guiding infant feeding choices and sustaining infant feeding practices [11-13]; they wield power and authority [12 14] but their potentially positive influence on infant feeding is compromised by confusion over HIV and infant feeding, which has eroded their confidence.[11 13] Identifying and implementing optimal strategies to effectively disseminate updated guidelines have lagged behind. Multi-component dissemination strategies, which aim to increase the reach, ability and motivation of health workers, are more effective than one strategy alone.[15] However, in reality there are few published studies that inform guideline dissemination. Most of these are from high-income settings and may not be relevant to low-income settings which have unique challenges.[15]

Research has demonstrated that improving HWs' capacity can significantly improve their skills, self-efficacy and confidence to counsel, support and promote breastfeeding among women living

For peer review only - http://bmjopen.bmj.com/site/about/guidelines.xhtml

with HIV.[16 17] Consequently, a key question was: What learning approach could best develop health worker capacity and confidence to implement the updated HIV and infant feeding guideline, using a methodology that is sustainable and feasible to implement at scale? Pedagogical research highlights the advantage of participatory training compared with standard didactic teaching for improving health worker skills.[18 19] Thus, we sought to determine whether a participatory outreach mentorship approach to disseminate the updated HIV and infant feeding guidelines, using simple low-technology activities, improves health workers' knowledge of, attitudes towards and confidence with counselling on HIV and infant feeding. We chose to focus on health workers knowledge, attitudes and confidence as health workers in South Africa consider themselves as advocates for babies.[20] Additionally, they are one of the key influential groups in the complex socio-ecology of infant feeding.[12 13 21 22]

Methods

Study design

A quasi-experimental before-after design with intervention and comparison sites was used. Two purposively selected districts (Ugu and Tshwane District) in South Africa in each of two geographically disparate provinces, KwaZulu-Natal (KZN) and Gauteng (Figure 1), were included. Both provinces experienced a policy change in June 2017, when the 2013 South African Infant and Young Child Feeding Policy was amended to align with the 2016 WHO/UNICEF update on HIV and Infant feeding guideline.

Sampling

In Ugu District all four sub-districts were selected; within Tshwane District two of the seven service delivery regions were randomly selected.

Twelve intervention and six comparison primary health care clinics were randomly sampled in Ugu District and Tshwane District (separately). Only clinics with above the median number of annual clinic visits for children under-5 years in the district were eligible for inclusion in the sampling frame. The comparison clinics served to capture any temporal changes in health worker knowledge, confidence and attitudes due to other interventions or trainings; hence a smaller sample was required in comparison versus intervention sites as the latter required more precise estimates of the intervention effect. A two-stage process was used to recruit participants. Firstly, research staff explained the study and participant inclusion and exclusion criteria to each facility manager during face-to-face on-site introductory meetings. The facility manager compiled a list of all eligible health workers involved in the care of pregnant women and children, including nurses, midwives, visiting doctors, lay counsellors, dieticians, nutritionists, facility managers and community health workers (CHWs). In the second stage, research staff approached eligible health workers and invited them to participate in the research. We aimed to recruit a manageable size of 8-10 health workers per clinic for participation in the intervention, and in the evaluation. The same staff were approached for the baseline and follow-up evaluations.

Sample size

The sample size was determined based on 80% power and alpha 0.05 to measure a 15-percentage points difference in health worker confidence in HIV and infant feeding counselling between the intervention and comparison clinics comparing baseline and follow-up. The expected effect was

For peer review only - http://bmjopen.bmj.com/site/about/guidelines.xhtml

based on the researchers' experience and data from recent studies in South Africa with the baseline level of high confidence to counsel HIV-positive women on breastfeeding duration set at 45%.[23] It was assumed that the confidence score would remain unchanged in the comparison clinics, implying a two-sample test in the post-intervention period. Clinic-level analyses were used for the sample size calculations, assuming a sampling ratio of 2:1 for the intervention clinics and a standard deviation of 15% in the mean score between clinics. Based on these assumptions, and adjusting for clustering, the sample size was determined to be 24 intervention clinics and 12 comparison clinics.[24] Within the intervention and comparison clinics, all health workers (nurses, midwives, visiting doctors, lay counsellors, dieticians, nutritionists, facility managers and CHWs), involved in caring for pregnant women and children were invited to participate in the study - we anticipated a mean number of health workers per participating facility to be 8-10.

Description of the intervention

We designed a participatory intervention comprising on-site mentoring through three workshops in each clinic, involving 303 selected health workers who provide care for pregnant women, breastfeeding mothers and their infants. This mentoring approach had five distinct features: 1) on-site: learning occurred in context 2) open to all cadres of health workers; 3) team-based; participants learned together; 4) content was led by self-identified gaps in knowledge and 5) activities were piloted and rooted in a theoretical framework. The intervention was delivered by the same trained facilitator (a nurse in Gauteng and nutritionist in KZN) in each intervention clinic. Each workshop lasted 1-2 hours over a 3-6-week period and had well-defined learning outcomes. The intervention has been described elsewhere.[25] In summary, our participatory intervention was guided by evidence that health workers' attitudes and practices are influenced by various factors, not just exposure to training and information.[26] We used Dee Fink's six part taxonomy as a guiding theory. This proposes that significant learning only occurs by developing foundational knowledge, applying skills, integrating ideas, developing new feelings/interests and values, and learning how to learn (encouraging the spirit of enquiry) (Figure 2).[27] Additionally, we applied the Theory of Planned Behaviour to the intervention design (Figure 2).[28 29] This states that an individual's intention to perform a behaviour is influenced by the person's attitudes towards performing the behaviour, their beliefs about whether people who are important to them will approve of the behaviour (subjective norms), and their beliefs about how likely they are to be able to implement the behaviour successfully. According to this theory, if health workers are to provide infant feeding counselling and support in accordance with updated infant feeding guidelines to HIV-positive or negative mothers, they need to agree with the change, believe that their colleagues and other stakeholders will approve of the action, and believe in their ability to implement it successfully. The workshops were tailored to achieve these goals: Workshop 1 covered knowledge gaps reported by participants, controversial statements, and advantages of breastfeeding. Following workshop 1, a poster or cards with key messages were placed in a prominent place in the clinic. Workshop 2 comprised a progressive case study discussed in small groups. Workshop 3 involved one-to-one mentorship: each participant was observed providing infant feeding counselling or a case study was discussed if no mothers were available for counselling. The same facilitator conducted all three workshops at each clinic. In addition, a WhatsApp cell phone messaging group was established to support participants in intervention sites to facilitate sharing of concerns, tips for counselling and dealing with difficult situations. Key messages were posted

For peer review only - http://bmjopen.bmj.com/site/about/guidelines.xhtml

on the group approximately weekly. Comparison and intervention sub-districts were exposed to routine supervision and training activities that took place at district level. The study team documented that the June 2017 circular issued by the National Department of Health, informing health facilities of the change in Infant and Young Child feeding policy, was disseminated to comparison clinics as an announcement via e-mail and other electronic communication as well as during meetings or trainings. We documented that in Tshwane, 15 of the 18 clinics had received the circular; 11 via e-mail and three at a meeting. In Ugu nine of 17 clinics had received the circular; 8 received it via hand delivery and one via e-mail.

Patient and Public Involvement

Patients and the public were not involved in the design of this study, as the main population of interest were health workers. The intervention and tool were piloted amongst a separate group of health workers to determine length, complexity of questions and level of understanding. These details are explained in our intervention paper.[25]

Data collection

Data were collected between August and December 2017 by dedicated trained non-nurse data collectors who were independent of the intervention staff. As per study design, data collection staff were not part of any intervention activities and had never been exposed to the intervention. The primary outcome measure for the study was the confidence level of health workers to counsel on infant feeding, evaluated using a Likert-scale tool, developed after reviewing the WHO Breastfeeding Counselling Course, and the WHO HIV and Infant feeding counselling courses. [9 30-33], see tool in Supplementary material. Secondary outcomes included health worker knowledge and attitude about breastfeeding counselling. A baseline assessment amongst all participating health workers in intervention and comparison sites was undertaken prior to the start of the intervention (August 2017). Health workers self-completed the assessment on study-provided electronic tablets at their workplaces. Questions covered basic demographic information, types of activities undertaken at work, knowledge, attitudes and confidence around counselling on infant feeding, see tool in Supplementary material. Approximately 12 weeks after the baseline assessment, a follow-up assessment using the same tool was conducted amongst the same group of health workers. Health workers who were not in the clinic on the day of the follow-up assessments were included in a special catch-up assessment. Questionnaire software had built in range and skip logic and data were transferred automatically to a database held at the University of KwaZulu-Natal.

Data analysis

There were three outcomes in the study: a) 22 knowledge statements which were scored 1 if correctly answered and 0 if not; evaluation of answers were based on existing literature and guidelines (binary outcomes); b) 21 attitude questions whose responses were measured on a 5-point Likert scale - given as completely disagree (1); disagree (2); neutral (3); agree (4) and completely agree (5); positive attitudes received higher scores; and c) 19 statements on confidence item questions which were also measured on a Likert scale, scored as such: not at all confident (1), not confident (2), confident (3) and very confident (4). For both attitude and confidence domains, a participant outcome was measured by the sum of the responses to the respective items (we verified that there was not a missing response on the items). Thus, the ranges for the attitude and confidence scores were 21 to 105, and 19 to 75, respectively.

For peer review only - http://bmjopen.bmj.com/site/about/guidelines.xhtml

Participants baseline and follow-up characteristics and outcomes between the intervention and control areas were compared using Chi-squared tests for categorical variables and two-sample t tests for continuous measures, after confirming that data were normally distributed. To assess the effect of the proposed intervention, several analysis methods for comparing intervention effect in before (pre)-after (post) quasi-experimental designs were considered. These included using post-measures and change from pre-intervention to post-intervention as the response variables. These approaches that use change and post measurements as the outcome, adjusting for pre-intervention measurements are recommended, and often give similar results.[34] In this paper, we considered three methods for estimating and testing the intervention effect using the sum of individual attitude or confidence scores as an outcome variable in a linear regression. The first method used the post-intervention measurements as the outcome variable but adjusted for the pre-intervention values; The second method analysed the change score as an outcome variable adjusting for pre-treatment values. The third method analysed the vectors of pre-and post-measurements as the outcome variable, and used time (coded 1 at follow-up and 0 at baseline) and treatment (coded:1 intervention group and 0: comparison group) as a covariates with an interaction term for time and treatment, in addition to an adjustment for pre-treatment values). Using methods 1 and 2 the coefficient for the intervention estimated the differences in the post intervention means and differences in the mean change of sum scores mean between the groups, controlling for the pre-intervention measurement. Using the third method, the sum of coefficients of intervention and the interaction terms was taken as the mean difference between groups post-treatment, allowing for pre-treatment mean differences between the groups. All analyses also controlled for baseline participant characteristics and prior training. Analyses adjusted for possible clustering effect at the site level, using a variance-correction method.[24] All the treatment effect comparison analyses were done on an intention to treat, rather than per-protocol, basis, Data can be obtained by e-mailing the corresponding author.

Ethics

Ethics approval was obtained from the South African Medical Research Council (EC028-9/2016), the University of KwaZulu-Natal (RECIP348/17) and the WHO Ethics Review Committee (ERC0002833)**.** Permission for undertaking the study was obtained from Tshwane and KZN Districts. Informed consent was sought from all study participants and no personal identifying information was captured in the questionnaires, only a study identification number.

Results

At baseline and follow-up, 23 intervention clinics (one large clinic was sampled twice with two rounds of data collection per time point) and 12 comparison clinics were visited; 289 and 131 health worker interviews were conducted at baseline in intervention and comparison clinics, respectively (Figure 3). Loss to follow-up between baseline and follow-up did not differ between intervention and comparison sites (17 (13.0%) in comparison sites versus 36 (12.5%) in intervention sites).

Tshwane and Ugu Districts did not differ in the main outcome measures at baseline (knowledge, attitude and confidence). Additionally, they were similar in all health worker characteristics except three: Tshwane had significantly more participants with less than 2 years employment (14.4%

For peer review only - http://bmjopen.bmj.com/site/about/guidelines.xhtml

versus 6.2%, p=0.007), more registered nurses (57% versus 26.2%, p=0.02), and fewer lay counsellors/CHWs (7.3% versus 50.0%, respectively, p=0.02). Given the lack of significant difference in the main outcome variables at baseline, data from the two sites were combined for the analysis.

All staff approached agreed to participate in the interviews. There were no significant differences between intervention and comparison sites at baseline, regarding district of origin, median age of respondent, gender, cadre of health worker, and working duration (Table 1). The proportion of participants who had received previous training (through the routine health system) on specific topics was similar in intervention versus comparison sites, except for three topics which had better coverage in comparison sites (Supplementary Figure 1). These were: ever trained on how to assess and support ART adherence for HIV positive women (78.6% in intervention sites versus 89.2% in comparison sites, p=0.01); ever trained about managing breastfeeding problems (76.5% in intervention sites and 86.2% in comparison sites, p=0.02); and received any information or training about the revised infant feeding policy (55.1% in intervention sites versus 67.4% in comparison sites p=0.02). At baseline, activities around breastfeeding counselling and management were similar between comparison and intervention sites in all respects except that comparison site participants reportedly spoke more frequently to HIV positive pregnant women about feeding than intervention participants (67% versus 71.6% spoke more than 1-3 times per month, p=0.04, data not shown).

For peer review only - http://bmjopen.bmj.com/site/about/guidelines.xhtml

Table 1: Characteristics of the participants in the intervention and comparison groups at baseline

Characteristic	Intervention group (n=289) (N (%))	Comparison group (n=131) (N (%))	p-value
**District:**			
- Tshwane	152 (52.6)	56 (42.8)	0.06
- Ugu	137 (47.4)	75 (57.3)	
**Age categories:**			
- 23 to 35 years	56 (19.4)	38 (29.7)	0.11
- 36 to 41 years	61 (21.2)	25 (19.5)	
- 42 to 46 years	53 (18.4)	26 (20.3)	
- 47 to 54 years	64 (22.2)	18 (14.1)	
- Over 54 years	54 (18.8)	21 (16.4)	
**Gender**			
- Female	267 (92.7)	118 (91.5)	0.66
- Male	21 (7.3)	11 (8.5)	
**Cadre of health worker**			
- Community level worker	84 (29.5)	52 (40,0)	0.05
- Trained health professional*	151 (53.0)	64 (49.2)	
- Enrolled nurse	50 (17.4)	14 (10.8)	
**Work experience in year (yr)/ years (yrs)**			
- Less than 1 yr	4 (1.4)	3 (2.3)	0.20
- 1 to <2 yrs	23 (8.0)	12 (9.3)	
- 2 to less than 5 yrs	36 (12.5)	18 (14.0)	
- 5 to less than 10 yrs	71 (24.7)	43 (33.3)	
- 10 yrs or more	154 (53.5)	53 (41.1)	

Abbreviations: *includes 68% nurses in the intervention arm and 58% nurses in comparison arm. This group also includes operation managers, dieticians, doctors and nutritionists.

In intervention sites, workshops were attended by 84-88% of participants interviewed at follow-up (Table 2).

Table 2: Attendance at workshops 1-3 measured at follow-up in intervention sites

	Attended workshop n	Attended catch-up n	Total attended n/N (%)
**Number of staff attending each workshop:**			
Group workshop 1	202	63	265/303 (87.5)
Group workshop 2	223	34	257/303 (84.8)
Workshop 3 (Clinical mentoring)	216	40	256/303 (84.5)
**Number of workshops attended:**			
	number	%	
No workshop	42	13.9	
1 workshop	8	2.6	
2 workshops	6	2.0	
All 3 workshops	247	81.5	
**Total**	**303**	**100**	

For peer review only - http://bmjopen.bmj.com/site/about/guidelines.xhtml

Effect of the intervention on health worker knowledge

At baseline, knowledge about key infant feeding statements or facts was similar between intervention and comparison sites, except for knowledge about soft porridge (Table 3). Although at baseline, more than 90% of intervention and comparison site participants knew that a baby under 4 months should not be given soft porridge if hungry, significantly more intervention site participants knew this recommendation (Table 3). The percentage of participants at baseline correctly answering the more difficult questions (on bottle sterilisation, storing expressed breastmilk, feeding HIV exposed infants) was low (Table 3). At follow-up significantly more intervention site participants correctly answered knowledge questions, regarding the leading cause of death in children under 5, the risk of formula feeding, duration of breastfeeding for HIV-negative mothers and women living with HIV, how to stop breastfeeding, complementary feeding, storing expressed breastmilk, feeding whilst at work, breastfeeding and viral suppression, mixed feeding in women living with HIV, adherence to ART and breastfeeding, breastfeeding difficulties in women living with HIV and managing women living with HIV who are breastfeeding, than comparison site participants (Table 3). The significant differences between intervention and comparison sites regarding soft porridge were not present at follow-up. Although improvements were seen in knowledge related to the risks of mixed feeding for women living with HIV, most health workers still provided incorrect responses at follow-up. At baseline, the mean number of correctly answered knowledge questions was 15.0 (68%) in comparison sites versus 15.2 (69%) in intervention sites, p=0.89 (Table 3). At follow-up the mean number was to 15.2 (69%) in comparison sites and 17.2 (78.2%) in intervention sites, p<0.001 (Table 3). For the two questions measuring knowledge about the 2017 change in infant feeding guidelines, namely, "Continued breastfeeding for 2 years is the recommended infant method in SA for ALL children, regardless of mother's HIV status" and "In South Africa, HIV-infected women who are breastfeeding should be supported to adhere to antiretroviral treatment and should introduce complementary foods at 6 months and be supported to continue breastfeeding for at least two years. (True)", there was a 36% improvement in knowledge in the intervention group at follow-up compared with a 13% increase in knowledge in the control group. For the second question there was a 15% increase in correct knowledge in the intervention group at follow-up while for the comparison group knowledge decreased from 89-81%. At follow-up, knowledge scores of participants who attended 3 workshops compared with knowledge scores of participants who attended less than 3 workshops was significantly better (p<0.001).

For peer review only - http://bmjopen.bmj.com/site/about/guidelines.xhtml

Table 3: Knowledge of health workers about breastfeeding in the intervention and comparison sites at baseline and follow-up

Knowledge statements	Number (%) with correct answers at BASELINE			Number (%) with correct answers at FOLLOW-UP		
	Intervention (n=289)	Comparison (n=131)	p-value *	Intervention (n=250)	Comparison (n=112)	p-value *
**Knowledge relating to updates in the HIV and Infant feeding guidelines**						
Significant improvements between intervention and comparison groups at follow-up						
Continued breastfeeding for 2 years is the recommended infant method in SA for ALL children, regardless of mother's HIV status (True)ǂ	190 (65.7)	91 (70.0)	0.39	224 (89.6)	88 (78.6)	<0.01
An HIV-positive mother who is virally suppressed on antiretroviral treatment should breastfeed her child rather than not breastfeed to improve the child's survival (True) ǂ	237 (82.0)	108 (83.1)	0.79	236 (94.4)	96 (85.7)	<0.01
A mother who has missed 6 tablets of Fixed Dose Combination ART in one month is considered to be poorly adherent and should stop breastfeeding immediately (False)** ǂ	181 (62.6)	89 (68.5)	0.25	201 (80.4)	72 (64.3)	<0.01
In South Africa, HIV-infected women who are breastfeeding should be supported to adhere to antiretroviral treatment and should introduce complementary foods at 6 months and be supported to continue breastfeeding for at least two years. (True) ǂ	245 (84.8)	116 (89.2)	0.220	244 (97.6)	91 (81.3)	<0.01
When an HIV-infected mother is ready to add complementary feeds she should stop breastfeeding rapidly over a 24-hour period (False) ǂ	214 (74.1)	103 (79.2)	0.25	217 (86.8)	86 (76.8)	<0.05
If a mother misses 2 doses of her ART in one month, she should be classified as a treatment failure (False)** ǂ	185 (64.0)	82 (63.1)	0.85	191 (76.4)	73 (65.2)	<0.05
Low levels of knowledge (<80%) at baseline in both groups - – no significant differences between intervention and comparison groups at follow-up [concept that this relates to]						
If an HIV exposed baby is receiving both breastmilk and formula milk, the mother should choose either breastfeeding or formula feeding if she is adherent to ART (False) ǂ [mixed feeding with formula and breastmilk]	69 (23.9)	29 (22.3)	0.71	75 (30.0)	28 (25.0)	0.33
A mother living with HIV and adherent to antiretroviral treatment cannot exclusively breastfeed her 4-month old infant because she is working. It is better for this mother to give formula during the day and breastfeed at night rather than giving no breast milk at all (True) ǂ [mixed feeding with formula and breastmilk]	22 (7.6)	14 (10.8)	0.29	40 (16.0)	14 (12.5)	0.38

For peer review only - http://bmjopen.bmj.com/site/about/guidelines.xhtml

High levels of knowledge (≥80%) at baseline in both groups – no significant differences between intervention and comparison groups at follow-up						
In South Africa, HIV-infected women who are breastfeeding should be supported to adhere to antiretroviral treatment and should be counselled and supported to exclusively breastfeed their infants for the first six months of life whilst maintaining an undetectable viral load (True)ǂ	281 (97.2)	123 (94.6)	0.18	242 (96.8)	109 (97.3)	0.79
Mothers living with HIV who are receiving antiretroviral treatment and are virally suppressed should be advised not to breastfeed their infants (False) ǂ	252 (87.2)	115 (88.5)	0.85	230 (92.0)	99 (88.4)	0.27
**General breastfeeding**						
Significant improvements between intervention and comparison groups at follow-up						
In South Africa, the leading cause of death amongst children under 5 is pneumonia (True)	189 (65.4)	82 (63.1)	0.65	230 (92.0)	75 (67.0)	<0.01
Giving any formula milk during the first six months of life increases the risk of death from diarrhoea and/or pneumonia (True)	246 (85.1)	104 (80.0)	0.17	232 (92.8)	95 (84.8)	0.02
It is safe to give the baby expressed breastmilk that has been kept outside the fridge for 8 hours (True)	106 (36.7)	43 (33.1)	0.48	120 (48.0)	38 (33.9)	<0.05
A mother who is working and giving formula milk should mix the milk herself and leave for the carer to give during the day (False)	218 (75.4)	94 (72.3)	0.50	189 (75.6)	68 (60.7)	<0.01
Low levels of knowledge (<80%) at baseline in both groups - – no significant differences between intervention and comparison groups at follow-up						
When sterilising feeding bottles cover the bottles with water in a saucepan and place on the heat. As soon as the water boils remove from heat and do not leave the bottles in the water until completely cool (False)	64 (22.2)	27 (20.8)	0.75	53 (21.2)	25 (22.3)	0.81
High levels of knowledge (≥80%) at baseline in both groups – no significant differences between intervention and comparison groups at follow-up						
Exclusive breastfeeding is the recommended infant feeding method for ALL infants aged 0-6 months in SA, regardless of mother's HIV status (True)	271 (93.8)	118 (90.8)	0.27	234 (93.6)	102 (91.1)	0.32
A baby under 4 months should be given soft porridge once he/she seems hungry (False)	284 (98.3)	124 (95.4)	0.09	247 (98.8)	108 (96.4)	0.13
Giving a baby expressed breastmilk is not as good as breastfeeding (False)	234 (81.0)	106 (81.5)	0.89	218 (87.2)	96 (85.7)	0.70
There are long term health benefits of breastfeeding for mother and child that last beyond the breastfeeding period (True)	264 (91.4)	116 (89.2)	0.49	232 (92.8)	100 (89.3)	0.26

For peer review only - http://bmjopen.bmj.com/site/about/guidelines.xhtml

Breastfeeding and HIV						
Significant improvements between intervention and comparison groups at follow-up						
An HIV-positive mother who has cracked nipples should continue to breastfeed unless they are bleeding (True)	143 (49.5)	64 (49.2)	0.96	187 (74.8)	59 (52.7)	<0.01
If a baby has a positive PCR (HIV test) at birth the mother should stop breastfeeding if this is affordable and feasible in her situation (False)**	224 (81.0)	100 (76.9)	0.90	214 (85.6)	82 (73.2)	<0.01
High levels of knowledge (<80%) at baseline in both groups – no significant differences between intervention and comparison groups at follow-up						
An HIV-exposed baby who is exclusively breastfeeding should be given some water when the weather is very hot (False)**	270 (93.4)	122 (93.9)	0.87	239 (95.6)	105 (93.8)	0.45
**Mean knowledge score (standard deviation) out of 22**	15.2 (2.6)	15.0 (3.1)	0.61*	17.2 (2.1)	15.2 (2.8)	<0.01

**The Statement is false; thus, the scales were inverted during data analysis. ǂ - these questions measure the change in knowledge relating to the South African Department of Health June 2017 circular and the WHO 2016 updated HIV and Infant feeding guidelines
*independent t-test comparing intervention and comparison sites at the relevant time point.
Note: the tables displays numbers with correct knowledge

Effect of the intervention on attitudes

At baseline, intervention and comparison sites were similar in HW attitudes except for attitudes towards feeding a crying baby and expressing breastmilk, which were significantly better in intervention sites (Supplementary Table 1). At follow-up attitudes to breastfeeding and HIV were significantly better in the intervention group for 13 of the 21 questions and the mean attitude score towards breastfeeding was significantly better in intervention sites (p<0.001) (Supplementary Table 1). At follow-up HW in the intervention group were significantly less confused about what to tell women living with HIV about infant feeding mothers about HIV and infant feeding. Methods 1 and 2 yielded the same results, except for the effect of baseline attitude score. Thus, in Table 4 below, we only show results for Methods 1 and 3. Controlling for other variables, post intervention attitude was significantly better in intervention, compared with comparison, sites (Table 4). Using Methods 1, attitude at follow-up was 5.4 points higher in the intervention group than the comparison group; Method 3 analysis showed a significant 5.1-point higher score in the intervention compared with the comparison group Using Method 1, being an enrolled nurse, and being in the youngest (36-41 years) or oldest (>54 years) age group was associated with a significantly lower attitude score. Using Methods 3, trained health professionals had a significantly higher attitude score at follow-up (, p<0.05).

For peer review only - http://bmjopen.bmj.com/site/about/guidelines.xhtml

Table 4: Adjusted effect of the intervention on health worker attitude score using different methods (Effect estimate and 95% confidence interval (CI))

Variable	Method 1		Method 3	
	Effect estimate	95% CI	Effect estimate	95% CI
**Attitude score at baseline**	**0.5**	**0.3; 0.7***	N/A	N/A
**Intervention**	**5.4**	**3.9; 6.9***	**5.1**	**2.1; 8.1***
**Follow-up Period**	N/A	N/A	1.8	0.2-3.4*
**Professional role: vs community level**				
- Trained health professional	1.6	-0.05; 3.2	**4.8**	**2.8; 6.7***
- Enrolled nurse	**-2.4**	**-5.0; -0.2***	0.9	-1.4; 3.2
**Ugu District vs Tshwane District**	-0.83	-2.2; 0.5	-1.4	-3.1; 0.2
**Age category: vs 23-35 yrs**				
- 36 to 41 yrs	**-2.8**	**-5.4; -0.2***	-1.8	-4.1; 0.6
- 42 to 46 yrs	-0.9	-3.3; 1.5	-0.2	-2.5; 2.2
- 47 to 54 yrs	0.5	-2.0; 2.9	-1.2	-3.1; 0.8
- over 54 yrs	**-3.3**	**-5.7; -1.0***	-2.2	-4.8; 0.3
**Work experience <5 yrs vs ≥5yrs**	-0.3	-2.5; 1.9	-1.3	-3.4; 0.8
**Received training or information at work about the revised policy**	0.5	-1.4; 2.3	1.7	0.1-3.24
**Received any training about managing common breastfeeding problems?**	0.3	-2.5; 3.0	3.2	0.9-5.5
**Ever received any training about how to assess and support ART adherence for HIV positive women?**	-0.1	-2.2; 2.0	1.6	-0.5-3.8

*p<0.05 N/A: not applicable. Note: All analyses are adjusted for clustering

For peer review only

Effect of the intervention on confidence

At baseline there was no difference in the percentage of participants in the intervention and control sites who were confident or very confident in counselling mothers on HIV / infant feeding (Supplementary Table 2). However, at follow-up HWs from intervention sites were significantly more confident in counselling an HIV positive women about HIV and infant feeding, advising HIV positive women about return to school/work, advising HIV positive mothers to continue breastfeeding for two years, assessing ART adherence in HIV positive mothers, and advising HIV positive mothers about breastfeeding with cracked nipples (Supplementary table 2). Confidence had not shifted about how to stop breastfeeding, identifying when a mother is not ART adherent and managing poor adherence, advising on formula feeding and counselling that a shorter breastfeeding duration is better than no breastfeeding. The mean confidence score at follow-up was significantly higher in the intervention compared with the comparison sites at follow-up (p=0.05) (Supplementary Table 2). Methods 1 and 2 yielded the same results, except for the effect of baseline confidence score. Thus, in Table 5 below, we only show results for Methods 1 and 3. Controlling for other variables, post intervention confidence was significantly better in intervention, compared with comparison, sites; however this was only statistically significant

For peer review only - http://bmjopen.bmj.com/site/about/guidelines.xhtml

under Method 1.. Our analysis demonstrated that, controlling for other factors, being a trained health professional significantly increased confidence score by 3.1 (Method 1) or 3.7 (Method 3). Additionally, Method 3 demonstrated that, controlling for other factors, working for less than 5 years significantly reduced the confidence score.

Table 5: Adjusted effect of the intervention on health worker confidence scores, using different multivariable analysis methods (Effect estimate and 95% confidence interval (CI))

Variable	Method 1		Method 3	
	Effect estimate	95% CI	Effect estimate	95% CI
**Confidence score at baseline**	**0.4**	**0.3; 0.6***	N/A	N/A
**Intervention**	**2.4**	**0.3; 4.5***	1.5	-2.2; 5.1
**Follow-up time**	N/A	N/A	0.5	-1.5; 2.5
**Cadre of heath professional: vs community level**				
- Trained health professional	**3.1**	**0.3; 5.9***	**3.7**	**1.5; 5.9***
- Enrolled nurse	-0.8	-4.3; 2.7	-0.7	-3.1; 1.6
**Ugu District vs Tshwane District**	0.00	-2.1; 2.1	-1.	-3.2; 1.2
**Age category vs 23-35 yrs**				
- 36 to 41 yrs	-1.0	-3.7; 1.6	-0.1	-2.7; 2.5
- 42 to 46 yrs	0.3	-2.9; 3.4	0.4	-1.2; 2.9
- 47 to 54 yrs	1.4	-0.7; 3.5	-1.3	-3.4; 0.8
- over 54 yrs	-2.5	-5.7; 0.7	-0.9	-4.0; 2.2
**Work experience <5 yrs vs ≥5 yrs**	-0.5	-3.4; 2.4	**-1.9**	**-3.7; -0.2***
**Received training or information at work about the revised policy**	0.05	-1.5; 1.6	1.7	-0.3; 3.6
**Received any training about managing common breastfeeding problems?**	-0.6	-3.2; 2.1	1.8	-0.5; 4.1
**Ever received any training about how to assess and support ART adherence for HIV positive women?**	0.8	-2.1; 3.7	5.7	3.5; 7.9

*p<0.005 N/A= not applicable yrs= years. NB: All analyses are adjusted for clustering

For peer review only

Dose response analysis

We also conducted a dose-response analysis to assess whether or not the mentored health workers responded differently according to the number of workshops attended. The number of workshops grouped into 0, 1 or 2 and 3. Even though post intervention attitude and confidence scores as well as their increases were higher in the higher workshop attendance participants, none had statistically significant dose-response effect (p-value >0.05, data not shown)

For peer review only - http://bmjopen.bmj.com/site/about/guidelines.xhtml

Discussion

We demonstrate that a participatory, side-by side, team-based mentoring approach to disseminating updated HIV and infant feeding guidelines was associated with an improvement in health workers' attitudes. when controlling for other factors. There was also a significant improvement in mean knowledge score between intervention and control sites at follow-up. However, we were not successful in shifting knowledge and attitudes about mixed feeding (breastmilk and formula milk) and health workers at the end of the study were not confident in advising that a shorter duration of breastfeeding is better than no breastfeeding at all. This demonstrates the success of at least 15 years of frequent publicity about the dangers of mixed feeding in the context of HIV and no ART, given that the two seminal papers on feeding practices and HIV were led by South African researchers.[35 36] Concerted communication? efforts are needed to highlight the acceptability of mixed feeding in the context of ART and maternal viral load suppression to facilitate a shift in knowledge about mixed feeding. Although some individual attitude and confidence items did not change, or only changed marginally, the overall analyses demonstrated an improvement in follow-up attitude and confidence scores. However, confidence in the intervention group was still low and health workers performed poorly on some of the more difficult confidence questions such as confidence with counselling when a mother is not ART adherent, managing high viral loads during breastfeeding, explaining HIV transmission risks to a mother with a high viral load, assisting mothers with HIV to safely formula feed and advising that some breastfeeding is better than no breastfeeding. The complexity of changing health workers' attitudes and confidence towards breastfeeding has been documented repeatedly in many African settings including South Africa.[11 25 37-39] We hypothesise that poor performance on some of the individual items or on the overall confidence score may be attributed to the short duration of the intervention. An alternative hypothesis is that HW's low confidence, around topics like non-adherence and high viral load, reflect more complex dynamics that are not easily addressed through counselling / mentoring interventions.[39] In fact a study from South Africa demonstrated how health workers personal beliefs affect their ability to provide supportive counselling.[11]

There is evidence that in-service training, supervision and follow-up improves the knowledge, skills and practices of health workers managing childhood undernutrition, and can improve health worker job satisfaction and motivation, but no data exist on how to improve health worker knowledge, skills and confidence in the tricky area of HIV and infant feeding.[40-42] For training / supervision interventions, implementation challenges include inadequately trained or shortages of supervisors, inappropriate job aids for follow-up, and poor alignment between community views/ practice and health programmes.[43] Our approach attempted to circumvent these challenges by using a low technology, mentorship model for skills development at clinic level. At the outset of the intervention we acknowledged that health workers were members of their community: we discussed their fears and beliefs, and then introduced facts and evidence to extend their knowledge, change their attitudes and increase their confidence to implement updated guidelines on HIV and infant feeding. Thus, we aimed to change inherent, deep seated beliefs and attitudes that are sustained in the absence of outside supervision.

We used a side-by-side mentorship approach, as reviewed by Schwerdte et.al. to conduct team-based mentoring to empower health workers.[44] A team-based approach allowed collaborative

For peer review only - http://bmjopen.bmj.com/site/about/guidelines.xhtml

learning between different cadres of health workers, facilitating any future change in practice. In accordance with Dee Fink's theory, a participatory mentorship approach allows participants to develop foundational knowledge, apply skills, integrate ideas, develop new feelings/ interests and values and learn how to learn.[26] Our experience suggests that such an approach allowed discussion of participants' attitudes towards performing the behaviour, beliefs about whether critical, important people will approve of the behaviour (subjective norms), and about their likelihood of successfully implementing the behaviour.[28 29] Our findings corroborate a scoping review which demonstrated that mentorship improves certain quality of care outcomes [44]; in our study it improved knowledge, attitudes and confidence. However, only four studies were included in this scoping review, and the nature of the mentorship varied from video-conferencing to monthly, six-weekly or annual visits interspersed with other contact forums, conducted over one day to an entire week. A list of desirable features of mentorship interventions, include at least one dedicated mentor per facility, ensuring an adequate mentor: mentee ratio so that all staff can be supported, forming meaningful relationships between mentors and mentees, ensuring cultural congruency between mentee and mentor, and using mentors for protocol-driven programmes, such as IMCI or HIV.[44] Our intervention related to HIV and infant feeding guidelines, was low cost and low technology (one mentor working with pen, flip chart and paper in the health facility), and was implemented by a dedicated mentor from the same cultural background as the mentees. She provided onsite support during the workshops, which lasted approximately one hour, and additional support through a WhatsApp messaging group.

There is an ongoing heath worker crisis in resource limited settings, including maldistribution of staff, an imbalance in skills mix, increasingly complicated health programmes and complicated socio-cultural-political-economic environments. Against this background, questions arise about the feasibility of an on-site mentorship approach to guideline dissemination amongst health workers, and an on-site peer-peer mentorship approach between women living with HIV to supporting mothers with infant feeding. In this study we chose to focus specifically on an onsite mentorship approach to guideline dissemination amongst health workers. We argue that strengthening investment in on-site mentorship rather than off-site training, may be a cost-saving approach. In our setting, all clinics receive regular visits from district primary health care (PHC) supervisors, but they mostly focus on administration and clinic management matters. These supervisors, as well as existing district PHC trainers, could be capacitated to provide clinical mentoring for health workers in the clinics they oversee. Our model of team-based learning and mentoring can be used for on-site mentoring, and avoids accommodation and travel costs, and absence from work that off-site training requires.

Our study had several limitations: We purposively selected districts for inclusion. We could not control for previous breastfeeding experience of health workers as we did not gather these data. The study tools were piloted before finalisation, but no factor analyses or validation exercises were conducted. The follow-up evaluation was conducted 3 months after the intervention; thus, we were only able to measure short term benefits. Additionally, we did not co-design the intervention with women living with HIV, did not measure the effect of improved knowledge, attitudes and confidence on health workers' counselling practices and on mothers' infant feeding practices and could not tease out whether the relationship between number of workshops and outcomes was due to staff motivation (more motivated staff attended more workshops) or the workshops themselves.

For peer review only - http://bmjopen.bmj.com/site/about/guidelines.xhtml

Co-designing the intervention with mothers living with HIV may have resulted in a different intervention and results, this needs to be considered in future work. Our study's strengths are that the design was quasi-experimental, measuring not only knowledge, but also attitudes and confidence. Additionally, results are robust as three different analytical methods yielded congruent results.

Conclusion: We demonstrated improved knowledge, attitudes and confidence of health workers following a participatory mentorship approach to HIV and infant feeding guideline dissemination compared with a standard approach. More research is needed to better understand how to change health workers' counselling practices, and whether this changes mothers' feeding practices.

Authors contributions:

AG: Study conceptualization and tool development, protocol writing including intervention development, oversight of sampling and field work, writing of the first draft of this manuscript, receiving and incorporating co-author comments, finalization of the paper
TD: Study conceptualization and tool development, protocol writing including intervention development, set up the sample frame and sampling, contributed to the manuscript, reviewed and approved the final version of the manuscript
SM: Led the statistical components of the protocol; provided overall oversight on the statistical analysis, contributed to the manuscript, reviewed and approved the final version of the manuscript
TN: Performed the work on the statistical components of the protocol, under SM's guidance; provided data analysis under SM's guidance, contributed to the manuscript, reviewed and approved the final version of the manuscript
LH: Contributed to study conceptualization and tool development, protocol writing including intervention development; was overall Project Manager; established, managed and cleaned the database; contributed to the manuscript, reviewed and approved the final version of the manuscript
VJ: Provided guidance on intervention development. Contributed to the manuscript, reviewed and approved the final version of the manuscript
IMSE: Contributed to study conceptualization and tool development, protocol writing including intervention development; contributed to the manuscript, reviewed and approved the final version of the manuscript
UF: Contributed to study conceptualization, assisted with district level buy-in in Tshwane District, provided information on how routine dissemination of updated infant feeding guidelines; contributed to the manuscript, reviewed and approved the final version of the manuscript
AD: Contributed to study conceptualization and tool development; contributed to the manuscript, reviewed and approved the final version of the manuscript
NR: Contributed to study conceptualization; contributed to the manuscript, reviewed and approved the final version of the manuscript
MK: Contributed to study conceptualization and tool development; contributed to the manuscript, reviewed and approved the final version of the manuscript
DS: Contributed to study conceptualization and tool development; contributed to the manuscript, reviewed and approved the final version of the manuscript

For peer review only - http://bmjopen.bmj.com/site/about/guidelines.xhtml

For peer review only

SK: Contributed to study conceptualization, assisted with national level buy-in, provided information on how routine dissemination of updated infant feeding guidelines; contributed to the manuscript, reviewed and approved the final version of the manuscript
TT: Contributed to study conceptualization and tool development; contributed to the manuscript, reviewed and approved the final version of the manuscript
CH: Study conceptualization, protocol writing including intervention development, high level oversight of study implementation, contributed to the manuscript, reviewed and approved the final version of the manuscript

Data sharing statement: Data can be obtained by e-mailing the corresponding author, and upon reasonable request

Figure legends:

Figure 1: Study districts: Tshwane District in Gauteng Province and Ugu District in KwaZulu-Natal Province of South Africa

Figure 2: Theoretical frameworks which informed the development of the intervention

Figure 3: Study population at baseline and follow-up for intervention and comparison sites

Supplementary Figure 1: Prior training reported by participants at baseline (intervention versus comparison groups)

For peer review only

References (please see clean document for revised reference list – it does not update on the track changes version)

1. Victora C, Bahl R, Barros A, et al. Breastfeeding in the 21st century: epidemiology, mechanisms, and lifelong effect. The Lancet 2016;**387**(10017):475-90
2. Black RE, Allen LH, Bhutta ZA, et al. Maternal and child undernutrition: global and regional exposures and health consequences. Lancet. 2008.;**37**(9608):243-60
3. National Department of Health. Policy and Guidelines for the Implementation of the PMTCT programme. 2008. Available from http://www.info.gov.za/view/DownloadFileAction?id=77877. Accessed 11 June 2020.
4. National Department of Health. The South African Antiretroviral Treatment Guidelines 2013: PMTCT Guidelines Revised March 2013. 2013. Available from https://sahivsoc.org/Files/2013%20ART%20Treatment%20Guidelines%20Final%2025%20March%202013%20corrected.pdf. Accessed 11 June 2020.
5. National Department of Health. National conslidated guidelines for the prevention of mother to child transmission of HIV (PMTCT) and the management of HIV in children, adolescents and adults. 2015. Available from https://www.health-e.org.za/2015/07/02/guidelines-national-consolidated-guidelines-for-pmtct-and-the-management-of-hiv-in-children-adolescents-and-adults/. Accessed 11 June 2020.
6. National Department of Health. Guideline for the Prevention of Mother to Child Transmission of Communicable Infections. 2019. Available from https://www.knowledgehub.org.za/system/files/elibdownloads/2019-10/PMTCT%20Guideline%2028%20October%20signed.pdf. Accessed 11 June 2020.
7. National Department of Health and SANAC. Clinical Guidelines: PMTCT (Prevention of Mother-to-Child Transmission). 2010. Available from https://www.fidssa.co.za/Content/Documents/PMTCT_Guidelines.pdf. Accessed 11 June 2020.
8. National Department of Health Breastfeeding Summit. The Tshwane Declaration of Support for breastfeeding in South Africa. S Afr J Clin Nutr 2011;**24**(4):214-16
9. World Health Organisation, UNICEF. 2016 Guideline Update on HIV and Infant Feeding. 2016. Available from https://www.who.int/nutrition/publications/hivaids/guideline_hiv_infantfeeding_2016/en. Accessed 11 June 2020.
10. Martin-Wiesner, P. A policy-friendly environment for breastfeeding A review of South Africa's progress in systematising its international and national responsibilities to protect, promote and support breastfeeding. In: University of Witwatersrand: DST-NRF Centre of Excellent in Human Development, 2018. Available from https://www.wits.ac.za/media/wits-university/research/coe-human/documents/Breastfeeding%20policy%20review.pdf. Accessed 11 June 2020..
11. Nieuwoudt S, Manderson L. Frontline health workers and exclusive breastfeeding guidelines in an HIV endemic South African community: a qualitative exploration of policy translation. International Breastfeeding Journal (2018) 13:20 2018;**13**(20):https://doi.org/10.1186/s13006-018-0164-y
12. Doherty T, Chopra M, Nkonki L, Jackson d, Greiner T. Effect of the HIV epidemic on infant feeding in South Africa: "When they see me coming with the tins they laugh at me". Bull of the World Health Organ. 2006;**84**(2):90-96

For peer review only - http://bmjopen.bmj.com/site/about/guidelines.xhtml

13. Doherty T, Horwood C, Haskins L, et al. Breastfeeding advice for reality: Women's perspectives on primary care support in South Africa. . Maternal & Child Nutrition 2019;**16**(1):e12877 DOI: 10.1111/mcn.77.
14. Seidel G, Sewpaul V, Dano B. Experiences of breastfeeding and vulnerability among a group of HIV-positive women in Durban, South Africa. Health Policy Plan 2000;**15**:24-33
15. McCormack L, Sheridan S, Lewis M, et al. Communication and dissemination strategies to facilitate the use of health-related evidence. Secondary Communication and dissemination strategies to facilitate the use of health-related evidence. 2013. https://www.ncbi.nlm.nih.gov/books/NBK179104/pdf/Bookshelf_NBK179104.pdf.
16. Academy of Nutrition and Dietetics, Handu D, Acosta A, et al. Effectiveness of interventions to promote exclusive breastfeeding in women living with HIV that are on antiretroviral therapy living in areas that promote exclusive breastfeeding due to limited resources for safe replacement feeding. 2016. Available from https://www.who.int/maternal_child_adolescent/documents/hiv-infant-feeding-2016-ax2-breastfeeding-promotion_systematic-review.pdf?ua=1. Accessed 10 June 2020.
17. World Health Organization, United Nations Children's Fund. Guideline: updates on HIV and infant feeding: the duration of breastfeeding, and support from health services to improve feeding practices among mothers living with HIV. Geneva, 2016. Available from http://apps.who.int/iris/bitstream/handle/10665/246260/9789241549707-eng.pdf;sequence=1. Accessed 9 June 2020.
18. Barker M, Baird J, Lawrence W, et al. The Southampton Initiative for Health: a complex intervention to improve the diets and increase the physical activity levels of women from disadvantaged communities. . J Health Psychol 2011;**16**:178-91
19. Baird J, Jarman M, Lawrence W, al. e. The effect of a behaviour change intervention on the diets and physical activity levels of women attending Sure Start Children's Centres: results from a complex public health intervention. . BMJ Open 2014;**4:e005290.**
20. Heerink F, Krumeich A, Feron F, A. G. We are the advocates for the babies' - understanding interactions between patients and health care providers during the prevention of mother-to-child transmission of HIV in South Africa: a qualitative study. . Global Health Action 2019.;**12**:DOI: 10.1080/16549716.2019.1630100
21. Hector D, Webb K, King L. Factors affecting breastfeeding practices. Applying a conceptual framework. New South Wales Public Health Bulletin 2005:https://www.researchgate.net/profile/Lesley_King2/publication/7654972_Factors_affecting_breastfeeding_practices_Applying_a_conceptual_framework/links/004635254b6bd3b8d7000000/Factors-affecting-breastfeeding-practices-Applying-a-conceptual-framework.pdf?origin=publication_detail
22. Nieuwoudt S, Ngandu C, Manderson L, Norris S. Exclusive breastfeeding policy, practice and influences in South Africa, 1980 to 2018: A mixed-methods systematic review. PLoS One. 2019; 14(10): e0224029. 2019;**14**(10):doi: 10.1371/journal.pone.0224029
23. Janse van Rensburg L, Nel R, Walsh CM. Knowledge, opinions and practices of healthcare workers related to infant feeding in the context of HIV. Health SA Gesondheid 2016;**21**:129-36 doi: http://dx.doi.org/10.1016/j.hsag.2015.12.001[published Online First: Epub Date]|.
24. Eldridge S, Ashby D, Kerry S. Sample size for cluster randomized trials: effect of coefficient of variation of cluster size and analysis method. International Journal of Epidemiology 2006;**35**(1292-1300):doi:10.1093/ije/dyl129

For peer review only - http://bmjopen.bmj.com/site/about/guidelines.xhtml

25. Horwood C, Haskins L, Goga A, et al. An educational intervention to update health workers about HIV and infant feeding. . Maternal & Child Nutrition. 2019:https://doi.org/10.1111/mcn.12922
26. Pariyo GW, Gouws E, Bryce J, Burnham for the Uganda IMCI Impact Study Team,. Improving facility-based care for sick children in Uganda: training is not enough. Health Policy Plan 2005;**20**(Suppl_1):i58-i68
27. Dee Fink L. A self-directed guide to designing courses for significant learning. Secondary A self-directed guide to designing courses for significant learning 2003. https://www.deefinkandassociates.com/GuidetoCourseDesignAug05.pdf.
28. Ajzen I. The theory of planned behavior. Organizational Behavior and Human Decision Processes 1991;**50**(2):179-211 doi: http://dx.doi.org/10.1016/0749-5978(91)90020-T[published Online First: Epub Date]|.
29. Ajzen I. Perceived Behavioral Control, Self-Efficacy, Locus of Control, and the Theory of Planned Behavior1. Journal of Applied Social Psychology 2002;**32**(4):665-83 doi: 10.1111/j.1559-1816.2002.tb00236.x[published Online First: Epub Date]|.
30. World Health Organization, UNICEF. Breastfeeding Counselling: A Training Course. Geneva, 1993. Available from https://www.who.int/maternal_child_adolescent/documents/who_cdr_93_3/en/. Accessed 10 June 2020.
31. World Healh Organization, UNICEF. HIV and Infant Feeding Counselling: A Training Course. Geneva, 2000. Available from https://apps.who.int/iris/bitstream/handle/10665/66194/WHO_FCH_CAH_00.4.pdf;sequence=5. Accessed 11 June 2020
32. Doherty T, Sanders D, Goga A, Jackson D. Implications of the new WHO Guidelines on HIV and infant feeding for child survival in South Africa. . Bull World Health Organ. 2011;**89**:62-67
33. Goga A, Doherty T, Jackson D, et al. Infant feeding practices at routine PMTCT sites, South Africa: Results of a prospective observational study amongst HIV exposed and unexposed infants - birth to 9 months. Int Breastfeeding J. 2012;**7**(4):doi:10.1186/746-4358-7-4.
34. O'Connell N, Dai L, Jiang Y, Speiser J, Ward R, et al. Methods for Analysis of Pre-Post Data in Clinical Research: A Comparison of Five Common Methods. . J Biom Biostat 2017;**8**(334):doi:10.4172/2155-6180.1000334
35. Coovadia H, Rollins N, Bland R, et a. Mother-to-child transmission of HIV-1 infection during exclusive breastfeeding in the first 6 months of life: an intervention cohort study. . Lancet 2007;**369**:1107-16
36. Coutsoudis A, Pillay K, Kuhn L, et a. Method of feeding and transmission of HIV-1 from mothers to children by 15 months of age: prospective cohort study from Durban, South Africa. . AIDS 2001;**15**:379-8737. Desclaux A, Alfieri C. Counseling and choosing between infant-feeding options: overall limits and local interpretations by health care providers and women living with HIV in resource-poor countries (Burkina Faso, Cambodia, Cameroon). . Soc Sci Med. 2009;69:821–9. 2009;**69**:821-9
38. Tuthill E, Chan J, Butler L. Challenges faced by healthcare providers offering infant feeding counseling to HIV-positive women in sub-Saharan Africa: A review of current research. AIDS Care. 2015;**27**(1):17-24

For peer review only - http://bmjopen.bmj.com/site/about/guidelines.xhtml

39. Buskens I, Jaffe A. Demotivating infant feeding counselling encounters in southern Africa: do counsellors need more or different training? . AIDS Care. 2008;20(3):337–45. 2008;**20**(3):337-45
40. Sunguya B, Poudel K, Mlunde L, Urassa D, Yasuoka J, Jimba M. Nutrition Training Improves Health Workers' Nutrition Knowledge and Competence to Manage Child Undernutrition: A Systematic Review. Frontiers in public health 2013:10.3389/fpubh.2013.00037
41. Bailey C, Blake C, Schriver M, Cubaka V, Thomas T, Martin Hilber A. A systematic review of supportive supervision as a strategy to improve primary healthcare services in Sub-Saharan Africa. Int J Gynaecol Obstet. 2016;**132**(1):117-25. doi: 10.1016/j.ijgo.2015.10.004.
42. World Health Organisation. Management of Childhood Illness in Developing Countries: Rationale for an integrated approach. 1999. Available from http://www.who.int/child_adolescent_health/documents/chs_cah_98_1a/en/. Accessed 11 June 2020.
43. Goga A, Muhe L. Global Challenges with Scale-Up of the Integrated Management of Childhood Illness Strategy: Results of a multi-country Survey. . BMC Public Health. 2011;**11**(503):doi: 10.1186/471-2458-11-503
44. Schwerdte P, Morphet, Hall H. A scoping review of mentorship of health personnel to improve the quality of health care i low and middle-income countries. Globalisation and Health 2017;**13**(77):doi 10.1186/s12992-017-0301-1

For peer review only

Figure 1: Study districts: Tshwane Metropolitan Municipality in Gauteng Province and Ugu District in KwaZulu-Natal Province of South Africa

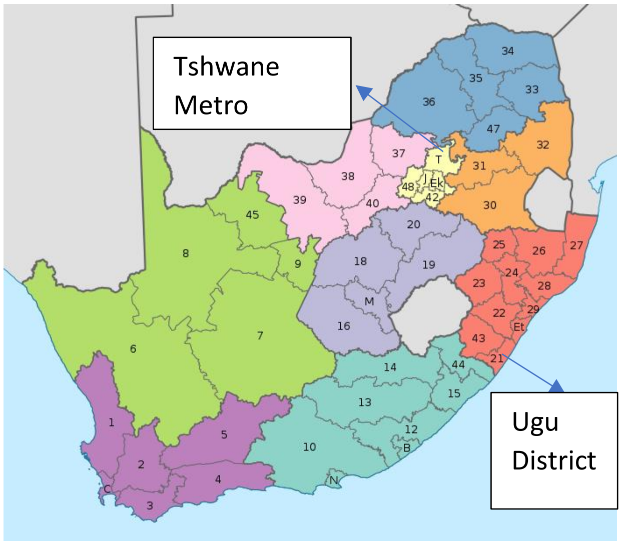

For peer review only

Figure 2: Theoretical frameworks which informed the development of the intervention

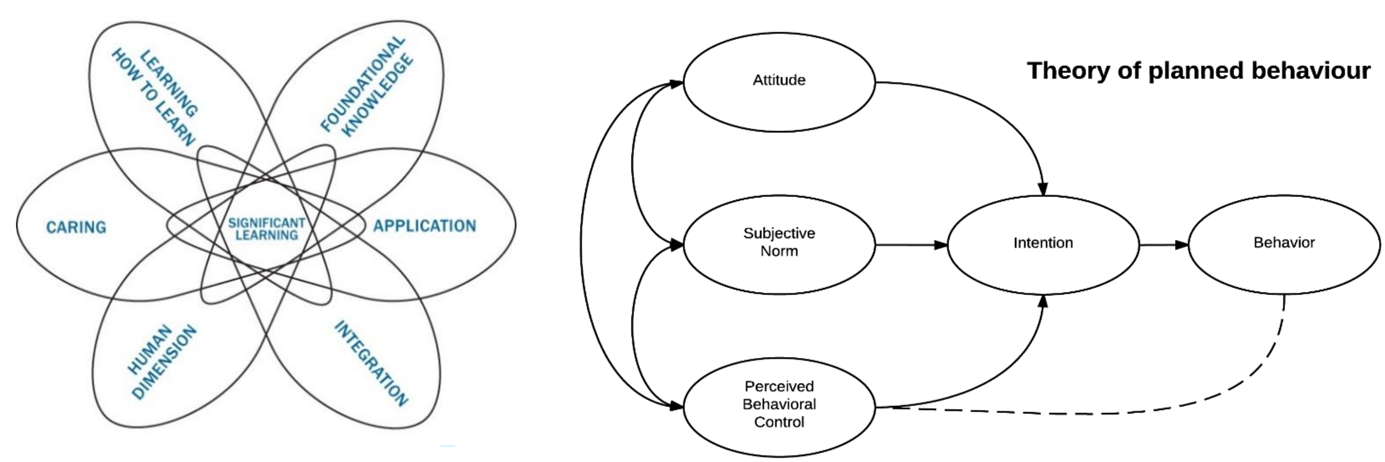

Dee Fink's six-part taxonomy for significant learning

For peer review only

Figure 3: Study population at baseline and follow-up for intervention and comparison sites

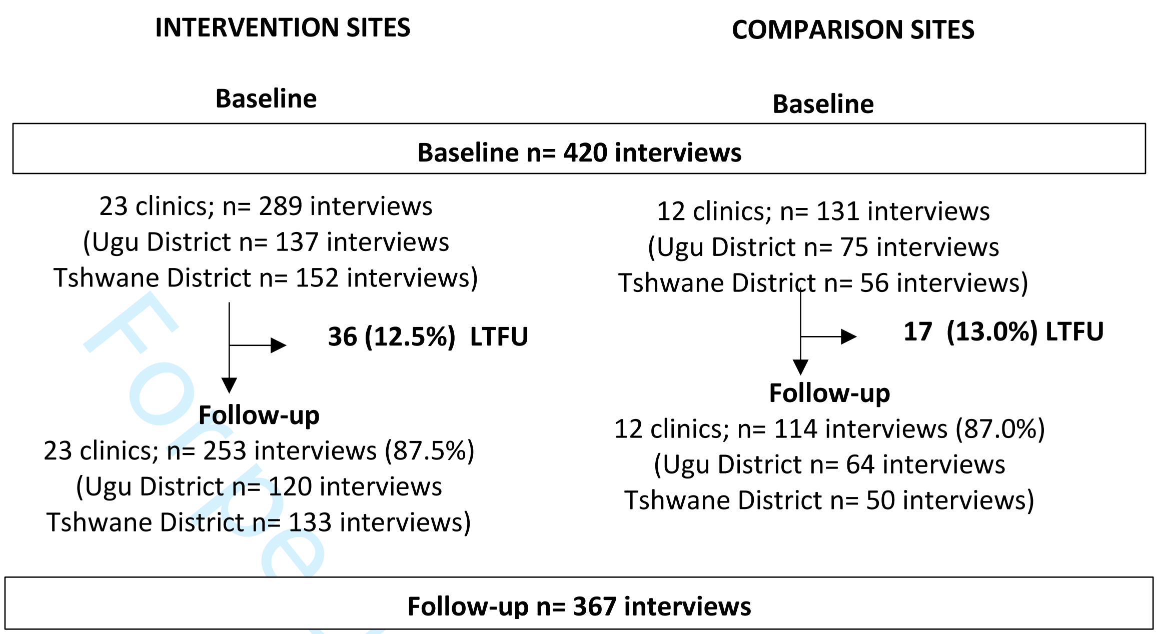

LTFU: lost to follow up

For peer review only

Supplementary Figure 1: Prior training reported by participants at baseline (intervention versus comparison groups)

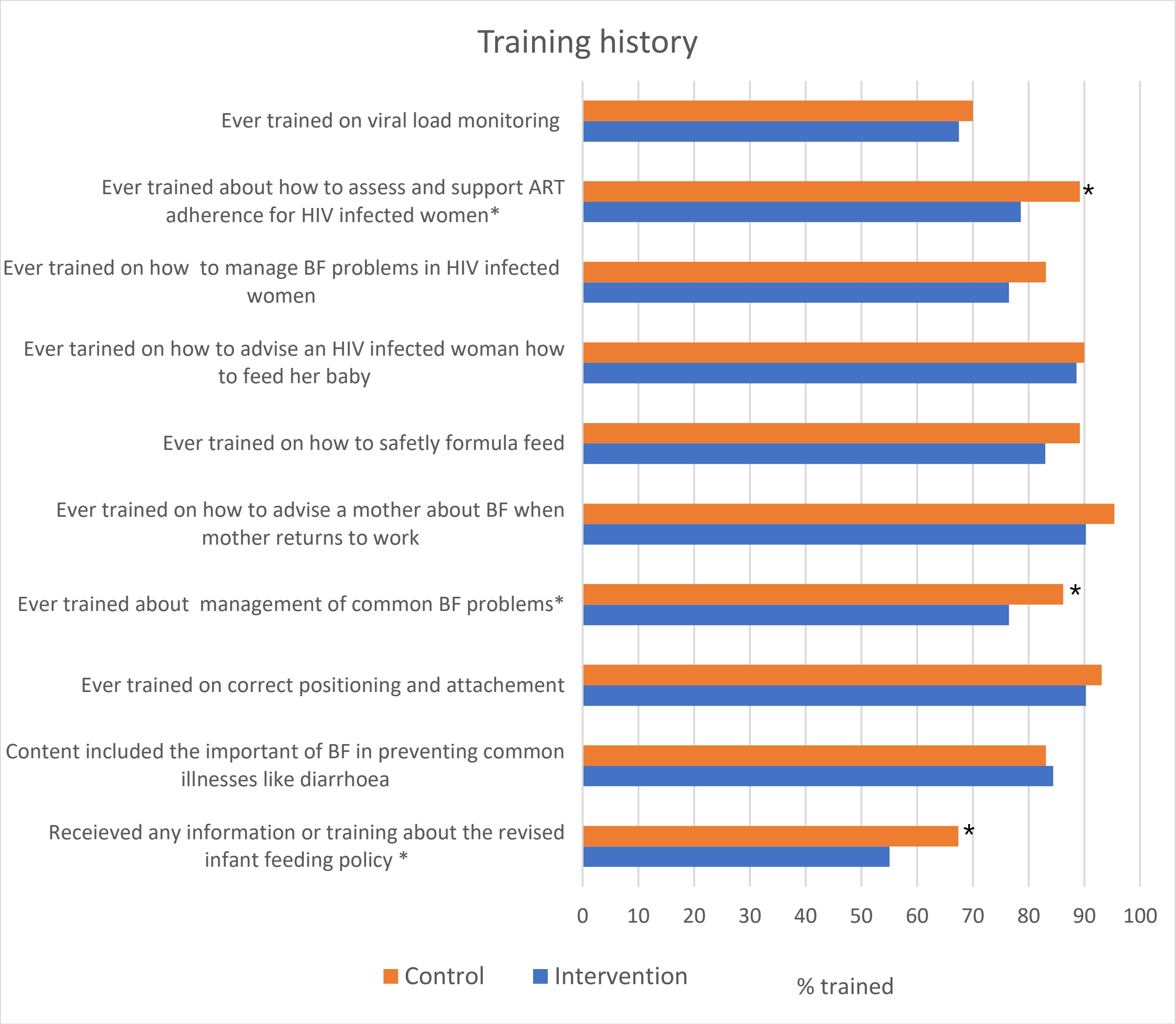

*p<0.05

Abbreviations: ART= antiretroviral therapy; BF= breastfeeding

Supplementary Table 1: Attitude scores of health workers at baseline and follow-up

Attitude statements	Number (%) who agreed or strongly agreed with the statement at baseline			Number (%) who agreed or strongly agreed with the statement at follow-up		
	Intervention (n=289)	Comparison (n=131)	p-value *	Intervention (n=252)	Comparison (n=114)	p-value*
Significant improvements between intervention and comparison groups at follow-up						
There have been so many changes to the infant feeding guidelines and breastfeeding guidelines however I am NOT confused about what to tell mothers who are HIV-infected about breastfeeding	155 (53.6)	74 (56.9)	059	175 (69.4)	65 (57.0)	0.02
For an HIV-exposed infant any breastfeeding is better than no breastfeeding at all, as long as the mother is virally suppressed and on antiretroviral therapy	189 (65.4)	79 (61.2)	0.31	196 (77.8)	74 (64.9)	0.01
When a baby cries all the time it is NOT usually because the baby is hungry and needs more food than just breastmilk	260 (90.0)	107 (83.0)	0.02	230 (91.3)	91(79.8)	<0.01
Exclusive breastfeeding in the first 6 months of life is the best choice for all mothers and babies in South Africa	256 (88.6)	114 (88.4)	0.65	239 (94.8)	98 (86.0)	<0.01
The benefits of breastfeeding for protecting children from illness such as diarrhoea and pneumonia outweighs the risk of acquiring HIV if the mother is on antiretroviral treatment	224 (77.5)	93 (72.1)	0.15	218 (86.5)	87 (76.3)	0.02
I feel that an HIV-infected mother who has not disclosed to her partner is NOT at high risk of non-adherence to ART and should NOT stop breastfeeding as soon as possible	128 (44.3)	51 (39.5)	0.30	137 (54.4)	46 (40.4)	0.01
I should support all mothers, regardless of HIV status, to continue breastfeeding until 2 years, as long as HIV-infected women are virally suppressed	237 (82.0)	101 (78.3)	0.24	237 (94.1)	87 (76.3)	<0.01
I should NOT advise an HIV-positive virally suppressed mother who has cracked and bleeding nipples to temporarily stop breastfeeding	80 (27.7)	40 (31.3)	0.55	97 (38.5)	24 (21.1)	<0.01
Formula feeding is NOT the best choice for mothers living in good socio-economic circumstances who are going back to work	202 (70.0)	95 (74.2)	0.58	198 (78.6)	74 (64.9)	<0.01
It is safer for HIV-positive mothers to breastfeed than to formula feed	231 (79.9)	89 (69.5)	0.63	226 (89.7)	85 (74.6)	<0.01
It is NOT very difficult for mothers to express breastmilk while they are at work or school	164 (53.0)	57 (44.5)	0.01	168 (67.1)	51 (44.7)	<0.01
If an HIV-positive mother can afford to buy formula it is NOT better for her to formula feed her baby	170 (58.8)	83 (64.9)	0.38	185 (73.4)	68 (59.7)	<0.01
Promoting breastfeeding for two years for HIV-exposed infants is NOT a risk because	197 (68.2)	85 (66.4)	0.51	210 (83.3)	79 (69.3)	<0.01

For peer review only - http://bmjopen.bmj.com/site/about/guidelines.xhtml

mothers will be able to maintain good ART adherence for that long						
Low levels of knowledge (<80%) at baseline in both groups - no significant differences between intervention and comparison groups at follow-up [concept that this relates to]						
For an HIV-positive mother on antiretroviral treatment and virally suppressed mixed feeding is better than not breastfeeding at all	39 (13.5)	15 (11.7)	0.56	57 (22.6)	16 (14.0)	0.06
In our community working mothers can successfully maintain exclusive breast feeding while going to work	219 (75.8)	89 (69.5)	0.09	208 (82.5)	92 (80.7)	0.67
There are exceptional circumstances where an HIV-positive mother would be advised not to breastfeed, such as failure of 2nd or 3rd line ART treatment, but these are not common	225 (77.9)	93 (72.7)	0.13	201 (79.7)	87 (76.3)	0.46
High levels of knowledge (≥80%) at baseline in both groups – no significant differences between intervention and comparison groups at follow-up						
HIV-exposed babies who are PCR negative must NOT stop breastfeeding as soon as possible	238 (82.4)	104 (81.3)	0.47	221 (87.7)	95 (83.3)	0.26
Exclusive breastfeeding for six months is an achievable goal for the majority of mothers	249 (86.2)	105 (82.0)	0.11	227 (90.1)	96 (84.1)	0.11
An HIV-positive mother who is on ART and not virally suppressed and is mixed feeding is putting her child at risk of acquiring HIV	256 (88.6)	109 (85.2)	0.13	219 (86.9)	97 (85.1)	0.64
In South Africa it is possible to improve exclusive breastfeeding rates	244 (84.7)	108 (84.4)	0.61	230 (91.3)	97 (85.1)	0.08
Formula feeding is NOT more convenient for a mother than breastfeeding	253 (87.5)	109 (85.2)	0.23	211 (83.7)	100(87.7)	0.32
**Number (%) participants whose attitude was to at least agree (Attitude score ≥84)***	**71 (24.6)**	**23 (17.9)**	**0.12**	**123 (49.2)**	**27 (24.1)**	**<0.01**
**Mean attitude score out of 105 (95% CI)**	**76.9 (75.9 – 77.9)**	**75.0 (73.0 – 77.0)**	**0.07**	**82.7 (81.6 – 83.8)**	**76.8 (75.0 – 78.5)**	**<0.01**

*84 was the minimum score obtainable if a participant at least agreed with all statements

For peer review only - http://bmjopen.bmj.com/site/about/guidelines.xhtml

Supplementary Table 2: Confidence statements of health workers at baseline and follow-up

Confidence statements	Number (%) who felt confident or very confident at baseline			Number (%) who felt confident or very confident at follow-up		
	Intervention (n=289)	Comparison (n=131)	p-value	Intervention (n=252)	Comparison (n=114)	p-value
Significant improvements between intervention and comparison groups at follow-up						
How confident do you feel about counselling an HIV-positive pregnant woman about how she will feed her baby	265 (91.7)	116 (88.6)	0.30	240 (95.2)	100 (87.7)	0.01
How confident do you feel about advising an HIV-positive mother about how to continue to breastfeed her baby when she returns to work or school	258 (89.3)	117 (89.3)	0.99	242 (96.0)	101 (88.6)	<0.01
How confident do you feel about advising an HIV-infected mother to continue breastfeeding for two years	216 (74.7)	105 (80.2)	0.23	236 (93.6)	93 (81.6)	<0.01
How confident do you feel about assessing ART compliance in an HIV-positive mother	240 (83.1)	111 (84.7)	0.67	230 (91.3)	94 (82.5)	0.02
How confident do you feel about advising an HIV-infected mother who is exclusively breastfeeding and has cracked nipples with bloody milk about how to feed her baby	196 (67.8)	84 (64.1)	0.45	201 (79.8)	80 (70.2)	0.04
Low levels of knowledge (<80%) at baseline in both groups - no significant differences between intervention and comparison groups at follow-up [concept that this relates to]						
How confident do you feel about advising an HIV-infected mother about how to stop breastfeeding	214 (74.1)	89 (67.9)	0.20	188 (74.6)	86 (75.4)	0.87
How confident do you feel about identifying when an HIV-positive mother is not adhering to her ART treatment	224 (77.5)	104 (79.4)	0.67	218 (86.5)	90 (79.0)	0.07
How confident do you feel about reassuring a mother living with HIV who is virally suppressed that a shorter duration of breastfeeding is better than never initiating breastfeeding	227 (78.5)	103 (78.6)	0.99	215 (85.3)	96 (84.2)	0.78
How confident do you feel about assisting a mother with HIV to safely formula feed her baby	212 (73.4)	102 (77.9)	0.33	191 (75.8)	95 (83.3)	0.11
How confident do you feel about using the guidelines for safe replacement feeding when you counsel a mother who is not adherent to ART and has a viral load above 1000 copies/ml	191 (66.1)	99 (75.6)	0.05	185 (73.4)	75 (65.8)	0.14
How confident do you feel about advising an HIV-infected mother who is exclusively breastfeeding and has defaulted from her ART about how to feed her baby	205 (70.9)	97 (74.1)	0.51	189 (75.0)	80 (70..2)	0.33
How confident do you feel about managing poor ART compliance in an HIV-infected breastfeeding mother	215 (74.4)	104 (79.4)	0.27	209 (82.9)	89 (78.1)	0.27

For peer review only - http://bmjopen.bmj.com/site/about/guidelines.xhtml

A mother is not adherent to ART and her last viral load is 1000 copies per ml. How confident do you feel about counselling her about feeding her infant?	199 (68.9)	98 (74.8)	0.21	198 (78.6)	80 (70.2)	0.08
High levels of knowledge (≥80%) at baseline in both groups – no significant differences between intervention and comparison groups at follow-up						
How confident do you feel about giving information about the risks and benefits of breastfeeding to an HIV-infected mother	268 (92.7)	120 (91.6)	0.67	238 (94.4)	102 (89.5)	0.09
How confident do you feel about assessing whether there is good positioning and attachment during breastfeeding	263 (91.0)	125 (95.4)	0.11	243(96.4)	107 (93.9)	0.27
How confident do you feel about advising an HIV-infected mother who is virally suppressed who is mixed feeding her infant	243 (84.1)	113 (86.3)	0.57	226 (89.7)	99 (86.8)	0.43
How confident do you feel about advising an HIV-positive mother about starting complementary feeds	251 (86.9)	115 (87.8)	0.79	229 (90.9)	103 (90.4)	0.87
How confident do you feel about explaining the risks of HIV transmission through breastmilk to an HIV-infected mother with high viral load	247 (85.5)	114 (87.0)	0.67	220 (87.3)	105 (92.1)	0.17
How confident do you feel about explaining to a mother about expressing and storing milk	269 (93.1)	124 (94.7)	0.54	237 (94.1)	109 (95.6)	0.54
**Number (%) participants who were confident or very confident (Sum Score ≥ 57)**	**164 (56.8)**	**86 (67.2)**	**0.09**	**175 (70.0)**	**72 (64.3)**	**0.28**
**Mean confidence score out of 76 (95% CI)**	**59.1 (58.0-60.2)**	**59.1 (57.0-61.3)**	**1.0**	**61.2 (60.8-63.1)**	**59.9 (58.1-61.7)**	**0.05**

For peer review only - http://bmjopen.bmj.com/site/about/guidelines.xhtml

Version4_ 14August2017

QUESTIONNAIRE FOR HEALTHCARE WORKERS

For peer review only

For peer review only - http://bmjopen.bmj.com/site/about/guidelines.xhtml

Version4_ 14August2017

**Section 1. Admin**		
1.1.	Interview Date	☐☐/ ☐☐ ☐ /☐☐☐☐<br>D D M M M Y Y Y Y
1.2.	District	1 OTshwane<br>2 OUgu
1.3.	Sub district	**If Tshwane chosen above:**<br>1 OTshwane 1<br>1 OTshwane 2<br>1 OTshwane 3<br>1 OTshwane 6<br>**If Ugu chosen above:**<br>1 O Omdoni<br>1 O UmZumbe<br>1 O Hibiscus<br>1 O uMuziwabantu
1.4.	Facility	**If Tshwane 1 selected the following clinics can be chosen:**<br>1 O Soshang Block JJ clinic<br>1 O KT Motubatse clinic<br>1 O Boikhutsong clinic<br>1 O Sedilega clinic<br>1 O Shoshanguve 2 clinic<br>1 O Soshang Block TT clinic<br>**If Tshwane 2 selected:**<br>1 O Jubilee gateway clinic<br>1 O Kekanastad clinic<br>1 O Suurman clinic<br>1 O Ramotse clinic<br>1 O Kekana gardens clinic<br>1 O New Eersterus clinic<br>**If Tshwane 3 selected**<br>Atteridgeville Clinic<br>Bophelong Clinic (Tshw 3)<br>Saulsville Clinic

For peer review only

Version4_ 14August2017

		**If Tshwane 6 selected**<br>S Bopape CHC<br>Eersterust CHC<br>Nellmapius Clinic<br><br>**If Omdoni selected**<br>GJ Crooke's Gateway<br>Pennington Clinic<br>Philani Clinic<br>Scottburgh Clinic<br>Umzinto Clinic<br><br>**If UmZumbe selected**<br>• Gqayinyanga clinic<br>• St Faiths clinic<br>• Phungashe clinic<br>• Ntimbankulu clinic<br>• Turton CHC<br>• Ndelu clinic<br><br>**If Hibiscus coast selected:**<br>• Gamalakhe CHC<br>• Southport Clinic<br>• Marburg Clinic<br><br>**If uMuziwabantu selected**<br>• Santombe clinic<br>• Meadowsweet clinic<br>• Mbonwa clinic
1.5.	Health worker number	Assigned number to each participant

For peer review only

Version4_ 14August2017

Section 2. Demographics			
2.1.	What is your date of birth?	☐☐/☐☐/☐☐☐☐<br>D D M M Y Y Y Y	
2.2.	Gender	1 O Male	2 O Female
2.3.	What is your role in this clinic?	1 O Lay counsellor or nutritional advisor<br>2.O Enrolled nurse assistant<br>3.O Enrolled nurse<br>4.O Registered nurse<br>5.O Medical degree (MB ChB or equivalent)<br>6.O community health worker (CCG)<br>7 O: dietician<br>8 O: Registered nurse operational manager<br>9 O: other specify below	
2.4.	Other		
2.5.	How long have you been working as a health worker?	1 O less than 1 year<br>2 O 1- <2 years<br>3 O 2- <5 years<br>4 O 5-< 10 years<br>5 O 10 or more years	

For peer review only

Version4_ 14August2017

Section 3	**Updated HIV and infant feeding guidelines.**<br>**In this section you will be asked about new infant feeding guidelines adopted in South Africa.**		
3.1	During 2017 have you received any information or training at work about the revised Infant and Young Child Feeding Policy – in the form of a circular, letter, workshop, meeting or lecture	1O Yes	0O No SKIP
3.2	If yes, how did you receive this information?	1. O Circular/letter<br>2.O Meeting<br>3.O Workshop<br>4.O Feedback/information from colleague<br>5.O lecture<br>6.O other	
3.3	Who gave you this information/ training?	1 O District trainer/ DoH staff member<br>2 O Outside/ private company	
3.4	How long was this training?	__________hours	

For peer review only

Version4_ 14August2017

**SECTION 4.**	**TRAINING Topics**<br>Have you received any training on the following topics (either in-service or formal training).<br><br>**Usuke wathola uqeqesho kulezihloko ezilandelayo?**		
4.1	Did the content of your training include the importance of breastfeeding in preventing common childhood illness such as diarrhoea?	1 ○ Yes	0○ No
4.2	Have you ever had any training about correct positioning and attachment of an infant during breastfeeding?	1 ○ Yes	0○ No
4.3	Have you ever had any training about the management of common breastfeeding problems?	1 ○ Yes	0○ No
4.4	Have you ever had any training about advising a mother about how to provide breastmilk for her baby when she returns to work or school	1 ○ Yes	0○ No
4.5	Have you ever had any training about how to advise a mother about formula feeding safely?	1 ○ Yes	0○ No
4.6	Have you ever had any training about how to advise an HIV infected woman about how to feed her baby?	1 ○ Yes	0○ No
4.7	Have you ever had any training about how to manage breastfeeding problems in HIV infected women (cracked nipples, mastitis etc.)?	1 ○ Yes	5.0 ○ No
4.8	Have you ever had any training about how to assess and support ART adherence for HIV infected women?	1 ○ Yes	0 ○ No
4.9	Have you ever had any training on viral load monitoring?	1 ○ Yes	0 ○ No

For peer review only

For peer review only - http://bmjopen.bmj.com/site/about/guidelines.xhtml

Version4_ 14August2017

Section 5	ACTIVITIES:<br><br>***Think carefully about your work in this facility.*** For the activity mentioned consider whether you ever perform this activity and if so how regularly do you perform this activity? If you do not perform this activity at all select the option 'Never'				
5.1	How often do you talk to groups of pregnant women attending the antenatal clinic about infant feeding (group counselling)	1 O one or more times per week	2 O one to three times per month	3 O Less than once a month	4 O Never
5.2	How often do you talk to a pregnant woman individually about her plan for feeding her baby	1 O one or more times per week	2 O one to three times per month	3 O Less than once a month	4 O Never
5.3	How often do you talk to an HIV infected pregnant woman about her plan for feeding her baby	1 O one or more times per week	2 O one to three times per month	3 O Less than once a month	4 O Never
5.4	How often do you assist a mother with breastfeeding within the first hour post delivery	1 O one or more times per week	2 O one to three times per month	3 O Less than once a month	4 O Never
5.5	How often do you talk to a mother about how she is feeding her baby?	1 O one or more times per week	2 O one to three times per month	3 O Less than once a month	4 O Never
5.6	How often do you observe a mother breastfeeding during a clinic or home visit	1 O one or more times per week	2 O one to three times per month	3 O Less than once a month	4 O Never
5.7	How often do you talk to a mother about positioning and attachment of the baby during breastfeeding	1 O one or more times per week	2 O one to three times per month	3 O Less than once a month	4 O Never
5.8	How often do you talk to an HIV infected mother about managing a breastfeeding problem (e.g. cracked nipples, baby crying all the time, mother says she does not have enough milk)	1 O one or more times per week	2 O one to three times per month	3 O Less than once a month	4 O Never
5.9	How often do you talk to a mother about how to maintain breastfeeding when away from the baby (going back to school or work)	1 O one or more times per week	2 O one to three times per month	3 O Less than once a month	4 O Never
5.10	How often do you talk to an HIV infected breastfeeding mother about taking ARVs	1 O one or more times per week	2 O one to three times per month	3 O Less than once a month	4 O Never

For peer review only - http://bmjopen.bmj.com/site/about/guidelines.xhtml

Version4_ 14August2017

SECTION 6	INFANT FEEDING KNOWLEDGE			
please state whether the statement is true or false or you do not know				
	**Statement**	**True**	**False**	**Do not know**
6.1	Exclusive breastfeeding is the recommended infant feeding method for ALL infants aged 0-6 months in SA, regardless of mother's HIV status	1 ○	2 ○	3 ○
6.2	Giving any formula milk during the first six months of life increases the risk of death from diarrhoea and/or pneumonia	1 ○	2 ○	3 ○
6.3	Continued breastfeeding for 2 years is the recommended infant method in SA for ALL children, regardless of mother's HIV status	1 ○	2 ○	3 ○
6.4	Mothers living with HIV who are receiving antiretroviral treatment and are virally suppressed should be advised not to breastfeed their infants	1 ○	2 ○	3 ○
6.5	When an HIV infected mother is ready to add complementary feeds she should stop breastfeeding rapidly over a 24hour period	1 ○	2 ○	3 ○
6.6	If an HIV exposed baby is receiving both breastmilk and formula milk, the mother should chose to either breastfeeding or formula feeding if she is adherent to ART	1 ○	2 ○	3 ○
6.7	A mother who is working and giving formula milk should mix the milk herself and leave for the carer to give during the day	1 ○	2 ○	3 ○
6.8	An HIV positive mother who is virally suppressed on antiretroviral treatment should breastfeed her child rather than not breastfeed to improve the child's survival	1 ○	2 ○	3 ○
6.9	When sterilising feeding bottles cover the bottles with water in a saucepan and place on the heat. As soon as the water boils remove from heat and leave the bottle in the water until completely cool	1 ○	2 ○	3 ○
6.10	In South Africa, the leading cause of death amongst children under 5 is pneumonia	1 ○	2 ○	3 ○
6.11	In South Africa, HIV infected women who are breastfeeding should be supported to adhere to antiretroviral treatment and should be counselled and supported to exclusively breastfeed their infants for the first six months of life whilst maintaining an undetectable viral load	1 ○	2 ○	3 ○
6.12	A baby under 4 months should be given soft porridge once he/she seems hungry	1 ○	2 ○	3 ○

For peer review only - http://bmjopen.bmj.com/site/about/guidelines.xhtml

Version4_ 14August2017

6.13	A mother living with HIV and adherent to antiretroviral treatment cannot exclusively breastfeed her 4-month old infant because she is working. It is better for this mother to give formula during the day and breastfeed at night rather than giving no breast milk at all	1 ○	2 ○	3 ○
6.14	An HIV exposed baby who is exclusively breastfeeding should be given some water when the weather is very hot	1 ○	2 ○	3 ○
6.15	If a baby has a positive PCR (HIV test) at birth the mother should stop breastfeeding if this is affordable and feasible in her situation	1 ○	2 ○	3 ○
6.16	Giving a baby expressed breastmilk is not as good as breastfeeding	1 ○	2 ○	3 ○
6.17	If a mother misses 2 doses of her ART in one month, she should be classified as a treatment failure	1 ○	2 ○	3 ○
6.18	An HIV positive mother who has cracked nipples should continue to breastfeed unless they are bleeding	1 ○	2 ○	3 ○
6.19	A mother who has missed 6 tablets of FDC in one month is considered to be poorly adherent and should stop breastfeeding immediately	1 ○	2 ○	3 ○
6.20	There are long term health benefits of breastfeeding for mother and child that last beyond the breastfeeding period	1 ○	2 ○	3 ○
6.21	In South Africa, HIV infected women who are breastfeeding should be supported to adhere to antiretroviral treatment and should introduce complementary foods around 6 months and be supported to continue breastfeeding for at least two years.	1 ○	2 ○	3 ○
6.22	It is safe to give the baby expressed breastmilk that has been kept outside the fridge for 8 hours	1 ○	2 ○	3 ○

For peer review only

For peer review only - http://bmjopen.bmj.com/site/about/guidelines.xhtml

Version4_ 14August2017

SECTION 7.		INFANT FEEDING ATTITUDE<br><br>Please state whether you completely disagree, disagree, neutral, agree or completely agree with the statement				
		Completely disagree	Disagree	Neutral	Agree	Completely agree
7.1	There have been so many changes to the infant feeding guidelines and breastfeeding guidelines that I am confused about what to tell mothers who are HIV infected about	1 ○	2 ○	3 ○	4 ○	5 ○
7.2	When a baby cries all the time it is usually because the baby is hungry and needs more food than just breastmilk	1 ○	2 ○	3 ○	4 ○	5 ○
7.3	Exclusive breastfeeding in the first 6 months of life is the best choice for all mothers and babies in South Africa	1 ○	2 ○	3 ○	4 ○	5 ○
7.4	For an HIV exposed infant any breastfeeding is better than no breastfeeding at all, as long as the mother is virally suppressed and on antiretroviral therapy	1 ○	2 ○	3 ○	4 ○	5 ○
7.5	The benefits of breastfeeding for protecting children from illness such as diarrhoea and pneumonia outweighs the risk of acquiring HIV if the mother is on antiretroviral treatment	1 ○	2 ○	3 ○	4 ○	5 ○
7.6	I feel that an HIV infected mother who has not disclosed to her partner is at high risk of non-adherence to ART and should stop breastfeeding as soon as [illegible]	1 ○	2 ○	3 ○	4 ○	5 ○
7.7	I should support all mothers, regardless of HIV status, to continue breastfeeding until 2 years, as long as HIV infected women are virally suppressed	1 ○	2 ○	3 ○	4 ○	5 ○
7.8	I should advise an HIV positive virally suppressed mother who has cracked and bleeding nipples to temporarily stop breastfeeding	1 ○	2 ○	3 ○	4 ○	5 ○
7.9	HIV exposed babies who are PCR negative must stop breastfeeding as soon as possible	1 ○	2 ○	3 ○	4 ○	5 ○

For peer review only

For peer review only - http://bmjopen.bmj.com/site/about/guidelines.xhtml

Version4_ 14August2017

7.10	Formula feeding is the best choice for mothers living in good socioeconomic circumstances who are going back to work	1 O	2 O	3 O	4 O	5 O
7.11	For an HIV positive mother on antiretroviral treatment and virally suppressed mixed feeding is better than not breastfeeding at all	1 O	2 O	3 O	4 O	5 O
7.12	Exclusive breastfeeding for six months is an achievable goal for the majority of mothers	1 O	2 O	3 O	4 O	5 O
7.13	It is safer for HIV positive mothers to breastfeed than to formula feed	1 O	2 O	3 O	4 O	5 O
7.14	In our community working mothers can successfully maintain exclusive breast feeding while going to work	1 O	2 O	3 O	4 O	5 O
7.15	An HIV positive mother who is on ART and not virally suppressed and is mixed feeding is putting her child at risk of acquiring HIV	1 O	2 O	3 O	4 O	5 O
7.16	It is very difficult for mothers to express breastmilk while they are at work or school	1 O	2 O	3 O	4 O	5 O
7.17	If an HIV positive mother can afford to buy formula it is better for her to formula feed her baby	1 O	2 O	3 O	4 O	5 O
7.18	Promoting breastfeeding for two years for HIV exposed infants is a risk because mothers will be unable to maintain good ART adherence for that long	1 O	2 O	3 O	4 O	5 O
7.19	In South Africa it is possible to improve exclusive breastfeeding rates	1 O	2 O	3 O	4 O	5 O
7.20	There are exceptional circumstances where an HIV positive mother would be advised not to breastfeed, such as failure of 2nd or 3rd line ART treatment, but these are not common	1 O	2 O	3 O	4 O	5 O
7.21	Formula feeding is more convenient for a mother than breastfeeding	1 O	2 O	3 O	4 O	5 O

For peer review only - http://bmjopen.bmj.com/site/about/guidelines.xhtml

Version4_ 14August2017

**SECTION 8.**	**INFANT FEEDING COUNSELLING CONFIDENCE**<br><br>For each activity below, please indicate how confident you feel to undertake each activity. Do you feel "Not at all confident", "Not very confident", "Somewhat confident" or "Very confident".				
		Not at all confident	Not very confident	Confident	Very confident
8.1	How confident do you feel about counselling an HIV positive pregnant woman about how she will feed her baby	1 ○	2 ○	3 ○	4 ○
8.2	How confident do you feel about giving information about the risks and benefits of breastfeeding to an HIV infected mother	1 ○	2 ○	3 ○	4 ○
8.3	How confident do you feel about assessing whether there is good positioning and attachment during breastfeeding	1 ○	2 ○	3 ○	4 ○
8.4	How confident do you feel about advising an HIV positive mother about how to continue to breastfeed her baby when she	1 ○	2 ○	3 ○	4 ○
8.5	How confident do you feel about advising an HIV infected mother who is virally suppressed who is mixed feeding her infant	1 ○	2 ○	3 ○	4 ○
8.6	How confident do you feel about advising an HIV infected mother to continue breastfeeding for two years	1 ○	2 ○	3 ○	4 ○
8.7	How confident do you feel about advising an HIV infected mother about how to stop breastfeeding	1 ○	2 ○	3 ○	4 ○
8.8	How confident do you feel about advising an HIV positive mother about starting complementary feeds	1 ○	2 ○	3 ○	4 ○
8.9	How confident do you feel about assessing ART compliance in an HIV positive mother	1 ○	2 ○	3 ○	4 ○
8.10	How confident do you feel about identifying when an HIV positive mother is not adhering to her ART treatment	1 ○	2 ○	3 ○	4 ○
8.11	How confident do you feel about reassuring a mother living with HIV who is virally suppressed that a shorter duration of breastfeeding is better than never initiating breastfeeding	1 ○	2 ○	3 ○	4 ○
8.12	How confident do you feel about explaining the risks of HIV transmission through breastmilk to an HIV infected mother with high viral load	1 ○	2 ○	3 ○	4 ○

For peer review only

For peer review only - http://bmjopen.bmj.com/site/about/guidelines.xhtml

Version4_ 14August2017

8.13	How confident do you feel about assisting a mother with HIV to safely formula feed her baby	1 O	2 O	3 O	4 O
8.14	How confident do you feel about advising an HIV infected mother who is exclusively breastfeeding and has cracked nipples with bloody milk about how to feed her baby	1 O	2 O	3 O	4 O
8.15	How confident do you feel about using the guidelines for safe replacement feeding when you counsel a mother who is not adherent to ART and has a viral load above 1000 copies/ml	1 O	2 O	3 O	4 O
8.16	How confident do you feel about advising an HIV infected mother who is exclusively breastfeeding and has defaulted from her ART about how to feed her baby	1 O	2 O	3 O	4 O
8.17	How confident do you feel about explaining to a mother about expressing and storing milk	1 O	2 O	3 O	4 O
8.18	How confident do you feel about managing poor ART compliance in an HIV infected breastfeeding mother	1 O	2 O	3 O	4 O
8.19	A mother is not adherent to ART and her last viral load is 1000 copies per ml. How confident do you feel about counselling her about feeding her infant?	1 O	2 O	3 O	4 O

For peer review only

For peer review only - http://bmjopen.bmj.com/site/about/guidelines.xhtml
